# Isoquinoline Alkaloids as Protein Tyrosine Phosphatase Inhibitors from a Deep-Sea-Derived Fungus *Aspergillus puniceus*

**DOI:** 10.3390/md20010078

**Published:** 2022-01-17

**Authors:** Cheng-Mei Liu, Fei-Hua Yao, Xin-Hua Lu, Xue-Xia Zhang, Lian-Xiang Luo, Xiao Liang, Shu-Hua Qi

**Affiliations:** 1 CAS Key Laboratory of Tropical Marine Bio-Resources and Ecology, Guangdong Key Laboratory of Marine Materia Medica, Innovation Academy of South China Sea Ecology and Environmental Engineering, South China Sea Institute of Oceanology, Chinese Academy of Sciences, Guangzhou 510301, China; liuchengmei19@mails.ucas.ac.cn (C.-M.L.); yaofeihua20@malls.ucas.ac.cn (F.-H.Y.); liangxiao@scsio.ac.cn (X.L.); 2 University of Chinese Academy of Sciences, Beijing 100049, China; 3 Southern Marine Science and Engineering Guangdong Laboratory, 1119 Haibin Road, Guangzhou 511458, China; 4 New Drug Research & Development Co., Ltd., North China Pharmaceutical Group Corporation, Shijiazhuang 050015, China; luxinhua89@hotmail.com (X.-H.L.); zhangxuexiazxx@163.com (X.-X.Z.); 5 The Marine Biomedical Research Institute, Guangdong Medical University, Zhanjiang 524023, China; luolianxiang321@gdmu.edu.cn

**Keywords:** deep-sea-derived fungus, *Aspergillus puniceus*, isoquinoline alkaloid, protein tyrosine phosphatase inhibitor, cytotoxicity, antibacterial

## Abstract

Puniceusines A–N (**1**–**14**), 14 new isoquinoline alkaloids, were isolated from the extracts of a deep-sea-derived fungus, *Aspergillus puniceus* SCSIO z021. Their structures were elucidated by spectroscopic analyses. The absolute configuration of **9** was determined by ECD calculations, and the structures of **6** and **12** were further confirmed by a single-crystal X-ray diffraction analysis. Compounds **3**–**5** and **8**–**13** unprecedentedly contained an isoquinolinyl, a polysubstituted benzyl or a pyronyl at position C-7 of isoquinoline nucleus. Compounds **3** and **4** showed selective inhibitory activity against protein tyrosine phosphatase CD45 with IC_50_ values of 8.4 and 5.6 µM, respectively, **4** also had a moderate cytotoxicity towards human lung adenocarcinoma cell line H1975 with an IC_50_ value of 11.0 µM, and **14**, which contained an active center, -C=N^+^, exhibited antibacterial activity. An analysis of the relationship between the structures, enzyme inhibitory activity and cytotoxicity of **1**–**14** revealed that the substituents at C-7 of the isoquinoline nucleus could greatly affect their bioactivity.

## 1. Introduction

Isoquinoline alkaloids are a large group of alkaloids in the plant kingdom, showing diverse pharmacological and biological activities such as anticancer, anti-inflammatory, cholesterol-lowering, antihyperglycemic, antiplasmodial, antifungal, and antimicrobial activity, etc. [1]. According to their structural components, isoquinoline and tetrahydroisoquinoline alkaloids can be classified into over 20 sub-classes, mainly including simple isoquinoline, benzylisoquinoline, bisbenzylisoquinoline, proto-berberine alkaloid, and aporphine alkaloid, etc. [1]. The most famous representative of this group is antidiabetic berberine. However, only a few isoquinoline alkaloids have been isolated from fungi, such as TMC-120A, B and C from *Aspergillus ustus* [2] and *A. insuetus* [3]; chaetoindicins A–C from *Chaetomium indicum* [4]; fusarimine from *Fusarium* sp. [5]; 8-methoxy-3,5-dimethylisoquinolin-6-ol from *Penicillium citrinum* [6]; azaphilone from *P. sclerotiorum* [7]; and spathullins A–B from *P. spathulatum* [8].

**Figure 1:**
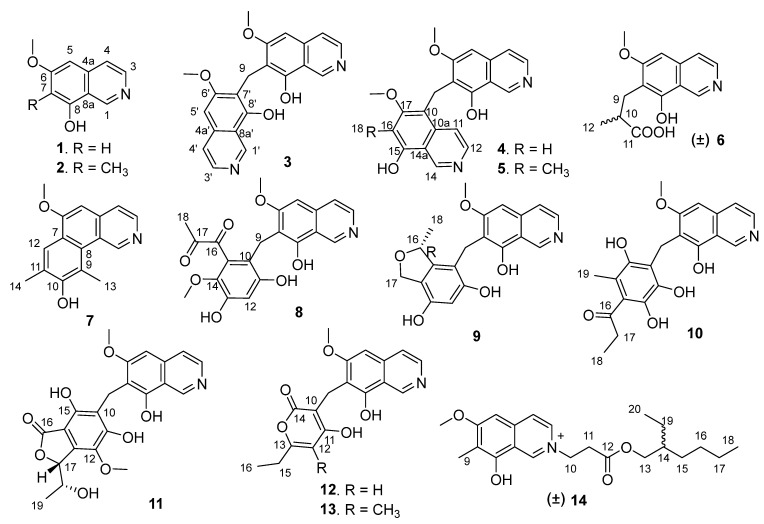
Structures of the isolated compounds **1**–**14**.

Malfunctions in protein tyrosine phosphatase (PTP) activity are linked to various diseases, ranging from cancer to neurological disorders and diabetes, such as CD45, SHP1, TCPTP, PTP1B and LAR linked to cancer, CD45, SHP1 and LAR also linked to neurological diseases, and PTP1B and LAR also linked to diabetes, etc. [9]. Some PTPs emerged as promising targets for therapeutic intervention in recent years [9,10]. However, achieving the selectivity of PTP inhibitors is a big challenge. In recent years, we obtained a series of natural compounds including anthraquinones [11], meroterpenoids [12], xanthone-type and anthraquinone-type mycotoxins [13], and oxaphenalenones [14] as PTP inhibitors from marine-derived fungi. In order to further explore diversified bioactive compounds from the deep-sea-derived fungus, *Aspergillus puniceus* SCSIO z021, we changed culture media and further conducted a chemical investigation of this strain cultured with a complex medium, which led to the characterization of 14 undescribed isoquinoline alkaloids, puniceusines A–N (**1**–**14**) (Figure 1). Compounds **1**–**14** were evaluated for their enzyme inhibitory activity against five kinds of PTPs, cytotoxicity towards human lung adenocarcinoma cell line H1975, and antibacterial activity. Herein, we report the isolation, structure elucidation and bioactivities of **1**–**14**.

## 2. Results and Discussion

Puniceusine A (**1**) has the molecular formula C_10_H_9_NO_2_, as determined by HRESIMS. The ^1^H NMR spectrum (Table 1) showed the presence of one methoxy group at *δ*_H_ 3.96 (3H, s) and five aromatic hydrogens at *δ*_H_ 9.45 (1H, s), 8.40 (1H, d, *J* = 5.7 Hz), 8.06 (1H, d, *J* = 6.4 Hz), 7.13 (1H, s), 6.81 (1H, s). The ^13^C NMR spectrum (Table 2) showed 10 carbon signals including one methoxy, five aromatic methines, and four aromatic non-protonated carbons. These NMR data were similar to those of 6,8-dimethoxyisoquinolin [15] and papraline [16], and the only clear difference between **1** and 6,8-dimethoxyisoquinolin was the disappearance of one oxygenated methyl, which indicated that **1** was also an isoquinoline alkaloid. This was supported by the HMBC spectrum showing correlations from H-1 to C-3/C-4a/C-8a, H-3 to C-4/C-4a, H-4 to C-3/C-5/C-8a, H-5 to C-4/C-6/C-7/C-8a, and H-7 to C-5/C-6/C-8/C-8a. In addition, the HMBC correlation from δ_H_ 3.96 (3H, s) to C-6 suggested that a methoxy group was attached at C-6. Thus, the structure of **1** was determined to be 6-methoxy-8-hydroxy-isoquinolin.

Puniceusine B (**2**) was assigned the molecular formula C_11_H_11_NO_2_ by HRESIMS. The ^1^H NMR and ^13^C NMR data (Table 1 and Table 2) showed great similarity to those of **1**, and the main difference between them was the additional presence of one methyl (δ_H_ 2.25, 3H, s; δ_C_ 9.2) and the disappearance of one aromatic hydrogen in **2**. The HMBC correlations from δ_H_ 2.25 to C-6/C-7/C-8 suggested the additional methyl attached at C-7. Hence, the structure of **2** was determined to be 6-methoxy-7-methyl-8-hydroxy-isoquinolin.

**Figure 2:**
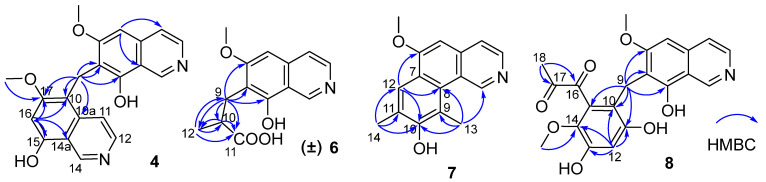
Key HMBC correlations of compounds **4**, **6**–**8**.

Puniceusine C (**3**) was found to have the molecular formula C_21_H_18_N_2_O_4_ by HRESIMS that was nearly twice that of **2**. The ^1^H and ^13^C NMR data (Table 1 and Table 2) showed great similarity to those of **2**, and the clearest difference between them was the disappearance of a methyl signal and the additional presence of a methylene signal (*δ*_H_ 4.32, 2H, s; δ_C_ 19.4) in **3**. The ^1^H NMR spectrum of **3** showed a double increase in the integral areas for H-1, H-3, H-4, H-5, and a methoxy group. The HMBC correlations (Figure 2) from *δ*_H_ 4.32 (H_2_-9) to C-6/C-7/C-8 suggested a methylene instead of a methyl attached at C-7. These data indicated that **3** was a symmetrical dimer of **1**, connected at positions C-7′ and C-7 by a methylene C-9. Thus, the structure of **3** was determined as shown.

Puniceusine D (**4**) showed the same molecular formula of C_21_H_18_N_2_O_4_ as that of **3** by analysis of its HRESIMS and NMR data (Table 1 and Table 2). The ^1^H NMR spectrum showed the presence of two downfield hydrogens at δ_H_ 9.59 (1H, s) and 9.52 (1H, s); six aromatic hydrogens at δ_H_ 8.58 (1H, d, *J* = 7.0 Hz), 8.31 (1H, d, *J* = 7.8 Hz), 8.29 (1H, d, *J* = 7.0 Hz), 8.05 (1H, d, *J* = 6.5 Hz), 7.11 (1H, s), and 7.00 (1H, s); two methoxys groups at δ_H_ 3.79 (3H, s) and 3.98 (3H, s); and one methylene at δ_H_ 4.47 (2H, s). The ^13^C NMR spectrum showed 21 carbon signals including one methylene, two methoxyls, eight aromatic methines, and ten aromatic non-protonated carbons. These data showed similarity to those of **1**–**3**, which indicated that **4** was also a dimer of **1**. The HMBC correlations from H-5 to C-4/C-8a, from H-16 to C-10/C-14a/C-15/C-17, and from H_2_-9 to C-6/C-7/C-8/C-10/C-10a/C-17 (Figure 2) suggested that **4** was an asymmetric dimer of **1** connected at positions C-7 and C-10 by a methylene C-9. The two methoxy groups were attached at C-6 and C-17 based on the HMBC correlations of δ_H_ 3.98 (3H, s) with C-17 and δ_H_ 3.79 (3H, s) with C-6, respectively. Therefore, the structure of **4** was established as shown.

The molecular formula of puniceusine E (**5**) was determined as C_22_H_21_N_2_O_4_ by HRESIMS. The ^1^H and ^13^C NMR data (Table 1 and Table 2) of **5** were greatly similar to those of **4**, and the only obvious difference between them was the absence of one aromatic hydrogen and the additional presence of one methyl signal (δ_H_ 2.38, 3H, s; δ_C_ 10.8) in **5**. The HMBC correlations from H_3_-18 (δ_H_ 2.38) to C-15/C-16/C-17 suggested a methyl located at C-16 instead of a hydrogen. Thus, the structure of **5** was established as shown.

**Figure 3:**
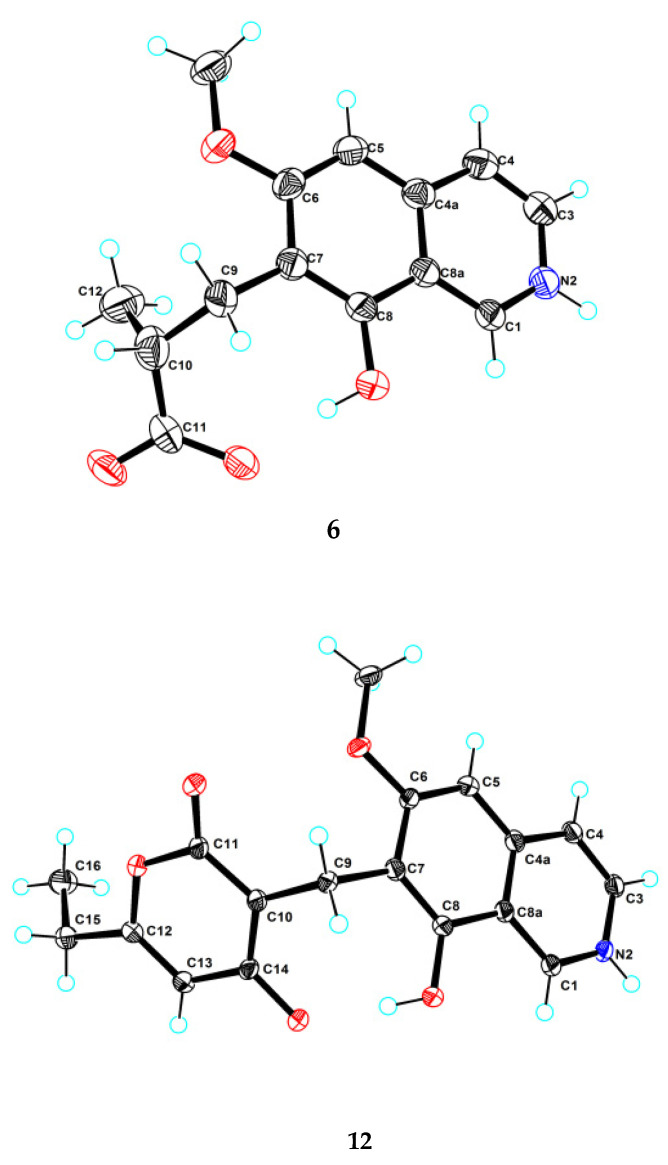
Ortep plot of the X-ray crystallographic data for **6** and **12**.

Puniceusine F (**6**) had the molecular formula C_14_H_15_NO_4_, as determined by HRESIMS. The ^1^H and ^13^C NMR data (Table 1 and Table 2) of **6** were similar to those of **1**, and the clearest difference between them was the disappearance of one aromatic hydrogen and the additional presence of one methyl (*δ*_H_ 1.27, d, *J* = 7.5 Hz, 3H), one methylene, one methine, and one carboxyl group in **6**. The HMBC correlations (Figure 2) from H_2_-9 (*δ*_H_ 2.97 (dd, *J* = 14.0, 5.5 Hz, 1H), 3.17 (dd, *J* = 14.0, 8.5 Hz, 1H)) to C-6/C-7/C-8/C-10/C-11/C-12, from H-10 (*δ*_H_ 2.85, m, 1H) to C-11/C-12, and from H_3_-12 (*δ*_H_ 1.27, d, *J* =7.5 Hz, 3H) to C-9/C-10/C-11 suggested an isobutyric acid group attached at C-7 of the isoquinoline nucleus. The optical rotation and measured CD data of **6** were zero, which indicated that **6** was a racemic mixture. This was supported by the HPLC analysis of **6** with a chiral column (CHIRALPAK IA 4.6 mm × 250 mm column) eluting with n-hexane/ethanol/TFA (68:32:0.2, *v*/*v*) (Figure S39). This structure was further confirmed by a single-crystal X-ray diffraction analysis (Figure 3).

The molecular formula of puniceusine G (**7**) was determined to be C_16_H_15_NO_2_, according to its HRESIMS. The ^1^H and ^13^C NMR data (Table 1 and Table 2) of **7** were similar to those of **1**, and the clearest difference between them was the additional presence of two double bonds (*δ*_C_ 112.0 (C, C-9), 115.0 (CH, C-12), 120.7 (C, C-11), 148.7 (C, C-10)) and two methyls (*δ*_H_ 2.51 (3H, s, H-14), 2.93 (3H, s, H-13); *δ*_C_ 8.0, 8.4) in **7**. The HMBC correlations (Figure 2) from H-12 to C-6/C-8/C-10, from H_3_-13 to C-1/C-8/C-9/C-10, and from H_3_-14 to C-10/C-11/C-12, suggested a 3,5-dimethyl-4-hydroxyphenyl group attached at the isoquinoline nucleus by sharing C-7 and C-8 to form a benzo[*h*]isoquinoline unit. Therefore, the structure of **7** was established as shown.

Puniceusine H (**8**) had the molecular formula of C_21_H_19_NO_7_ as determined by its HRESIMS. The ^1^H and ^13^C NMR data (Table 2 and Table 3) of **8** showed similarity to those of **4**–**6** with the presence of characteristic chemical shifts for a methylene C-9 (δ_H_ 4.01 (2H, s), δ_C_ 19.3 (CH_2_)). The HMBC correlations (Figure 2) from H_2_-9 to C-6/C-7/C-8/C-10/C-11/C-15, from H-12 (δ_H_ 6.58, 1H, s) to C-10/C-11/C-13/C-14, and from H_3_-19 (δ_H_ 3.52, 3H, s) to C-14 suggested a 2,4-dihydroxy-5-methoxyphenyl fragment connected with isoquinoline unit by a methylene at position C-7. In addition, the HMBC correlations from H_3_-18 (δ_H_ 2.30, 3H, s) to C-16 (δ_C_ 195.1, C)/C-17 (δ_C_ 196.9, C) suggested the presence of a 1,2-propanedione group that was exclusively assigned to attach at C-15 of the benzene ring based on the above data. Thus, the structure of **8** was established as shown.

**Figure 4:**
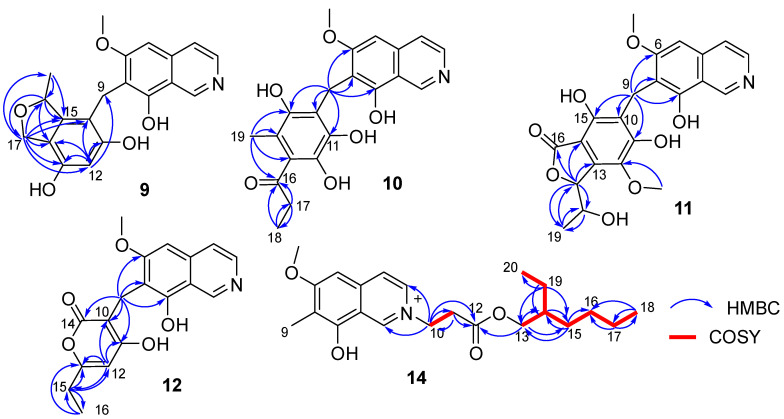
Key COSY and HMBC correlations of compounds **9**–**12** and **14**.

Puniceusine I (**9**) had the molecular formula of C_20_H_19_NO_5_ according to its HRESIMS with 12 degrees of unsaturation. The ^1^H and ^13^C NMR data (Table 3 and Table 4) of **9** were similar to those of **8**, and the clearest difference between them was the disappearance of two keto carbons and one methoxy and the additional presence of one oxygenated methylene (δ_H_ 4.84 (1H, dd, *J* = 11.5, 2.5 Hz), 4.69 (1H, dd, *J* = 11.5 Hz), δ_C_ 68.9, CH_2_) and one oxygenated methine (δ_H_ 5.22 (1H, m), δ_C_ 79.3, CH). A detailed analysis of HSQC and HMBC spectra proved that **9** also contained a 1,2,4,5,6-pentasubstituted-benzyl attached at C-7 of isoquinoline nucleus. In addition, the HMBC correlations from H-16 to C-14/C-15/C-17, from H_2_-17 to C-10/C-11/C-12/C-13/C-14/C-15/C-16/C-18, from H_3_-18 (δ_H_ 1.35, d, *J* = 6.2 Hz, 3H) to C-15/C-16 (Figure 4), suggested a 2-methyl-2,5-dihydrofuran ring connected with the benzene ring via C-14 and C-15. Therefore, the 2D structure of **9** was established as shown. The absolute configuration of **9** was further determined by electronic circular dichroism (ECD) calculations (Tables S1–S3).

**Figure 5:**
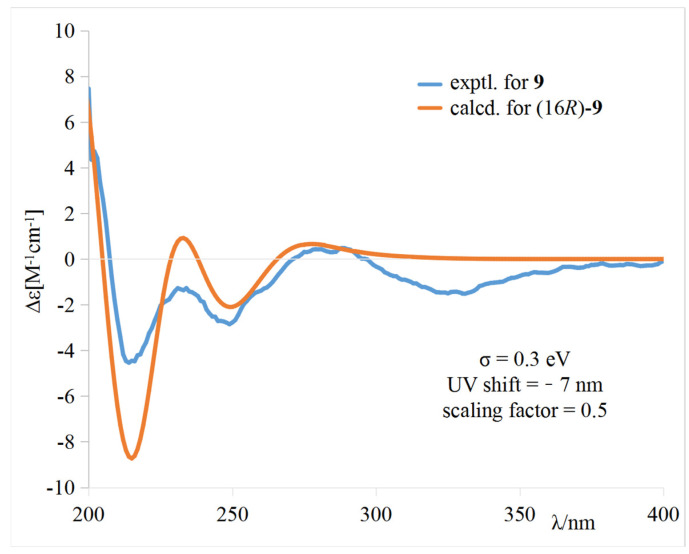
Comparison of the experimental and calculated ECD spectra of **9** in CH_3_OH.

The calculated weighted ECD spectrum of (16*R*)-**9** agreed well with the experimental ECD spectrum of **9** (Figure 5), leading to the assignment of the absolute configuration at C-16.

Puniceusine J (**10**) had the molecular formula of C_21_H_21_NO_6_, as determined by its HRESIMS. The ^1^H and ^13^C NMR data of **10** (Table 3 and Table 4) were very similar to those of **8** and **9**. A detailed analysis of HSQC and HMBC spectra proved that **10** also contained a benzyl attached at position C-7 of isoquinoline unit. The HMBC correlations of H_2_-9 with C-6/C-7/C-8/C-10/C-11 (δ_C_ 170.0, C)/C-15 (δ_C_ 171.6, C) (Figure 4) suggested two hydroxyl groups attached at C-11 and C-15, respectively. In addition, the HMBC correlations from H_3_-19 (δ_H_ 1.92, 3H, s) to C-13/C-14/C-15 suggested a methyl attached at C-14. Furthermore, the HMBC correlations (Figure 4) from H_2_-17 (δ_H_ 2.58, 2H, q, *J* = 7.3 Hz) to C-16/C-18, from H_3_-18 (δ_H_ 1.02, 3H, t, *J* = 7.3 Hz) to C-16/C-17, and from H_3_-19 to C-16 suggested an 1-acetonyl group attached at C-13. Lastly, the chemical shift of C-12 (δ_C_ 163.1, C) indicated a hydroxy group attached at C-12. Therefore, the structure of **10** was established as shown.

Puniceusine K (**11**) was found to have the molecular formula C_22_H_21_NO_8_ by HRESIMS. The ^1^H and ^13^C NMR data of **11** (Table 3 and Table 4) were very similar to those of **10**. A detailed analysis of HSQC and HMBC spectra proved that **11** also contained a hexasubstituted benzyl attached at position C-7 of isoquinoline unit. The HMBC correlations of H_2_-9 with C-6/C-7/C-8/C-10/C-11 (δ_C_ 157.1, C)/C-15 (δ_C_ 153.0, C) suggested two hydroxyl groups attached at C-11 and C-15, respectively. Additionally, the HMBC correlations (Figure 4) from H-17 (δ_H_ 5.56, d, *J* = 2.5 Hz) to C-13/C-14/C-16/C-18/C-19, from H-18 (δ_H_ 4.36, qd, *J* = 6.5, 2.5 Hz) to C-19, and from H_3_-19 (δ_H_ 0.94, d, *J* = 6.5 Hz, 3H) to C-17/C-18, suggested a 5-hydroxy-2-hexene-4-lactone group attached on the benzene ring via C-13 and C-14. In addition, the HMBC correlation of δ_H_ 3.76 (s, 3H) with C-12 (δ_C_ 136.8, C) suggested a methoxy group attached at C-12 of the benzene ring. The assignment of the substituent groups at positions C-12, C-13 and C-14 of the benzene ring was further supported by comparison of the ^1^H and ^13^C NMR data of the same isobenzofuran moiety in **11**, embeurekol C [17] and acetophthalidin [18]. In addition, the small ^3^*J*_HH_ value (2.5 Hz) between H-17 and H-18 in **11** was closely similar to that of embeurekol C [17]. Furthermore, the specific rotation value of **11** ([α]${}_{D}^{25}$ −9.1 (*c* 0.1, CH_3_OH)) was close to that of embeurekol C ([α]${}_{D}^{20}$ −17 (*c* 0.05, CH_3_OH)) [17], and the experimental ECD spectrum of **11** (Figure S76) was greatly similar to that of embeurekol C [17]. These data suggested that the absolute configuration of **11** was also 17*R*, 18*S* for that of embeurekol C.

Puniceusine L (**12**) had a molecular formula of C_18_H_17_NO_5_ on the basis of its HRESIMS and NMR data. Its ^1^H and ^13^C NMR data (Table 3 and Table 4) showed a similarity to those of **8**–**11**. A detailed analysis of HSQC and HMBC spectra suggested that **12** contained the same isoquinoline unit as **8**–**11**. In addition, considering the molecular formula and unsaturation degrees of **12**, the HMBC correlations from H_2_-9 to C-6/C-7/C-8/C-10/C-11/C-14, from H-12 to C-10/C-11/C-13/C-15, from H_2_-15 to C-12/C-13/C-16, and from H_3_-16 to C-13/C-15 (Figure 4), suggested a 6-ethyl-4-hydroxy-2*H*-pyran-2-one unit attached at the methylene C-9 of isoquinoline unit. The above assignment was further confirmed by a single crystal X-ray diffraction analysis (Figure 3).

Puniceusine M (**13**) had the molecular formula of C_19_H_19_NO_5_ on the basis of its HRESIMS. The ^1^H and ^13^C NMR data (Table 3 and Table 4) were very similar to those of **12**. The only difference between them was the disappearance of one aromatic hydrogen and the additional presence of a methyl (δ_H_ 2.01 (3H, s), δ_C_ 9.9). The HMBC correlations from H_3_-17 (δ_H_ 2.01) to C-11/C-12/C-13 suggested the additional methyl attached at C-12. Therefore, the structure of **13** was established as shown.

Puniceusine N (**14**) had the molecular formula C_22_H_32_NO_4_^+^, as determined by HRESIMS. The ^1^H and ^13^C NMR (Table 3 and Table 4) data of **14** showed a similarity to those of **2**, and the clearest difference between them was the additional presence of two methyls, seven methylenes (one oxygenated), one methine, and one carboxyl in **14**. Detailed analysis of the HMBC and COSY spectra proved that **14** contained the same isoquinoline unit as that of **2**. In addition, combining with the COSY correlation of H_2_-10 (*δ*_H_ 4.88, t, *J* = 6.3 Hz) with H_2_-11 (*δ*_H_ 3.18, t, *J* = 6.3 Hz) (Figure 4), the HMBC correlations from H_2_-10 to C-1/C-3/C-11/C-12 (*δ*_C_ 171.8, C), and from H_2_-11 to C-9/C-12 (Figure 4), suggested a -CH_2_-CH_2_-COO- group attached at the nitrogen atom of isoquinoline unit. Furthermore, the sequential COSY correlations of H_2_-13/H-14/H_2_-15/H_2_-16/H_2_-17/H_2_-18, and H-14/H_2_-19/H_3_-20 (Figure 4), together with the HMBC correlations from H_2_-13 to C-12/C-14/C-15, from H_2_-15 to C-13/C-14/C-16, from H_2_-17 to C-16/C-18, from H_3_-18 to C-16/C-17, from H_2_-19 to C-13/C-14/C-15/C-20, and from H_3_-20 to C-14/C-19 (Figure 4), suggested that the 2-ethylhexanol group connected with the carboxyl of the -CH_2_-CH_2_-COO- group to form an ester. The optical rotation and measured CD data of **14** were zero, which indicated **14** was a racemic mixture. However, an HPLC analysis of **14** with a chiral column (CHIRALPAK IA and IB, respectively, 4.6 mm × 250 mm column), eluting with n-hexane/ethanol/TFA (Figures S99 and S100), showed a big trailing peak. The reason for this could be that the two kinds of chiral columns were not suitable for the chiral separation of **14**. Thus, the structure of **14** was determined as shown.

All of the 14 compounds were evaluated for their enzyme inhibitory activity against five PTPs including CD45, SHP1, TCPTP, PTP1B and LAR, cytotoxicity towards human lung adenocarcinoma cell line H1975, and antibacterial activity. The results of protein phosphatase inhibition assays (Table 5) showed that only **3** and **4** selectively exhibited significant inhibitory activity against CD45 with IC_50_ values of 8.4 and 5.6 µM, respectively, and **1**, **8**, **9**, **10**, **12** and **13** showed a mild inhibitory activity against several PTPs. A cytotoxicity assay (Table 5) showed that only **4** had a moderate cytotoxicity towards H1975 cell lines with an IC_50_ value of 11.0 µM. The analysis of the relationship of their structures, enzyme inhibitory activity and cytotoxicity displayed that the substituents at C-7 of the isoquinoline nucleus could greatly affect their bioactivity. In addition, antibacterial assays exhibited that **14** had medium antibacterial activity towards *Staphylococcus aureus*, methicillin-resistant *S. aureus* (MRSA), and *Escherichia coli,* with MIC values of 100 µg/mL, and **4** could inhibit the growth of *E. coli* with a MIC value of 100 µg/mL, while other compounds did not show clear antibacterial activity towards the three indicators. The results indicated that -C=N^+^ unit was an active center for the antibacterial activity of **14**.

## 3. Experimental Section

### 3.1. General Experimental Procedure

The procedures were the same as previously reported [13,14].

### 3.2. Fungal Material

The strain *Aspergillus puniceus* SCSIO z021 was isolated from a deep-sea sediment of Okinawa Trough (27°34.01′ N and 126°55.59′ E, ~1589 depth), which was located approximately 4.7 km from an active hydrothermal vent. The strain (GenBank accession number KX258801) was identified as *Aspergillus puniceus* through DNA extraction, ITS sequence amplification and sequence alignment, which has a 99% similarity to *A. puniceus* (GenBank accession number GU456970). The strain *A. puniceus* SCSIO z021 was deposited in the RNAM Center, South China Sea Institute of Oceanology, Chinese Academy of Science.

### 3.3. Fermentation and Extraction

The fungus strain was cultivated on potato glucose agar (PDA) plate containing 3% sea salt at 28 °C for 7 days. The spores were selected and transferred to a complex culture medium (glucose 1%, D-mannitol 2%, maltose 2%, corn meal 0.05%, monosodium glutamate 1%, KH_2_PO_4_ 0.05%, MgSO_4_·7H_2_O 0.03%, yeast extract 0.3%, sea salt 3%) to obtain a spore suspension that was cultured in a shaker at 28 °C for 3 days at a rotating speed of 180 rmp. The fungus was cultured in 1 L Erlenmeyer flasks each containing 300 mL of 3# medium (glucose 1%, D-mannitol 2%, maltose 2%, corn meal 0.05%, monosodium glutamate 1%, KH_2_PO_4_ 0.05%, MgSO_4_·7H_2_O 0.03%, yeast extract 0.3%, sea salt 3%) at 28 °C for 33 days under static condition. After fermentation, the broth and mycelia were separated with gauze. The broth was extracted with XAD-16 resin and sequentially eluted with H_2_O and EtOH to obtain crude extract (61.7 g). The mycelia was extracted three times with acetone, and further extracted three times with EtOAc to yield a crude extract (48.2 g).

### 3.4. Isolation and Purification

The combined extracts (109.9 g) were subjected to a normal-phase silica gel column eluting with a gradient of dichloromethane (DCM)/MeOH (100:0, 98:2, 95:5, 95:5, 90:10, 80:20, 70:30, 50:50, 0:100) to give nine subfractions (Fr.1–Fr.9) based on TLC analysis. Fr.4 (6.6 g) was separated by ODS column using MeOH-H_2_O-TFA (5:95:0.02 to 100:0:0.02) as eluent to afford 10 subfractions (Fr.4.1–Fr.4.10). Fr.4.1 was separated by Sephadex LH-20 eluting with MeOH followed by semipreparative HPLC (MeOH/H_2_O/TFA, 28:72:0.03, 3 mL/min) to yield **1** (11.6 mg, *t*_R_ = 15.8 min). Fr.4.3 was subjected to Sephadex LH-20 using MeOH as mobile phase, which was further purified by semipreparative HPLC (CH_3_CN/H_2_O/TFA, 17:83:0.03, 3 mL/min) to yield **2** (30.7 mg, *t*_R_ = 16.3 min). Fr.4.5 was isolated by Sephadex LH-20 eluting with MeOH, then purified by semipreparative HPLC (CH_3_CN/H_2_O/TFA, 25:75:0.03, 3 mL/min) to yield **7** (2.9 mg, *t*_R_ = 25.9 min). Fr.4.6 was separated by Sephadex LH-20 with a mobile phase of MeOH, and then further purified by semipreparative HPLC (MeOH/H_2_O/TFA, 45:55:0.03, 3 mL/min) to yield **10** (2.8 mg, *t*_R_ = 16.1 min) and **12** (15.2 mg, *t*_R_ = 14.5 min). Fr.4.8 was isolated by silica gel column eluting with a gradient of CH_2_Cl_2_/MeOH (100:0, 80:1, 60:1, 40:1, 10:1, 5:1, 1:1, 0:100) to obtain three fractions, then Fr.4.8.1 was further purified by semipreparative HPLC (CH_3_CN/H_2_O/TFA, 49:51:0.03, 3 mL/min) to yield **14** (29.4 mg, *t*_R_ = 20.2 min). Fr.6 (11.0 g) was separated by ODS column using MeOH-H_2_O-TFA (5:95:0.02 to 100:0:0.02) as mobile phase to yield eight subfractions (Fr.6.1–Fr.6.8). Fr.6.3 was purified by semipreparative HPLC (CH_3_CN/H_2_O/TFA, 21:79:0.03, 3 mL/min) to obtain **6** (4.0 mg, *t*_R_ = 19.5 min), **3** (17.6 mg, *t*_R_ = 18.4 min) and **4** (12.2 mg, *t*_R_ = 19.7 min). Fr.6.6 was isolated by semipreparative HPLC (CH_3_CN/H_2_O/TFA, 24:76:0.03, 3 mL/min) to yield **11** (7.8 mg, *t*_R_ = 16.0 min) and **5** (4.3 mg, *t*_R_ = 14.4 min). Fr.6.7 was isolated by Sephadex LH-20 with MeOH as mobile phase, then further purified by semipreparative HPLC (CH_3_CN/H_2_O/TFA, 28:72:0.03, 3 mL/min) to yield **8** (4.1 mg, *t*_R_ = 39.2 min) and **9** (2.8 mg, *t*_R_ = 18.4 min). Fr.6.8 was separated by Sephadex LH-20, eluting with MeOH and further purified by semipreparative HPLC (CH_3_CN/H_2_O/TFA, 35:65:0.03, 3 mL/min) to get **13** (10.3 mg, *t*_R_ = 18.0 min).

Puniceusine A (**1**): white acicular crystal; UV (CH_3_OH) *λ*_max_ (log *ε*) 204 (1.42), 208 (1.42), 241 (1.56), 258 (1.58), 357 (0.74) nm; ^1^H and ^13^C NMR, Table 1 and Table 2; HRESIMS *m*/*z* 176.0709 [M + H]^+^ (calcd for C_10_H_10_NO_2_, 176.0706).

Puniceusine B (**2**): pale yellow powder; UV (CH_3_OH) *λ*_max_ (log *ε*) 206 (1.61), 245 (1.75), 260 (1.83), 305 (0.73), 360 (0.84) nm; ^1^H and ^13^C NMR, Table 1 and Table 2; HRESIMS *m*/*z* 190.0864 [M + H]^+^ (calcd for C_11_H_12_NO_2_, 190.0863).

Puniceusine C (**3**): pale yellow powder; UV (CH_3_OH) *λ*_max_ (log *ε*) 205 (1.87), 244 (1.98), 262 (2.04), 332 (1.07) nm; IR (film) *ν*_max_ 3415, 1678, 1436, 1382, 1321, 1203, 1184, 1130, 1022, 954, 900, 840, 800, 723 cm^−1^; ^1^H and ^13^C NMR, Table 1 and Table 2; HRESIMS *m*/*z* 363.1339 [M + H]^+^ (calcd for C_21_H_19_N_2_O_4_, 363.1349).

Puniceusine D (**4**): pale yellow powder; UV (CH_3_OH) *λ*_max_ (log *ε*) 207 (1.86), 243 (1.88), 260 (1.88) nm; IR (film) *ν*_max_ 3402, 1678, 1643, 1566, 1384, 1342, 1197, 1132, 1045, 989, 954, 842, 800, 723 cm^−1^; ^1^H and ^13^C NMR, Table 1 and Table 2; HRESIMS *m*/*z* 363.1339 [M + H]^+^ (calcd for C_21_H_19_N_2_O_4_, 363.1338).

Puniceusine E (**5**): pale yellow powder; UV (CH_3_OH) *λ*_max_ (log *ε*) 206 (2.07), 248 (1.83), 265 (2.00), 337 (1.06) nm; IR (film) *ν*_max_ 1703, 1678, 1365, 1178, 0012, 835, 800, 721 cm^−1^; ^1^H and ^13^C NMR, Table 1 and Table 2; HRESIMS *m*/*z* 377.1496 [M + H]^+^ (calcd for C_22_H_21_N_2_O_4_, 377.1486).

Puniceusine F (**6**): colorless crystals; [α]${}_{D}^{25}$ 0 (*c* 0.10, CH_3_OH); UV (CH_3_OH) *λ*_max_ (log *ε*) 206 (1.63), 244 (1.76), 261 (1.71) nm; IR (film) *ν*_max_ 3419, 1703, 1681, 1363, 1201, 1180, 1134, 837, 800, 721 cm^−1^; ^1^H and ^13^C NMR, Table 1 and Table 2; HRESIMS *m*/*z* 262.1076 [M + H]^+^ (calcd for C_14_H_16_NO_4_, 262.1074).

Puniceusine G (**7**): yellow powder; UV (CH_3_OH) *λ*_max_ (log *ε*) 200 (1.26), 246 (1.46) nm; IR (film) *ν*_max_ 3412, 1680, 1440, 1195, 1136, 1028, 844, 800, 725 cm^−1^; ^1^H and ^13^C NMR, Table 1 and Table 2; HRESIMS *m*/*z* 254.1182 [M + H]^+^ (calcd for C_16_H_16_NO_2_, 254.1176).

Puniceusine H (**8**): pale yellow powder; UV (CH_3_OH) *λ*_max_ (log *ε*) 191 (1.61), 204 (2.08), 242 (1.06), 272 (1.12) nm; IR (film) *ν*_max_ 3367, 1680, 1456, 1417, 1394, 1201, 1139, 1020, 839, 802, 721 cm^−1^; ^1^H and ^13^C NMR, Table 2 and Table 3; HRESIMS *m*/*z* 398.1244 [M + H]^+^ (calcd for C_21_H_20_NO_7_, 398.1234).

Puniceusine I (**9**): yellow powder; [α]${}_{D}^{25}$ + 60.7 (*c* 0.10, CH_3_OH); UV (CH_3_OH) *λ*_max_ (log *ε*) 205 (1.30), 237 (0.90), 244 (0.92), 276 (0.63) nm; ECD (CH_3_OH) *λ*_max_ (Δ*ε*) 201 (+4.35), 202 (+4.71), 214 (−4.54), 232 (−1.32), 246 (−2.71), 279 (+0.44), 322 (−1.45) nm; IR (film) *ν*_max_ 3402, 1699, 1681, 1361, 1201, 1136, 837, 800, 721 cm^−1^; ^1^H and ^13^C NMR, Table 3 and Table 4; HRESIMS *m*/*z* 354.1331 [M + H]^+^ (calcd for C_20_H_20_NO_5_, 354.1336).

Puniceusine J (**10**): pale yellow powder; UV (CH_3_OH) *λ*_max_ (log *ε*) 195 (2.08), 208 (1.06), 243 (1.12) nm; IR (film) *ν*_max_ 3406, 1678, 1392, 1321, 1203, 1132, 840, 800, 723 cm^−1^; ^1^H and ^13^C NMR, Table 3 and Table 4; HRESIMS *m*/*z* 384.1455 [M + H]^+^ (calcd for C_21_H_22_NO_6_, 384.1442).

Puniceusine K (**11**): pale yellow powder; [α]${}_{D}^{25}$ − 155.6 (*c* 0.10, CH_3_OH); UV (CH_3_OH) *λ*_max_ (log *ε*) 205 (1.30), 237 (0.90), 244 (0.92), 276 (0.63) nm; ECD (CH_3_OH) *λ*_max_ (Δ*ε*) 215 (−2.39), 229 (+0.45), 238 (+0.29), 255 (+0.67), 304 (−0.18) nm; IR (film) *ν*_max_ 3390, 1674, 1435, 1371, 1319, 1199, 1134, 840, 800, 723 cm^−1^; ^1^H and ^13^C NMR, Table 3 and Table 4; HRESIMS *m*/*z* 428.1348 [M + H]^+^ (calcd for C_22_H_22_NO_8_, 428.1340).

Puniceusine L (**12**): colorless crystals; UV (CH_3_OH) *λ*_max_ (log *ε*) 206 (2.14), 264 (2.04), 366 (1.12) nm; IR (film) *ν*_max_ 3080, 1678, 1643, 1396, 1319, 1197, 1130, 839, 798, 721 cm^−1^; ^1^H and ^13^C NMR, Table 3 and Table 4; HRESIMS *m*/*z* 328.1195 [M + H]^+^ (calcd for C_18_H_18_NO_5_, 328.1179).

Puniceusine M (**13**): pale yellow powder; UV (CH_3_OH) *λ*_max_ (log *ε*) 207 (2.08), 244 (1.74), 267 (1.84) nm; IR (film) *ν*_max_ 3404, 1678, 1394, 1319, 1201, 1176, 1138, 1026, 837, 800, 721 cm^−1^; ^1^H and ^13^C NMR, Table 3 and Table 4; HRESIMS *m*/*z* 342.1332 [M + H]^+^ (calcd for C_19_H_20_NO_5_, 342.1336).

Puniceusine N (**14**): pale yellow powder; [α]${}_{D}^{25}$ 0 (*c* 0.10, CH_3_OH); UV (CH_3_OH) *λ*_max_ (log *ε*) 206 (1.89), 232 (1.61), 266 (2.08), 309 (1.06), 364 (1.12) nm; IR (film) *ν*_max_ 3423, 1680, 1363, 1195, 1182, 1128, 839, 800, 719 cm^−1^; ^1^H and ^13^C NMR, Table 3 and Table 4; HRESIMS *m*/*z* 374.2329 [M]^+^ (calcd for C_22_H_32_NO_4_, 374.2326).

### 3.5. X-ray Crystallographic Analysis of ***6*** and ***12***

The crystal data were obtained on a Rigaku MicroMax 007 diffractometer (Rigaku Corporation, Tokyo, Japan)with Cu Kα radiation and a graphite monochromator. The crystal structures of **6** and **12** were solved by direct methods with the SHELXTL and refined by full-matrix, least-squares techniques. Crystallographic data for **6** and **12** were deposited with the Cambridge Crystallographic Data Centre as supplementary publication numbers, CCDC 2112471 and 2112479, respectively.

Crystal data for **6**: C_14_H_14.0375_NO_4_, *FW* = 260.30; colorless crystal from MeOH; crystal size = 0.15 × 0.12 × 0.1 mm^3^; *T* = 100.00 (10) K; monoclinic, space group P2_1_/c (no. 14); unit cell parameters: *a* = 4.82780 (10) Å, *b* = 13.4963 (3) Å, *c* = 22.6665 (7) Å, *α* = 90°, *β* = 96.025 (3)°, *γ* = 90°, *V* = 1468.73 (6) Å^3^, *Z* = 4, *D*_calc_ = 1.177 g/cm^3^, *F* (000) = 548.0, *μ* (CuK*α*) = 0.724 mm^−1^; 7185 reflections measured (7.634° ≤ 2θ ≤ 147.79°), 2852 unique (*R*_int_ = 0.0197, R_sigma_ = 0.0255), which were used in all calculations. The final R_1_ was 0.0487 (I > 2σ(I)) and *w*R_2_ was 0.1406 (all data).

Crystal data for **12**: C_18_H_17_NO_5_, *FW* = 327.32; colorless crystal from MeOH; crystal size = 0.13 × 0.12 × 0.1 mm^3^; *T* = 100.00 (10) K; triclinic, space group P-1 (no. 2); unit cell parameters: *a* = 7.6037 (4) Å, *b* = 9.9253 (5) Å, *c* = 10.5261 (6) Å, *α* = 70.543 (5)°, *β* = 85.628 (4)°, *γ* = 81.605 (4)°, *V* = 740.68(7) Å^3^, *Z* = 2, *D*_calc_ = 1.468 g/cm^3^, *F* (000) = 344.0, *μ* (CuK*α*) = 0.897 mm^−1^; 7019 reflections measured (8.914° ≤ 2θ ≤ 148.202°), 2880 unique (*R*_int_ = 0.0259, R_sigma_ = 0.0317) which were used in all calculations. The final R_1_ was 0.0419 (I > 2σ(I)) and *w*R_2_ was 0.1150 (all data).

### 3.6. ECD Calculations

The ECD calculation for **9** was performed by Gaussian 16 program package. The procedures were the same as described in our previous study [13,14]. Briefly, the conformational search was performed by a MMFF model, then the conformers with lower relative energies (<10 kcal/mol) were subjected to geometry optimization with the DFT method at the B3LYP/6-311G(d) level. Vibrational frequency calculations were carried out at the same level to evaluate their relative thermal (ΔE) and free energies (ΔG) at 298.15 K. The geometry-optimized conformers were further calculated at the M06-2X/def2-TZVP level and the solvent (methanol) effects were taken into consideration by using SMD. The optimized conformers with a Boltzmann distribution of more than 1% population were subjected to ECD calculation, which were performed by TDDFT methodology at the PBE1PBE/TZVP level. The ECD spectrum was generated by the software SpecDis using a Gaussian band shape with 0.3 eV exponential half-width from dipole-length dipolar and ratational strengths. The calculated spectrum of **9** was generated from the low-energy conformers according to the Boltzmann distribution of each conformer in MeOH solution. Details regarding optimized conformation geometries, thermodynamic parameters, and Boltzmann distributions (Tables S1–S3) of all conformations are provided in the Supporting Information.

### 3.7. Protein Phosphatase Inhibition Assays

The same methods as described in our previous study [13,14] were applied to test the inhibition activity of compounds **1**–**14** against five human protein tyrosine phosphatases (CD45, SHP1, TCPTP, PTP1B and LAR).

### 3.8. Cytotoxicity

Cytotoxic activity was evaluated using human lung adenocarcinoma cell line H1975 by CCK-8 method. Briefly, each of the test compounds was dissolved in DMSO and further diluted to give final concentrations of 80, 40, 20, 10, 5, 2.5, and 1.25 µg/mL, respectively. H1975 cells (5 × 10^3^ cells/plate) were seeded in 96-well plates and treated with compounds at the indicated concentration for 24 h, and then 10 μL CCK-8 reagent was added to each well, and the plates were incubated at 37 °C for another 4 h. Next, the optical density was measured at a wavelength of 450 nm with the Bio-Rad (Hercules, CA, USA) microplate reader. Dose–response curves were generated, and the IC_50_ values were calculated from the linear portion of log dose–response curves.

### 3.9. Antibacterial Assays

Antibacterial activities of **1**–**14** against *E. coli*, *S. aureus* and MRSA were evaluated using the 2-fold dilution assay in 96-microwell plates. Briefly, all the indicator bacteria were cultured on Luria−Bertani (LB) agar plates at 37 °C for 12 h, and then a single colony was picked into LB liquid medium and cultivated on a rotary shaker at 37 °C for 12 h. Then, the bacterial suspensions with LB medium were diluted until the difference of the OD_600_ values between the bacterial suspensions and the medium was 0.01~0.02. Each of the tested compounds was dissolved in DMSO to give an initial concentration of 5 mg/mL, and further diluted with the bacterial suspensions by twofold serial dilution to give a final concentration of 100, 50, 25, 12.5, 6.25, and 3.125 μg/mL, respectively. The 96-well plates were incubated at 37 °C for 12 h. MIC value was determined as the lowest concentration with no visible bacterial growth. Ampicillin was used as the positive control and DMSO as the negative control. All experiments were performed three times.

## 4. Conclusions

Summarily, 14 new isoquinoline alkaloids (**1**–**14**) were obtained from the deep-sea-derived fungus, *A. puniceus* SCSIO z021. Compounds **3**–**5** and **8**–**13** unprecedentedly contained an isoquinolinyl, a polysubstituted benzyl or a pyronyl at position C-7 of the isoquinoline nucleus, which was different from 1-benzylisoquinoline analogues and other isoquinoline alkaloids from plants commonly containing substituents at positions C-1, N-2, C-3, C-4 or C-8 of isoquinoline skeleton. In addition, **3** and **4** showed selective inhibitory activity against CD45; **4** also had moderate cytotoxicity towards human lung adenocarcinoma cell line H1975, and **14** contained an active center -C=N^+^ which had evident antibacterial activity towards three indicator bacteria. An analysis of the relationship of the structures, enzyme inhibitory activity and cytotoxicity of **1**–**14** displayed that the substituents at C-7 of the isoquinoline nucleus could greatly affect their bioactivity. The results greatly enrich the structural diversity of isoquinoline alkaloids from fungi, and provide a potential lead compound for the development of a selective CD45 inhibitor and anticancer drug.

## Figures and Tables

**Table 1:** ^1^H NMR Data for Compounds **1**–**7** (*δ* in ppm, *J* in Hz) in DMSO-*d*_6_ or Methanol-*d*_4_.

No.	1 ^*a*,*c*^	2 ^*a*,*d*^	3 *^b^*^,*d*^	4 ^*a*,*d*^	5 ^*a*,*d*^	6 *^a^*^,*d*^	7 ^*b*,*d*^
1	9.45, s	9.41, s	9.34, s	9.59, s	9.58, s	9.49, s	9.87, s
3	8.40, d (5.7)	8.24 d (6.5)	8.08, d (6.3)	8.29, d (7.0)	8.30, d (6.6)	8.29, d (6.0)	8.47, d (6.3)
4	8.06, d (6.4)	8.04 d (6.5)	7.90, d (6.3)	8.05, d (6.5)	8.09, d (6.6)	8.10, d (6.0)	8.22, d (6.3)
5	7.13, s	7.13 s	6.95, s	7.11, s	7.18, s	7.23, s	7.31, s
7	6.81, s						
9		2.25, s	4.32, s	4.47, s	4.61, s	2.97, dd (14.0, 5.5) 3.17, dd (14.0, 8.5)	
10						2.85, m	
11				8.58, d (7.0)	8.67, d (6.8)		
12				8.31, d (7.8)	8.39, d (6.8)	1.27, d (7.5)	8.24, s
13							2.93, s
14				9.52, s	9.68, s		2.51, s
16				7.00, s			
18					2.38, s		
6-OCH_3_	3.96, s	4.07, s	4.09, s	3.79, s	3.84, s	4.10, s	4.24, s
6′-OCH_3_			4.09, s				
17-OCH_3_				3.98, s	3.79, s		

*^a^* 500 MHz for ^1^H NMR; *^b^* 700 MHz for ^1^H NMR; *^c^* DMSO-*d*_6_; *^d^* Methanol-*d*_4_.

**Table 2:** ^13^C NMR data for Compounds **1**–**8** (*δ* in ppm) in DMSO-*d*_6_ or Methanol-*d*_4_.

No.	1 ^*a*,*c*^	2 ^*a*,*d*^	3 *^b^*^,*d*^	4 ^*a*,*d*^	5 ^*a*,*d*^	6 *^a^*^,*d*^	7 ^*b*,*d*^	8 ^*a*,*c*^
1	141.8, CH	141.5, CH	141.9, CH	142.4, CH	142.3, CH	142.3, CH	132.7, CH	141.2, CH
3	133.2, CH	131.4, CH	130.8, CH	132.1, CH	132.0, CH	132.2, CH	123.5, CH	131.5, CH
4	122.3, CH	123.7, CH	123.1, CH	123.7, CH	123.9, CH	123.8, CH	114.7, CH	122.0, CH
4a	140.9, C	140.9, C	141.2, C	141.5, C	141.5, C	141.6, C	137.2, C	139.1, C
5	97.8, CH	98.6, CH	96.1, C	98.8, CH	99.4, CH	99.0, CH	90.3, CH	97.2, CH
6	166.9, C	168.4, C	169.1, C	168.5, C	168.2, C	168.3, C	155.0, C	165.8, C
7	103.5, CH	116.8, C	118.9, C	118.5, C	120.8, C	119.1, C	113.3, C	117.3, C
8	158.6, C	155.7, C	163.9, C	156.3, C	156.2, C	156.4, C	121.2, C	155.8, C
8a	115.5, C	117.5, C	118.7, C	117.8, C	117.9, C	117.9, C	114.0, C	115.8, C
9		9.2, CH_3_	19.4, CH_2_	20.7, CH_2_	21.3, CH_2_	28.0, CH_2_	112.0, C	19.3, CH_2_
10				113.6, C	120.0, C	40.4, CH	148.7, C	115.0, C
10a				139.7, C	138.9, C			
11				121.6, CH	122.7, CH	181.7, C	120.7, C	151.3, C
12				131.2, CH	131.0, CH	18.1, CH_3_	115.0, C	107.1, CH
13							8.0, CH_3_	139.5, C
14				143.3, CH	143.4, CH		8.4, CH_3_	148.1, C
14a				115.9, C	119.0, C			
15				161.0, C	156.7, C			130.2, C
16				100.1, CH	120.6, C			195.1, C
17				167.5, C	167.7, C			196.9, C
18					10.8, CH_3_			23.7, CH_3_
6-OCH_3_	56.4, CH_3_	57.4, CH_3_	57.0, CH_3_	56.9, CH_3_	57.0, CH_3_	57.4, CH_3_	47.9, CH_3_	56.4, CH_3_
14-OCH_3_								59.7, CH_3_
17-OCH_3_				57.1, CH_3_	62.1, CH_3_			

*^a^* 125 MHz for ^13^C NMR; *^b^* 175 MHz for ^13^C NMR; *^c^* DMSO-*d*_6_; *^d^* Methanol-*d*_4_.

**Table 3:** ^1^H NMR data for Compounds **8**–**14** (*δ* in ppm, *J* in Hz) in DMSO-*d*_6_ or Methanol-*d*_4_.

No.	8 ^*a*,*c*^	9 *^b^*^,*c*^	10 ^*b*,*d*^	11 ^*a*,*d*^	12 *^a^*^,*d*^	13 *^a^*^,*d*^	14 ^*a*,*d*^
1	9.49, s	9.51, s	9.47, s	9.49, s	9.48, s	9.52, s	9.64, s
3	8.36, d (6.6)	8.39, d (6.6)	8.23, d (6.6)	8.25, d (6.4)	8.26, d (5.5)	8.29, d (6.6)	8.37, d (6.9)
4	8.08, d (6.6)	8.11, d (6.6)	8.05, d (6.6)	8.06, d (6.4)	8.06, d (5.5)	8.10, d (6.6)	8.08, d (6.9)
5	7.11, s	7.26, s	7.17, s	7.16, s	7.16, s	7.23, s	7.19, s
9	4.01, s	3.84, d (14.9)4.03, d (14.8)	3.96, s	4.21, s	3.92, s	3.97, s	2.29, s
10							4.88, t (6.3)
11							3.18, t (6.3)
12	6.58, s	6.27, s			6.12, s		
13							4.00, d (5.7)
14							1.46, m
15					2.52, q (7.5)	2.60, q (7.5)	1.16, m
16		5.22, m			1.19, t (7.5)	1.17, t (7.5)	1.17, m
17		4.69, d (11.5) 4.84, dd (11.5, 2.5)	2.58, q (7.3)	5.56, d (2.5)		2.01, s	1.18, m
18	2.30, s	1.35, d (6.2)	1.02, t (7.3)	4.36, qd (6.5, 2.5)			0.79, t (7.5)
19	3.52, s		1.92, s	0.94, d (6.5)			1.24, m
20							0.82, t (6.5)
6-OCH_3_	3.77, s	4.00, s	4.11, s	4.03, s	4.07, s	4.13, s	
12-OCH_3_				3.76, s			

*^a^* 500 MHz for ^1^H NMR; *^b^* 700 MHz for ^1^H NMR; *^c^* DMSO-*d*_6_; *^d^* Methanol-*d*_4_.

**Table 4:** ^13^C NMR data for Compounds **9**–**14** (*δ* in ppm) in DMSO-*d*_6_ or Methanol-*d*_4_.

No.	9 *^b^*^,*c*^	10 ^*b*,*d*^	11 *^a^*^,*d*^	12 *^a^*^,*d*^	13 ^*a*,*d*^	14 *^a^*^,*d*^
1	141.2, CH	142.3, CH	142.0, C	142.2, CH	142.4, CH	145.4, CH
3	131.5, CH	131.7, CH	131.6, CH	131.8, CH	132.0, CH	134.9, CH
4	122.2, CH	123.6, CH	123.6, CH	123.6, CH	123.9, CH	124.4, CH
4a	139.0, C	141.5, C	141.4, C	141.5, C	141.7, C	139.7, C
5	97.7, CH	98.4, C	98.7, CH	98.8, C	98.9, CH	98.6, CH
6	165.6, C	168.1, C	168.7, C	168.3, C	167.7, C	168.8, C
7	117.7, C	119.2, C	118.7, C	119.1, C	118.8, C	117.1, C
8	154.8, C	158.7, C	156.7, C	157.4, C	157.5, C	155.8, C
8a	115.5, C	118.4, C	117.6, C	118.0, C	118.3, C	117.7, C
9	19.8, CH_2_	19.4, CH_2_	18.9, CH_2_	18.7, CH_2_	19.3, CH_2_	9.23, CH_3_
10	109.4, C	101.5, C	116.2, C	101.0, C	100.9, C	57.1, CH_2_
11	154.8, C	170.0, C	157.1, C	170.2, C	169.7, C	35.7, CH_2_
12	101.4, CH	163.1, C	136.8, C	101.2, CH	109.8, C	171.8, C
13	150.1, C	113.8, C	137.4, C	167.4, C	162.7, C	68.4, CH_2_
14	115.9, C	154.4, C	104.2, C	171.2, C	170.7, C	40.0, CH
15	145.3, C	171.6, C	153.0, C	27.4, CH_2_	25.1, CH_2_	31.3, CH_2_
16	79.3, CH	207.3, C	172.7, C	11.2, CH_3_	11.8, CH_3_	29.9, CH_2_
17	68.9, CH_2_	36.6, CH_2_	85.2, CH		9.9, CH_3_	23.9, CH_2_
18	21.3, CH_3_	7.8, CH_3_	68.6, CH			14.3, CH_3_
19		10.4, CH_3_	15.8, CH_3_			24.6, CH_2_
20						11.2, CH_3_
6-OCH_3_	56.7, CH_3_	57.3, CH_3_	57.2, CH_3_	57.2, CH_3_	57.5, CH_3_	57.5, CH_3_
12-OCH_3_			61.1, CH_3_			

*^a^* 125 MHz for ^13^C NMR; *^b^* 175 MHz for ^13^C NMR; *^c^* DMSO-*d*_6_; *^d^* Methanol-*d*_4_.

**Table 5:** Inhibition activity against five phosphatases and cytotoxicity of **1**–**14**.

Comp.	Inhibitory Effect against Phosphatases (IC_50_ in µM)					Cytotoxicity (IC_50_ in μM)
	CD45	SHP1	TCPTP	PTP1B	LAR	H1975 Cell (after 24 h)
**1**	29.7	38.3	196.9	182.2	100	76.2
**2**	>200	>200	>200	>200	>200	>80
**3**	8.4	20.7	>200	64.5	21.9	71.3
**4**	5.6	130.2	>200	150	26.1	11.0
**5**	102.6	145.1	>200	200	>200	>80
**6**	>200	200	>200	>200	>200	>80
**7**	>200	>200	>200	>200	>200	>80
**8**	49.0	100	>200	180	>200	>80
**9**	27.2	41.8	57.7	68.8	>200	>80
**10**	30.8	>200	>200	>200	100	>80
**11**	100	145.4	>200	190	>200	>80
**12**	38.5	116.8	>200	170	150	>80
**13**	11.1	40.26	>200	37.9	32.6	>80
**14**	200	>200	>200	>200	>200	>80
Na_3_VO_4_	-	4.4	2.4	1.6	-	-
AACQ	0.29	-	-	-	-	-

AACQ: (2-[(4-acetylphenyl)amino]-3-chloronaphthoquinone); “-”: Not tested.

## Supplementary Materials

The following supporting information can be downloaded at: www.mdpi.com/article/10.3390/md20010078/s1, Figures S1–S126: the 1D NMR, 2D NMR, HRESIMS, IR, and UV spectra of compounds **1**–**14**, Tables S1–S3: details of ECD calculation of compound **9**, Tables S4 and S5: X-ray crystallographic data for compound **6** and X-ray crystallographic data for compound **12**.
