# Supplementary material for: Isoquinoline Alkaloids as Protein Tyrosine Phosphatase Inhibitors from a Deep-Sea-Derived Fungus Aspergillus puniceus
Source: Mar Drugs. 2022 Jan 17;20(1):78. doi: 10.3390/md20010078 (PMC8781450; doi:10.3390/md20010078)

Supplementary Information

Isoquinoline alkaloids as protein tyrosine inhibitors from a deep-sea-derived fungus *Aspergillus puniceus*

Cheng-Mei Liu,[a,b] Fei-Hua Yao,[a,b] Xin-Hua Lu,[d] Xue-Xia Zhang,[d] Lian-Xiang Luo,[e] Xiao Liang,[a] Shu-Hua Qi[a, c, *]

[a] CAS Key Laboratory of Tropical Marine Bio-resources and Ecology/Guangdong Key Laboratory of Marine Materia Medica/Innovation Academy of South China Sea Ecology and Environmental Engineering, South China Sea Institute of Oceanology, Chinese Academy of Sciences, Guangzhou 510301, Guangdong, China

[b] University of Chinese Academy of Sciences, Beijing, 100049, China

[c] Southern Marine Science and Engineering Guangdong Laboratory (Guangzhou), 1119 Haibin Road, Nansha District, Guangzhou 511458, China

[d] New Drug Research & Development Co., Ltd, North China Pharmaceutical Group Corporation, Shijiazhuang, Hebei 050015, China

[e] The Marine Biomedical Research Institute, Guangdong Medical University, Zhanjiang, Guangdong, 524023, China

Contents

Figure S1. ^{1}H NMR spectrum of **1** in DMSO-d_6.
Figure S2. ^{13}C NMR spectrum of **1** in DMSO-d_6.
Figure S3. HMBC spectrum of **1** in DMSO-d_6.
Figure S4. Partial HMBC spectrum of **1** in DMSO-d_6.
Figure S5. HRESIMS spectrum of **1**.
Figure S6. UV spectrum of **1.**
Figure S7. ^{1}H NMR spectrum of **2** in methanol-d_4.
Figure S8. ^{13}C NMR spectrum of **2** in methanol-d_4.
Figure S9. HMBC spectrum of **2** in methanol-d_4.
Figure S10. Partial HMBC spectrum of **2** in methanol-d_4.

Figure S11. HRESIMS spectrum of **2**.
Figure S12. UV spectrum of **2.**
Figure S13. ^{1}H NMR spectrum of **3** in methanol-*d*4.
Figure S14. ^{13}C NMR spectrum of **3** in methanol-*d*4.
Figure S15. HSQC spectrum of **3** in methanol-*d*4.
Figure S16. HMBC spectrum of **3** in methanol-*d*4.
Figure S17. Partial HMBC spectrum of **3** in methanol-*d*4.
Figure S18. Partial HMBC spectrum of **3** in methanol-*d*4.
Figure S19. HRESIMS spectrum of **3**.
Figure S20. IR spectrum of **3.**
Figure S21. UV spectrum of **3.**
Figure S22. ^{1}H NMR spectrum of **4** in methanol-*d*4.
Figure S23. ^{13}C NMR spectrum of **4** in methanol-*d*4.
Figure S24. HSQC spectrum of **4** in methanol-*d*4.
Figure S25. Partial HSQC spectrum of **4** in methanol-*d*4.
Figure S26. HMBC spectrum of **4** in methanol-*d*4.
Figure S27. Partial HMBC spectrum of **4** in methanol-*d*4.
Figure S28. Partial HMBC spectrum of **4** in methanol-*d*4.
Figure S29. HRESIMS spectrum of **4**.
Figure S30. IR spectrum of **4.**
Figure S31. UV spectrum of **4.**
Figure S32. ^{1}H NMR spectrum of **5** in methanol-*d*4.
Figure S33. ^{13}C NMR spectrum of **5** in methanol-*d*4.
Figure S34. HSQC spectrum of **5** in methanol-*d*4.
Figure S35. HMBC spectrum of **5** in methanol-*d*4.
Figure S36. Partial HMBC spectrum of **5** in methanol-*d*4.
Figure S37. Partial HMBC spectrum of **5** in methanol-*d*4.
Figure S38. HRESIMS spectrum of **5**.
Figure S39. IR spectrum of **5.**
Figure S40. UV spectrum of **5.**
Figure S41. ^{1}H NMR spectrum of **6** in methanol-*d*4.
Figure S42. ^{13}C NMR spectrum of **6** in methanol-*d*4.
Figure S43. HSQC spectrum of **6** in methanol-*d*4.
Figure S44. HMBC spectrum of **6** in methanol-*d*4.
Figure S45. HRESIMS spectrum of **6**.
Figure S46. IR spectrum of **6.**
Figure S47. UV spectrum of **6.**
Figure S48. Chirality analysis of compound **6**.
Figure S49. ^{1}H NMR spectrum of **7** in methanol-*d*4.
Figure S50. ^{13}C NMR spectrum of **7** in methanol-*d*4.
Figure S51. HSQC spectrum of **7** in methanol-*d*4.
Figure S52. HMBC spectrum of **7** in methanol-*d*4.
Figure S53. Partial HMBC spectrum of **7** in methanol-*d*4.
Figure S54. Partial HMBC spectrum of **7** in methanol-*d*4.

Figure S55. HRESIMS spectrum of **7**.
Figure S56. IR spectrum of **7.**
Figure S57. UV spectrum of **7.**
Figure S58. ^{1}H NMR spectrum of **8** in DMSO-d_6.
Figure S59. ^{13}C and DEPT135 NMR spectra of **8** in DMSO-d_6.
Figure S60. HSQC spectrum of **8** in DMSO-d_6.
Figure S61. HMBC spectrum of **8** in DMSO-d_6.
Figure S62. Partial HMBC spectrum of **8** in DMSO-d_6.
Figure S63. Partial HMBC spectrum of **8** in DMSO-d_6.
Figure S64. Partial HMBC spectrum of **8** in DMSO-d_6.
Figure S65. HRESIMS spectrum of **8**.
Figure S66. IR spectrum of **8**.
Figure S67. UV spectrum of **8**.
Figure S68. ^{1}H NMR spectrum of **9** in DMSO-d_6.
Figure S69. ^{13}C and DEPT135 NMR spectra of **9** in DMSO-d_6.
Figure S70. HSQC spectrum of **9** in DMSO-d_6.
Figure S71. HMBC spectrum of **9** in DMSO-d_6.
Figure S72. Partial HMBC spectrum of **9** in DMSO-d_6.
Figure S73. Partial HMBC spectrum of **9** in DMSO-d_6.
Figure S74. HRESIMS spectrum of **9**.
Figure S75. IR spectrum of **9.**
Figure S76. UV spectrum of **9.**
Figure S77. CD spectrum of **9** in methanol.
Figure S78. ^{1}H NMR spectrum of **10** in methanol-d_4.
Figure S79. ^{13}C NMR spectrum of **10** in methanol-d_4.
Figure S80. HSQC spectrum of **10** in methanol-d_4.
Figure S81. HMBC spectrum of **10** in methanol-d_4.
Figure S82. Partial HMBC spectrum of **10** in methanol-d_4.
Figure S83. Partial HMBC spectrum of **10** in methanol-d_4.
Figure S84. HRESIMS spectrum of **10**.
Figure S85. IR spectrum of **10.**
Figure S86. UV spectrum of **10.**
Figure S87. ^{1}H NMR spectrum of **11** in methanol-d_4.
Figure S88. ^{13}C NMR spectrum of **11** in methanol-d_4.
Figure S89. HSQC spectrum of **11** in methanol-d_4.
Figure S90. HMBC spectrum of **11** in methanol-d_4.
Figure S91. Partial HMBC spectrum of **11** in methanol-d_4.
Figure S92. Partial HMBC spectrum of **11** in methanol-d_4.
Figure S93. HRESIMS spectrum of **11**.
Figure S94. IR spectrum of **11.**
Figure S95. UV spectrum of **11.**
Figure S96. CD spectrum of **11** in methanol.
Figure S97. ^{1}H NMR spectrum of **12** in methanol-d_4.
Figure S98. ^{13}C NMR spectrum of **12** in methanol-d_4.

Figure S99. HSQC spectrum of **12** in methanol-d_4.
Figure S100. HMBC spectrum of **12** in methanol-d_4.
Figure S101. Partial HMBC spectrum of **12** in methanol-d_4.
Figure S102. HRESIMS spectrum of **12.**
Figure S103. IR spectrum of **12.**
Figure S104. UV spectrum of **12.**
Figure S105. ^{1}H NMR spectrum of **13** in methanol-d_4.
Figure S106. ^{13}C NMR spectrum of **13** in methanol-d_4.
Figure S107. HSQC spectrum of **13** in DMSO-d_6.
Figure S108. HMBC spectrum of **13** in methanol-d_4.
Figure S109. Partial HMBC spectrum of **13** in methanol-d_4.
Figure S110. Partial HMBC spectrum of **13** in methanol-d_4.
Figure S111. HRESIMS spectrum of **13**.
Figure S112. IR spectrum of **13.**
Figure S113. UV spectrum of **13.**
Figure S114. ^{1}H NMR spectrum of **14** in methanol-d_4.
Figure S115. ^{13}C NMR spectrum of **14** in methanol-d_4.
Figure S116. HSQC spectrum of **14** in methanol-d_4.
Figure S117. HMBC spectrum of **14** in methanol-d_4.
Figure S118. Partial HMBC spectrum of **14** in methanol-d_4.
Figure S119. Partial HMBC spectrum of **14** in methanol-d_4.
Figure S120. Partial HMBC spectrum of **14** in methanol-d_4.
Figure S121. COSY spectrum of **14** in methanol-d_4.
Figure S122. HRESIMS spectrum of **14**.
Figure S123. IR spectrum of **14.**
Figure S124. UV spectrum of **14.**
Figure S125. Chirality analysis of compound **14** by CHIRALPAK IA
Figure S126. Chirality analysis of compound **14** by CHIRALPAK IB
Computational Methods
Table S1. Stable conformers of compound **9** used for ECD calculations.
Table S2. Important thermodynamic parameters (a.u.) of **9** at B3LYP/6-311G (d,p) level in the gas phase.
Table S3. Relative thermal energies (ΔE), relative free energies (ΔG), and equilibrium populations (P) of low-energy conformers of structures (16*R*)-**9** in methanol.
Table S4. Crystal data and structure refinement for compound **6**
Table S5. Crystal data and structure refinement for compound **12**

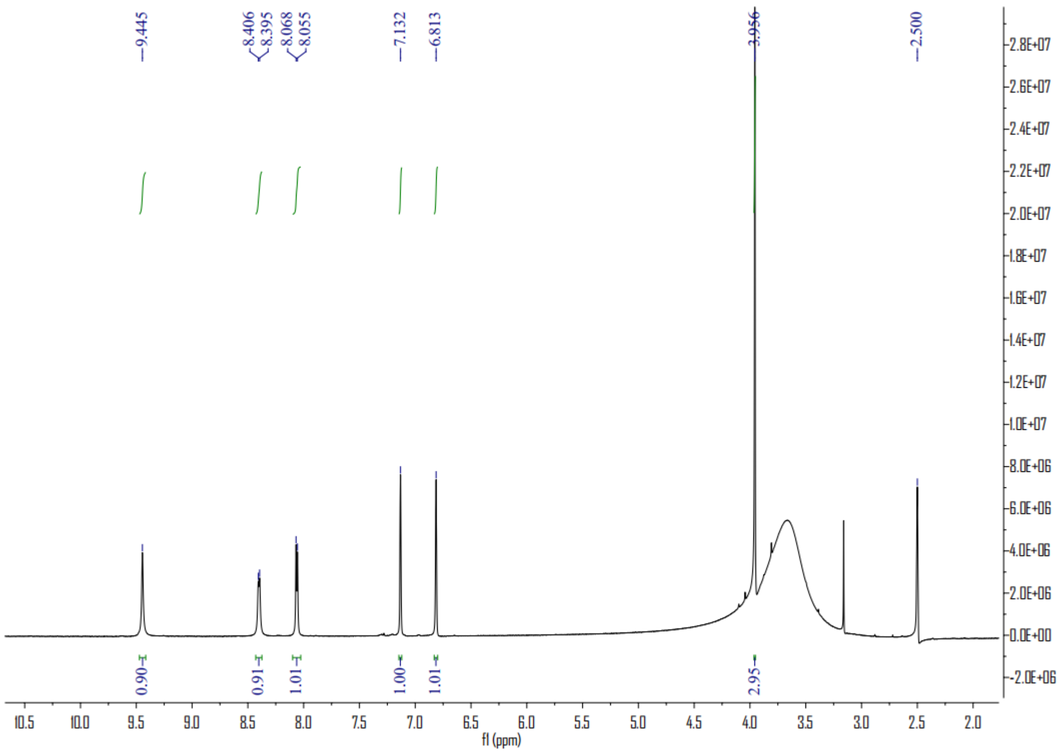

Figure S1. ^{1}H NMR spectrum of **1** in DMSO-d_6

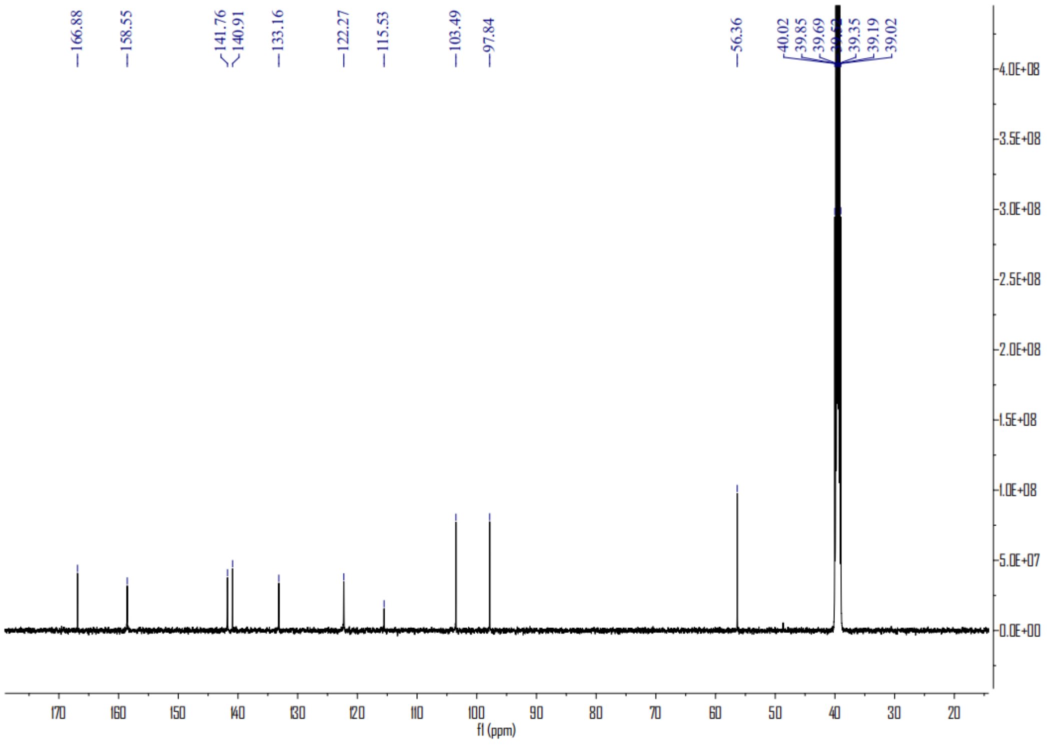

Figure S2. ^{13}C NMR spectrum of **1** in DMSO-d_6

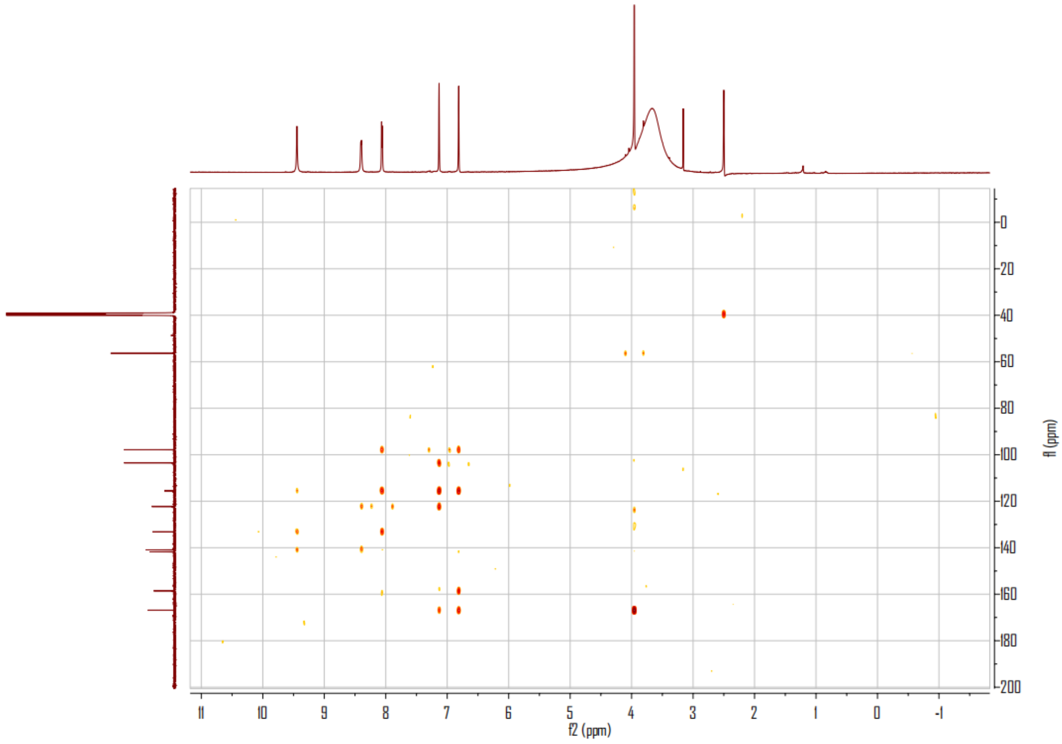

Figure S3. HMBC spectrum of **1** in DMSO-d_6

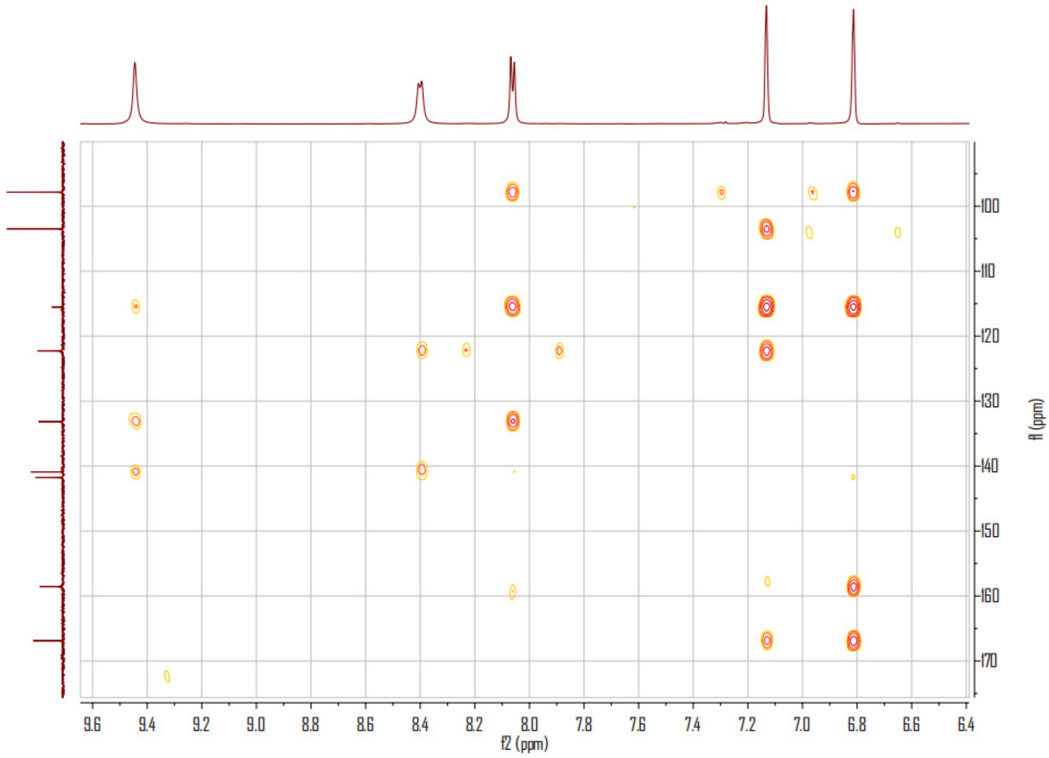

Figure S4. Partial HMBC spectrum of **1** in DMSO-d_6

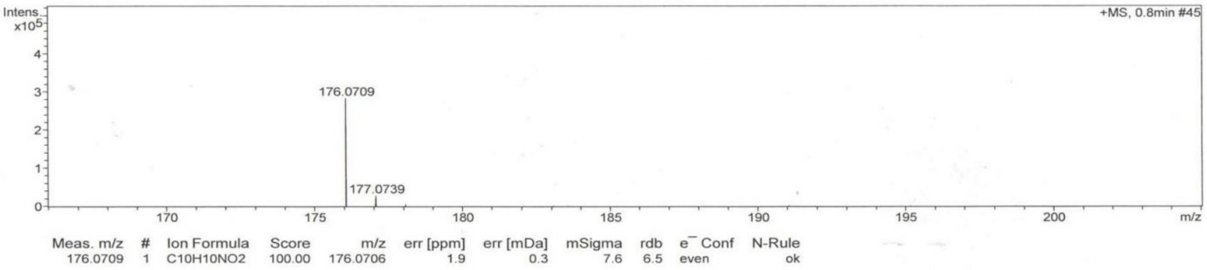

Figure S5. HRESIMS spectrum of **1**

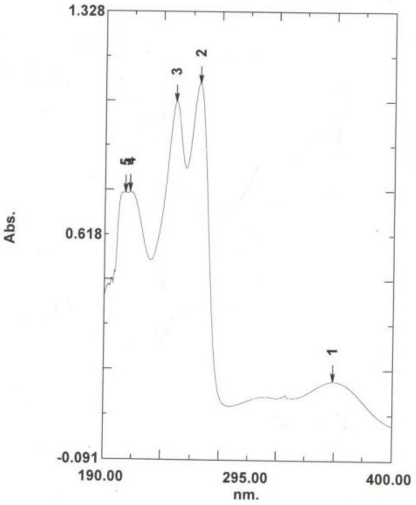

Figure S6. UV spectrum of **1**

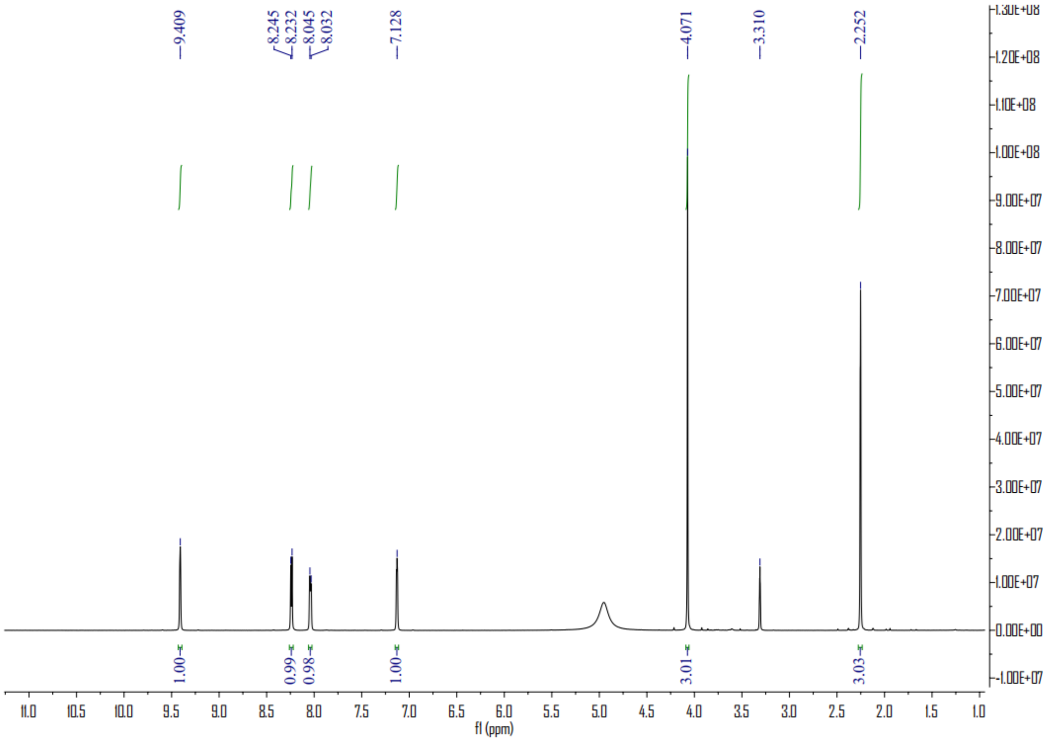

Figure S7. ^{1}H NMR spectrum of **2** in methanol-d_4

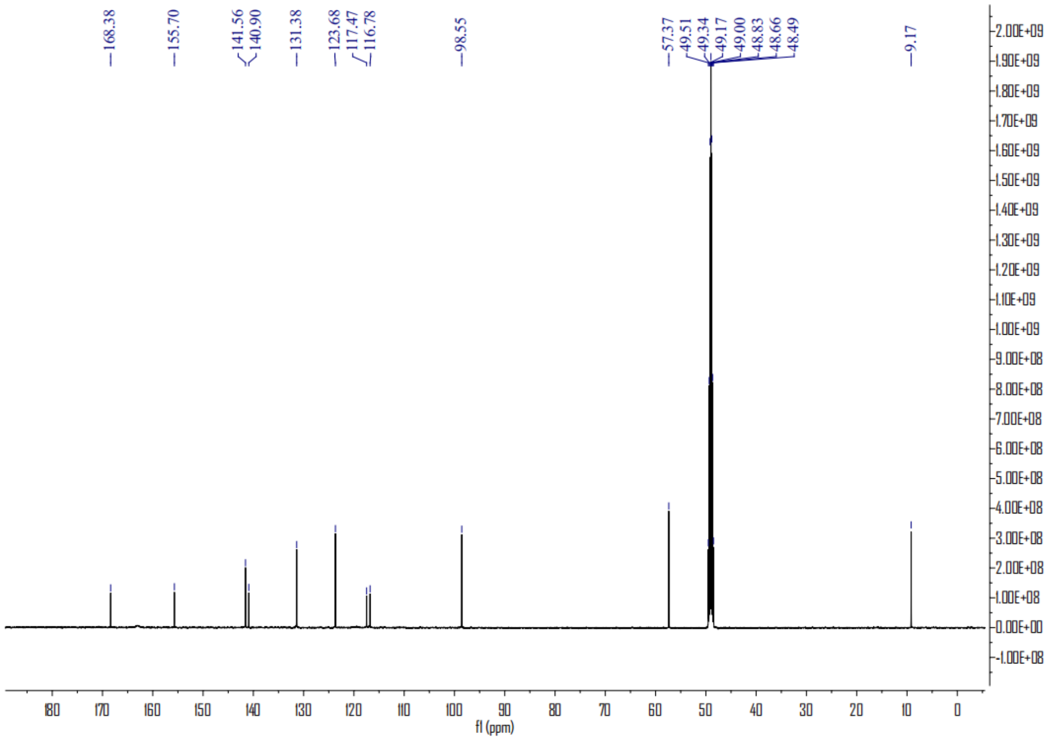

Figure S8. ^{13}C NMR spectrum of **2** in methanol-d_4

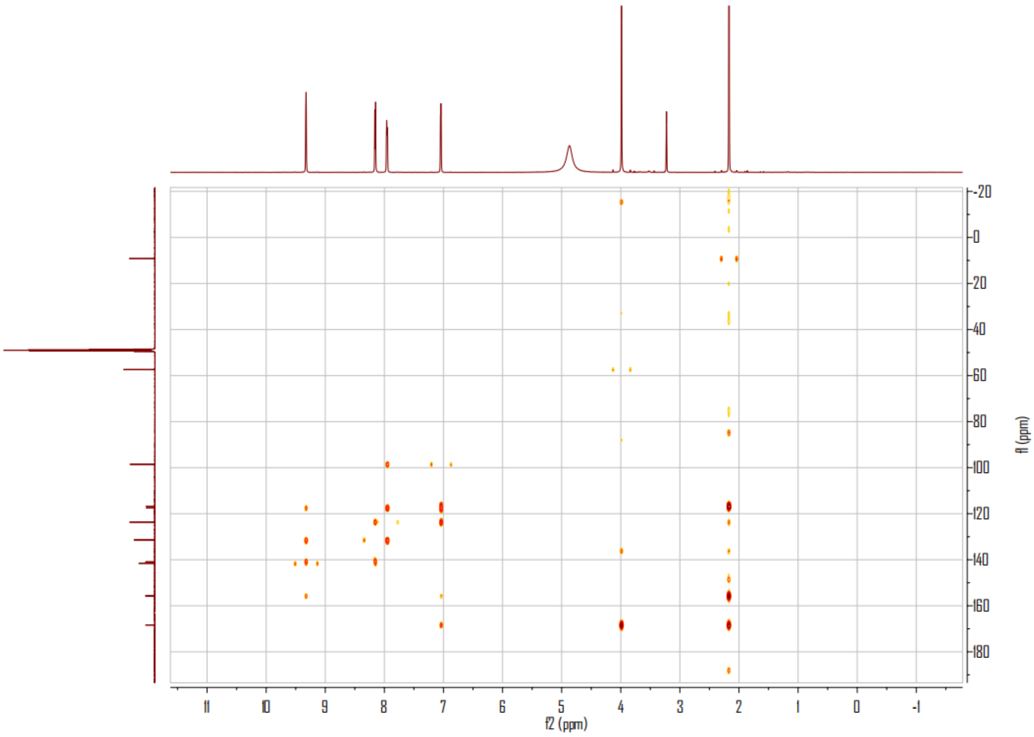

Figure S9. HMBC spectrum of **2** in methanol-d_4

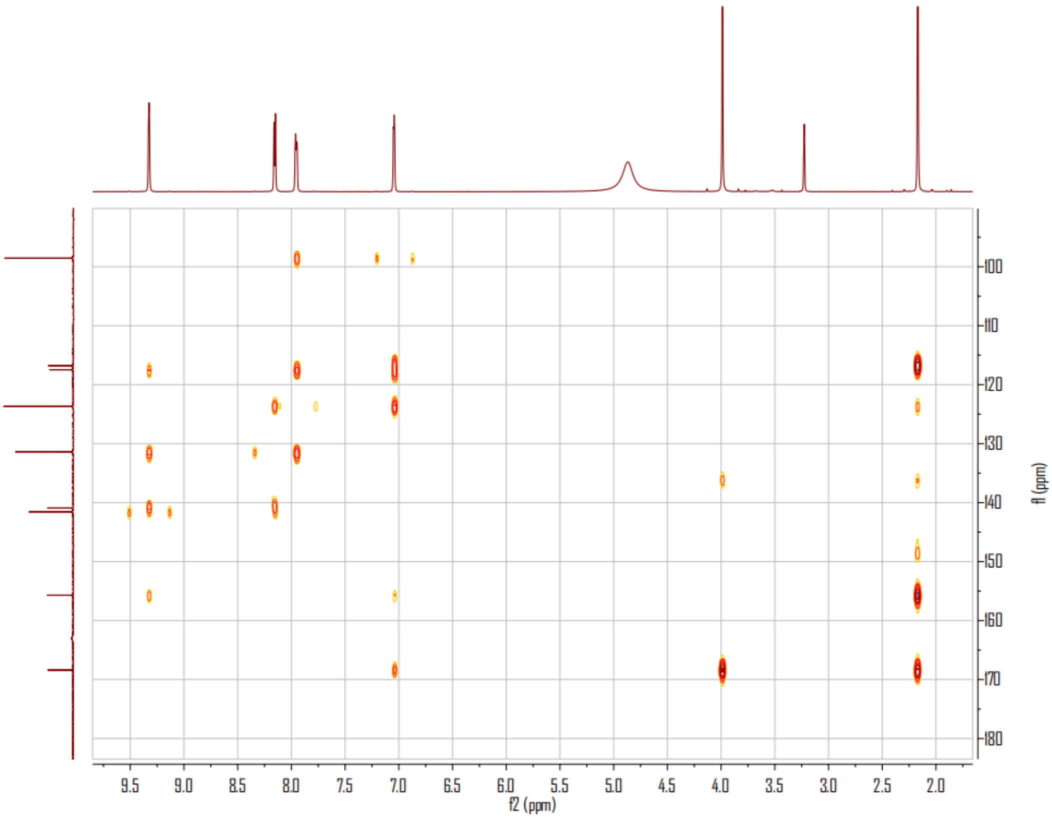

Figure S10. Partial HMBC spectrum of **2** in methanol-d_4

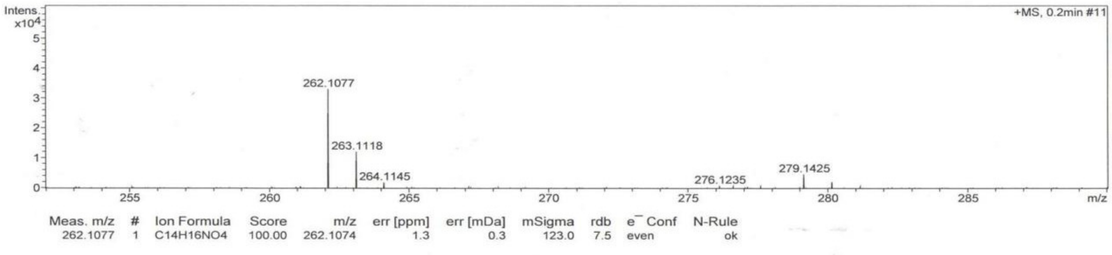

Figure S11. HRESIMS spectrum of **2**

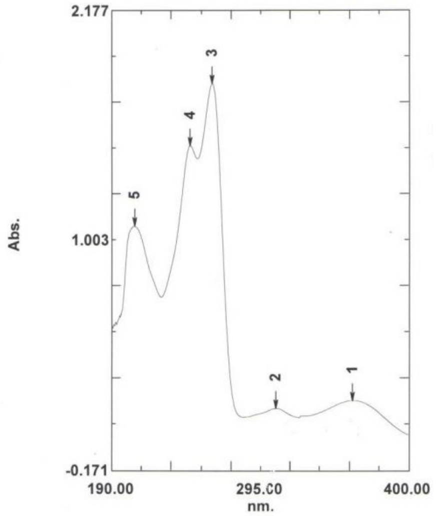

Figure S12. UV spectrum of **2.**

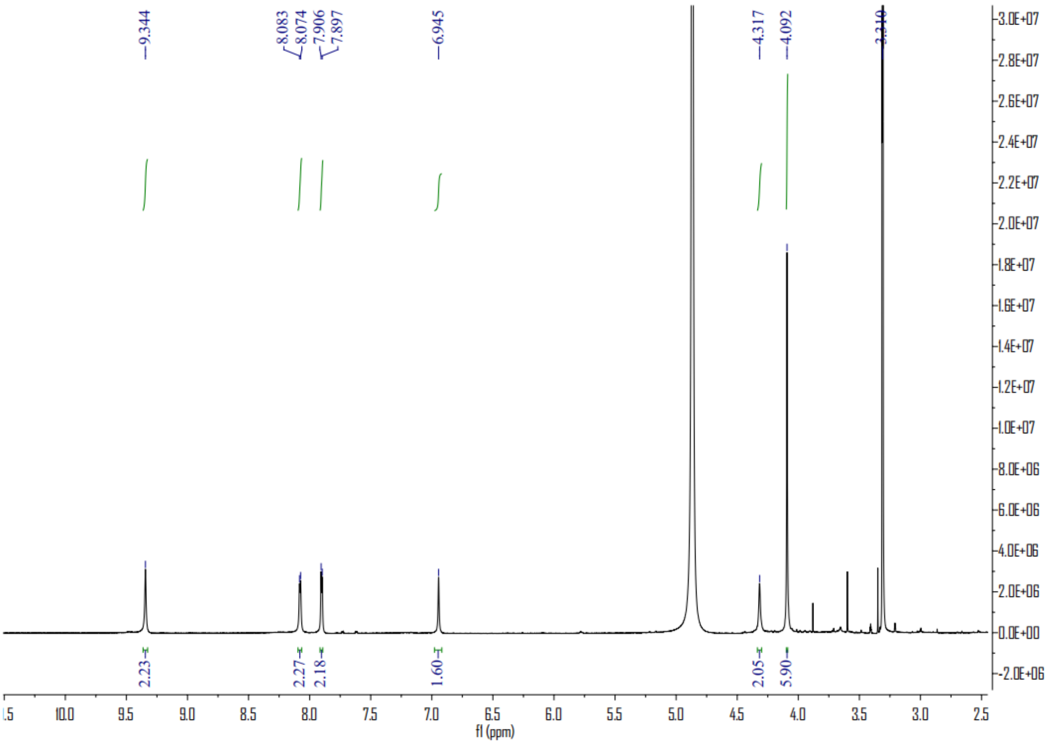

Figure S13. ^{1}H NMR spectrum of **3** in methanol-d_4

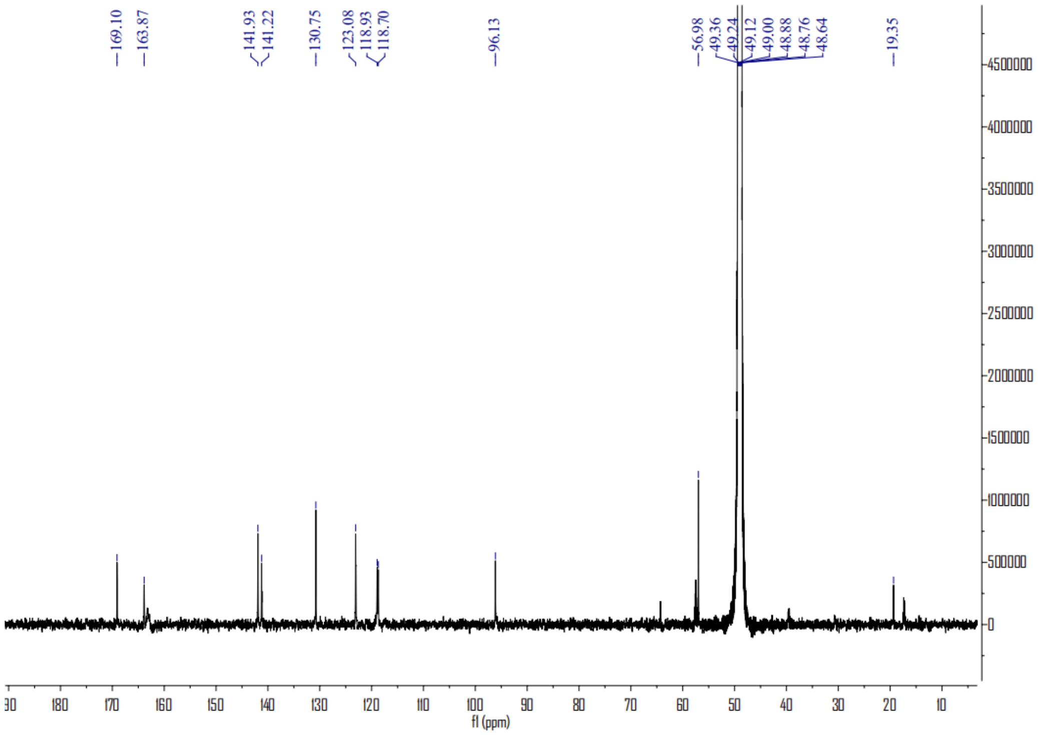

Figure S14. ^{13}C NMR spectrum of **3** in methanol-d_4

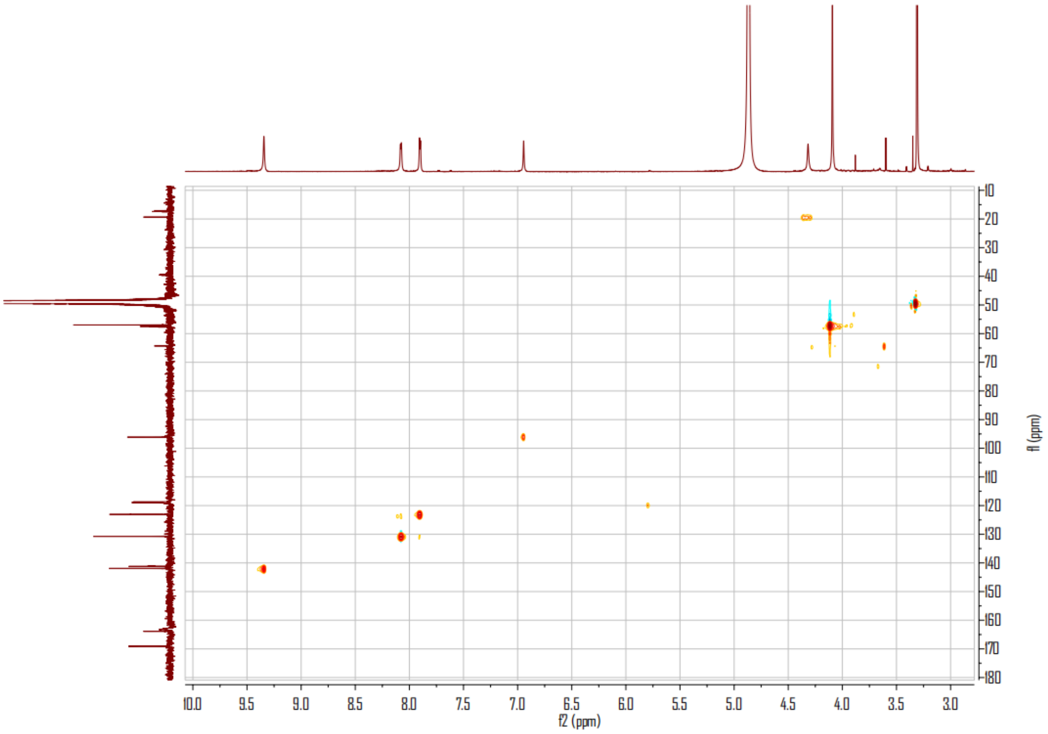

Figure S15. HSQC spectrum of **3** in methanol-*d*4

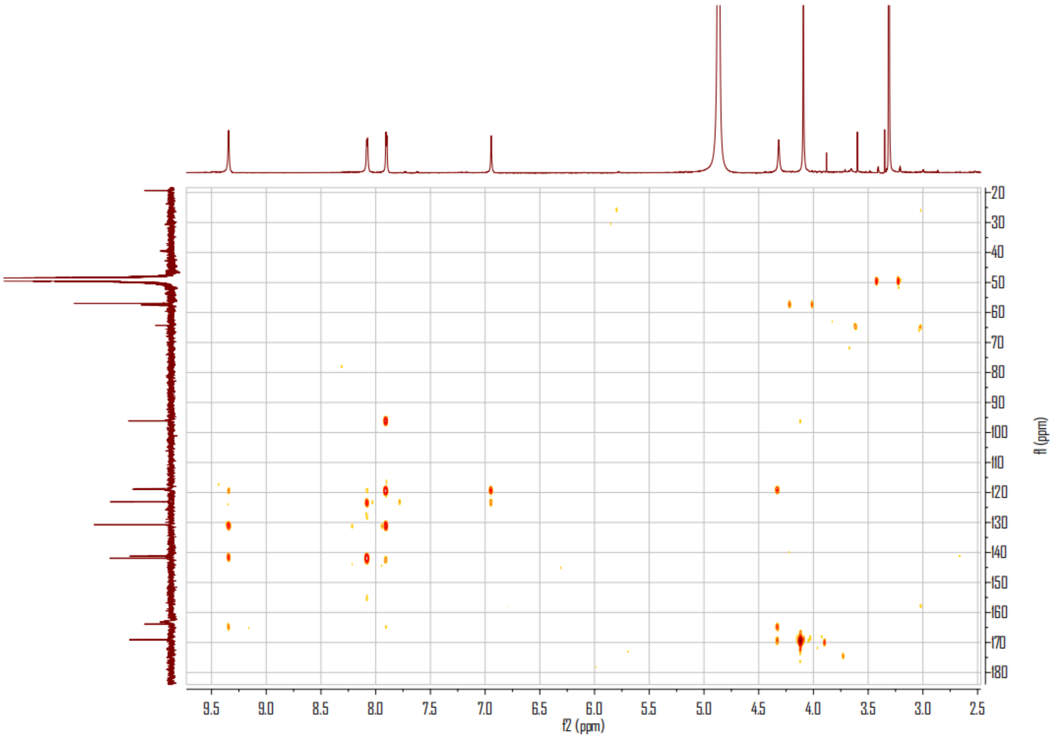

Figure S16. HMBC spectrum of **3** in methanol-*d*4

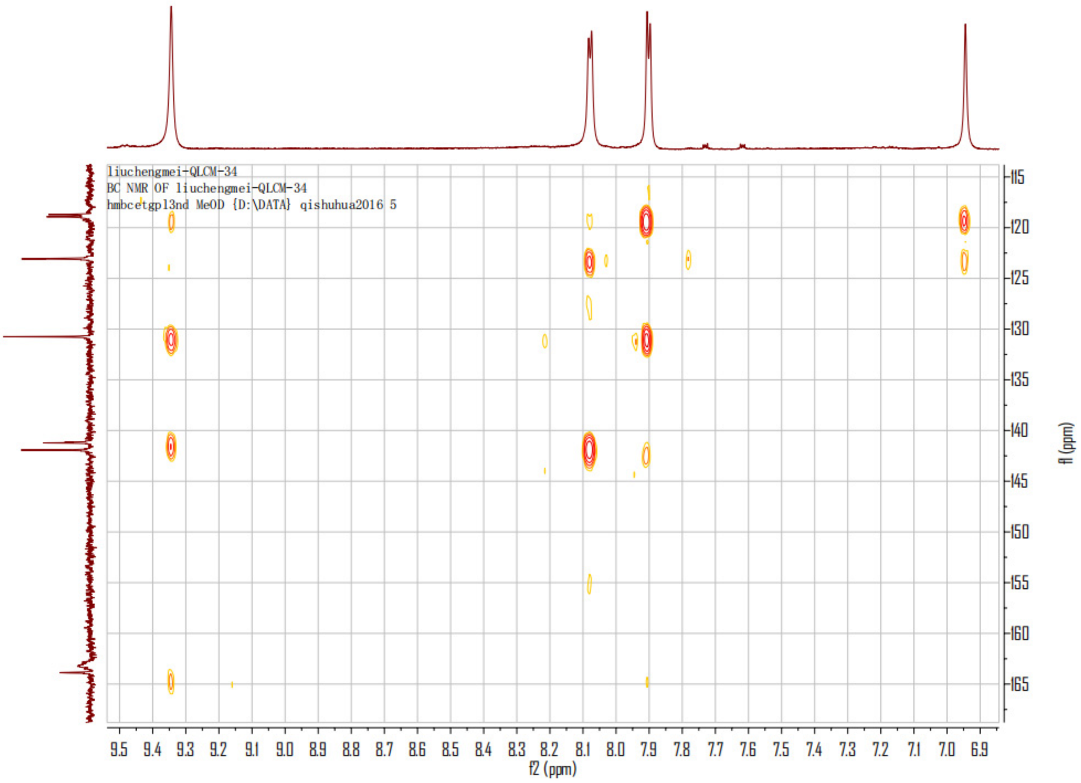

Figure S17. Partial HMBC spectrum of **3** in methanol-*d*4

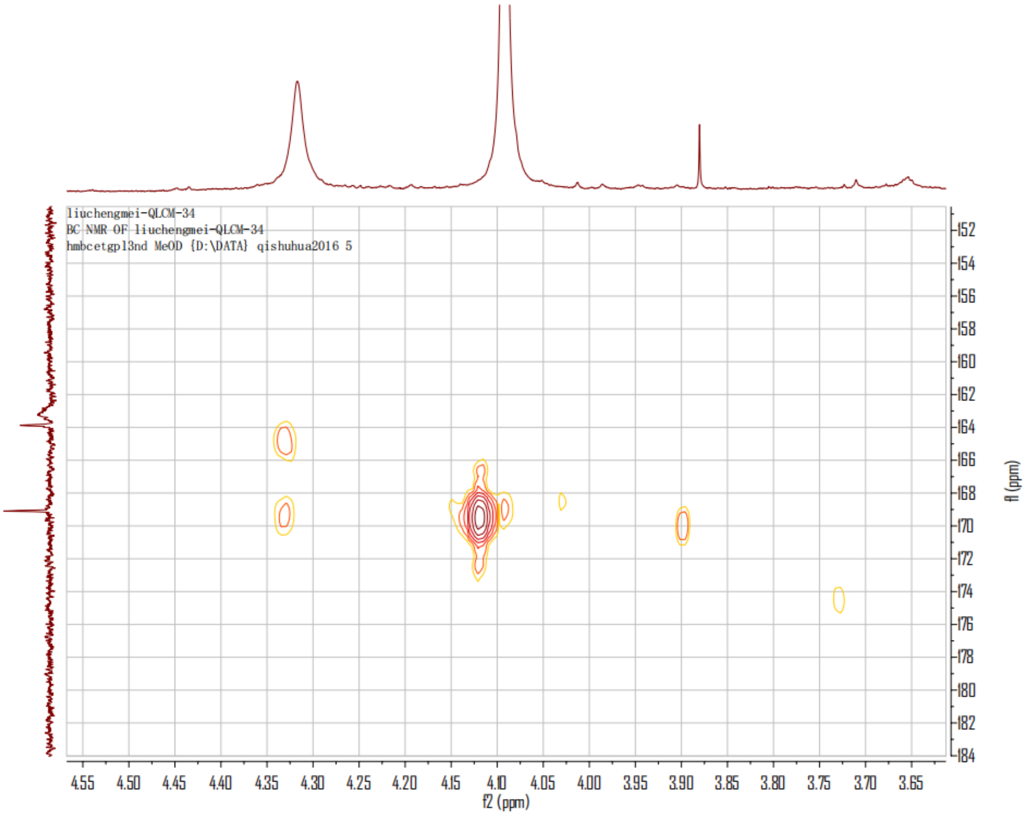

Figure S18. Partial HMBC spectrum of **3** in methanol-*d*4

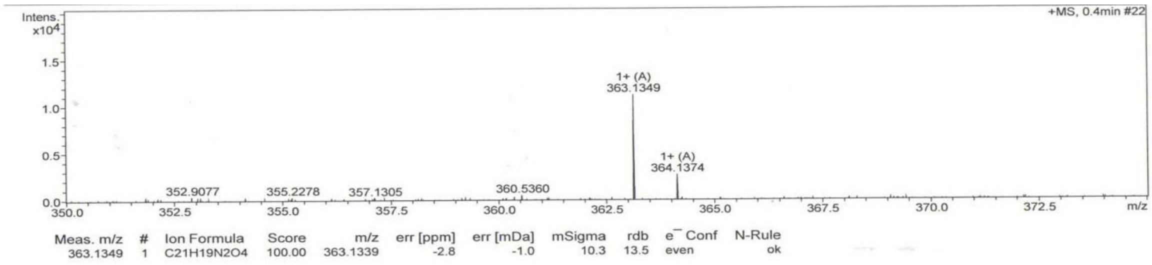

Figure S19. HRESIMS spectrum of **3**.

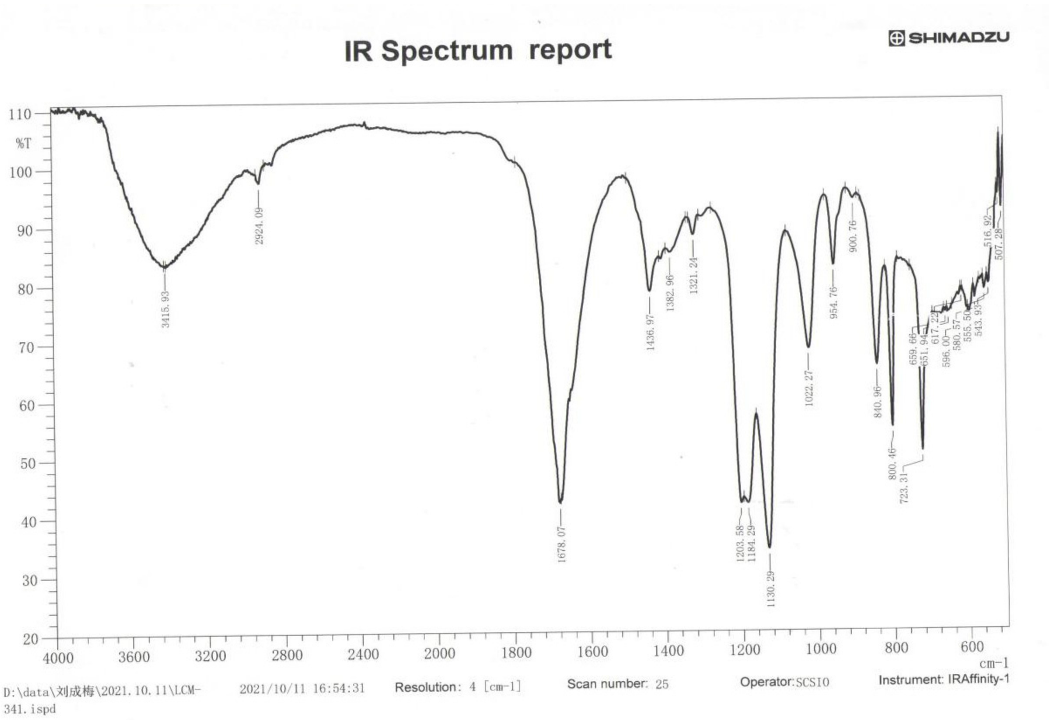

Figure S20. IR spectrum of **3**

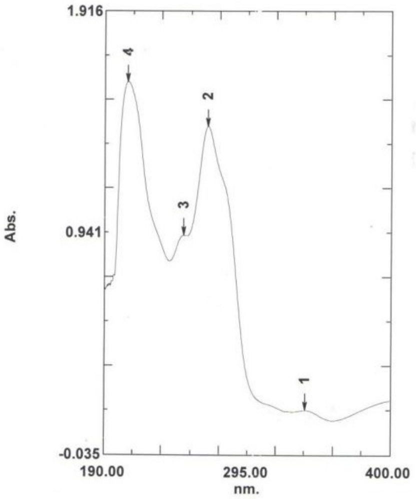

Figure S21. UV spectrum of **3**

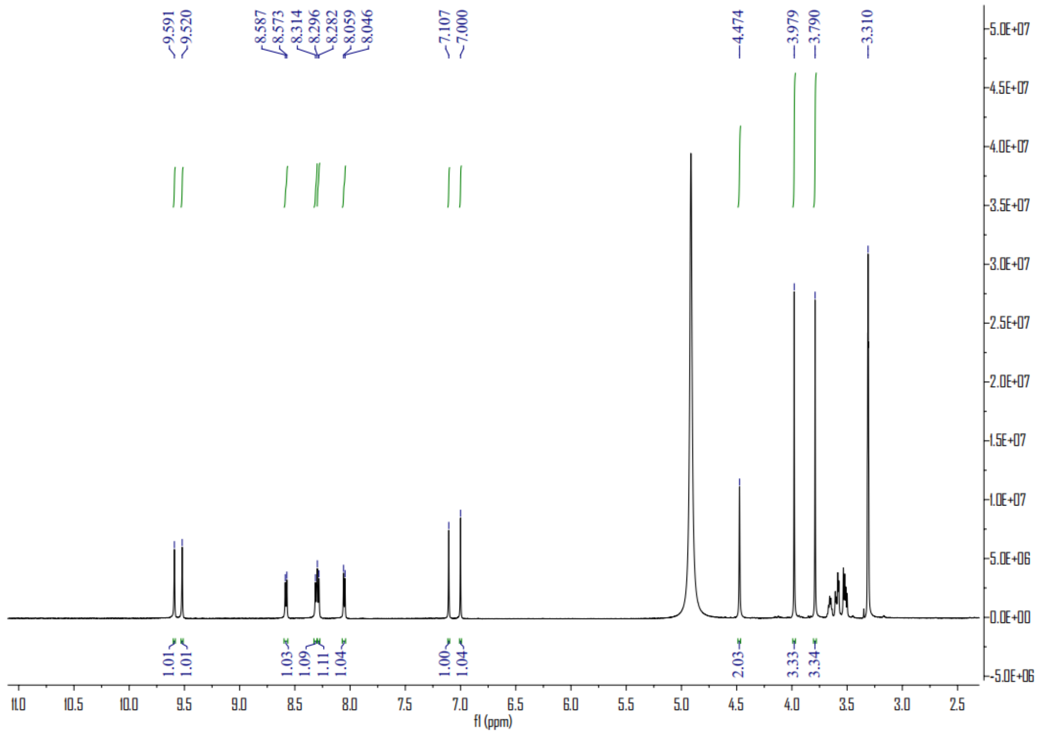

Figure S22. ^{1}H NMR spectrum of **4** in methanol-d_4

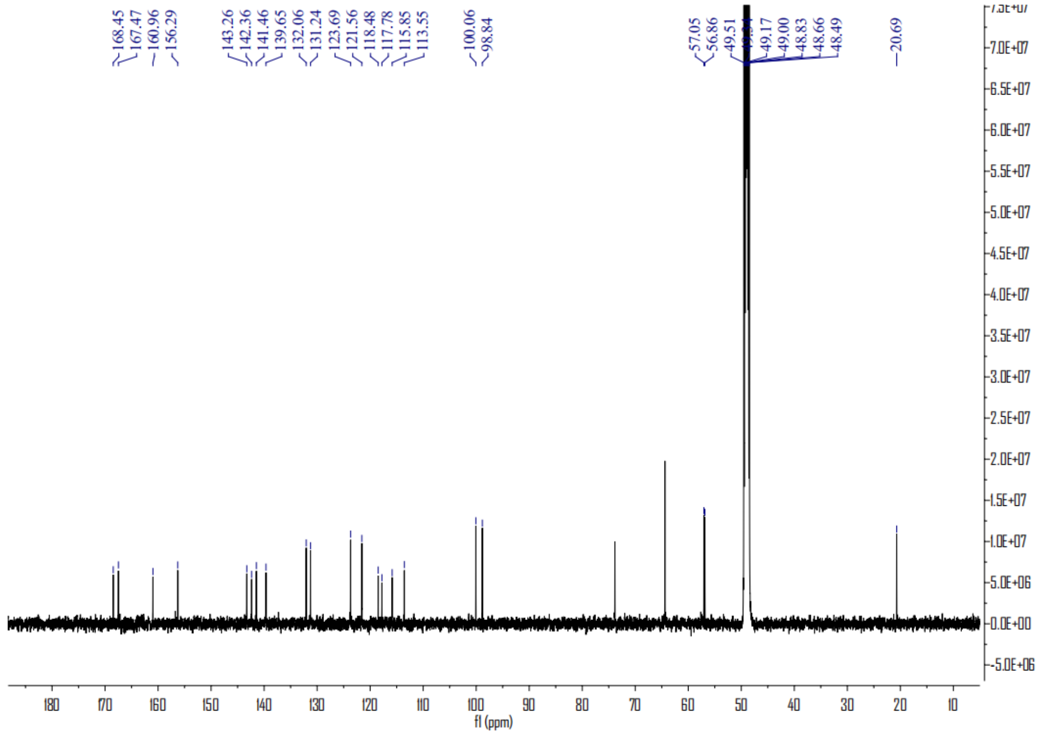

Figure S23. ^{13}C NMR spectrum of **4** in methanol-d_4

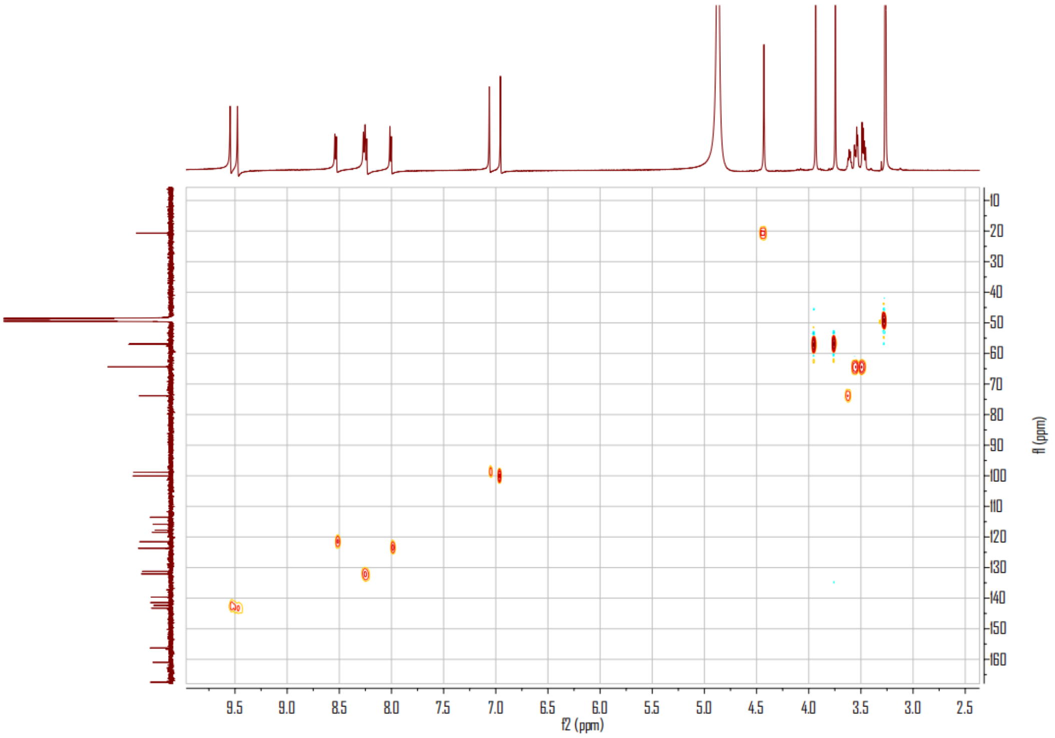

Figure S24. HSQC spectrum of **4** in methanol-d_4

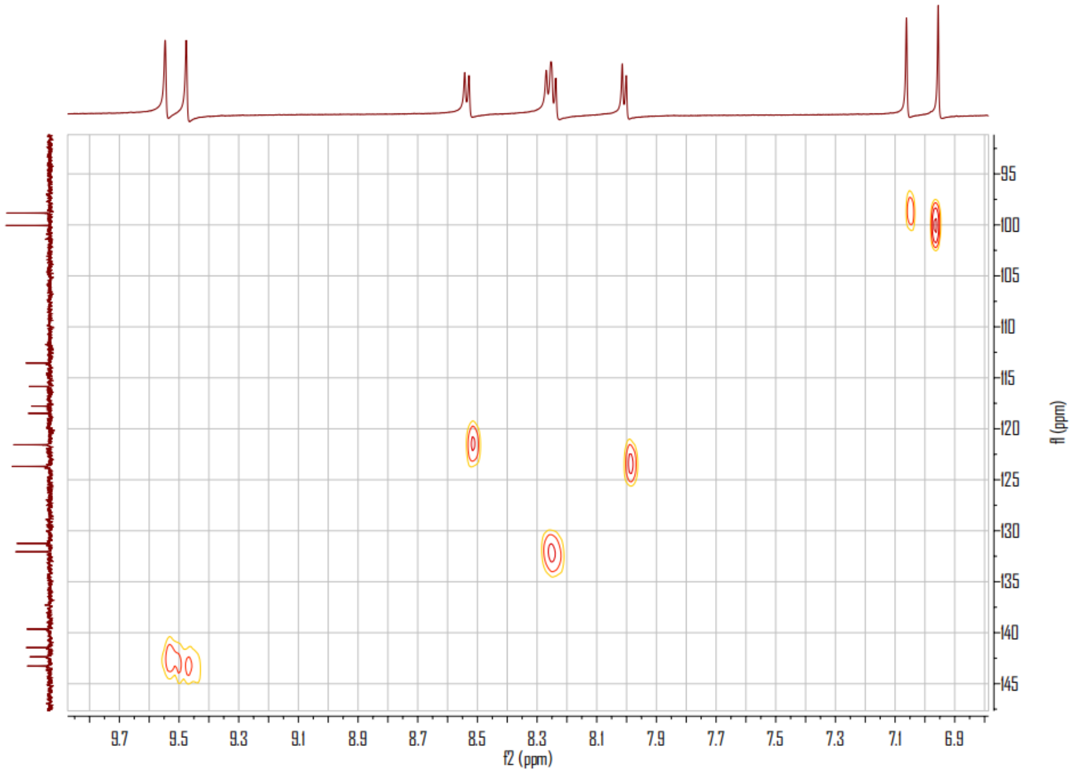

Figure S25. Partial HSQC spectrum of **4** in methanol-d_4

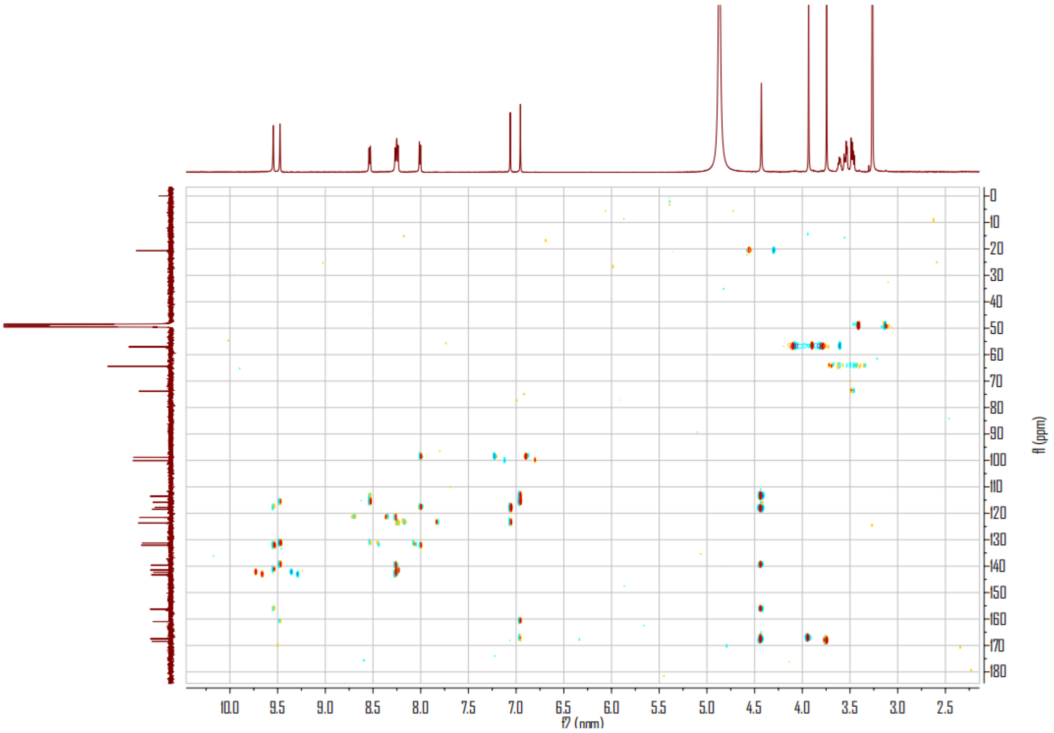

Figure S26. HMBC spectrum of **4** in methanol-d_4

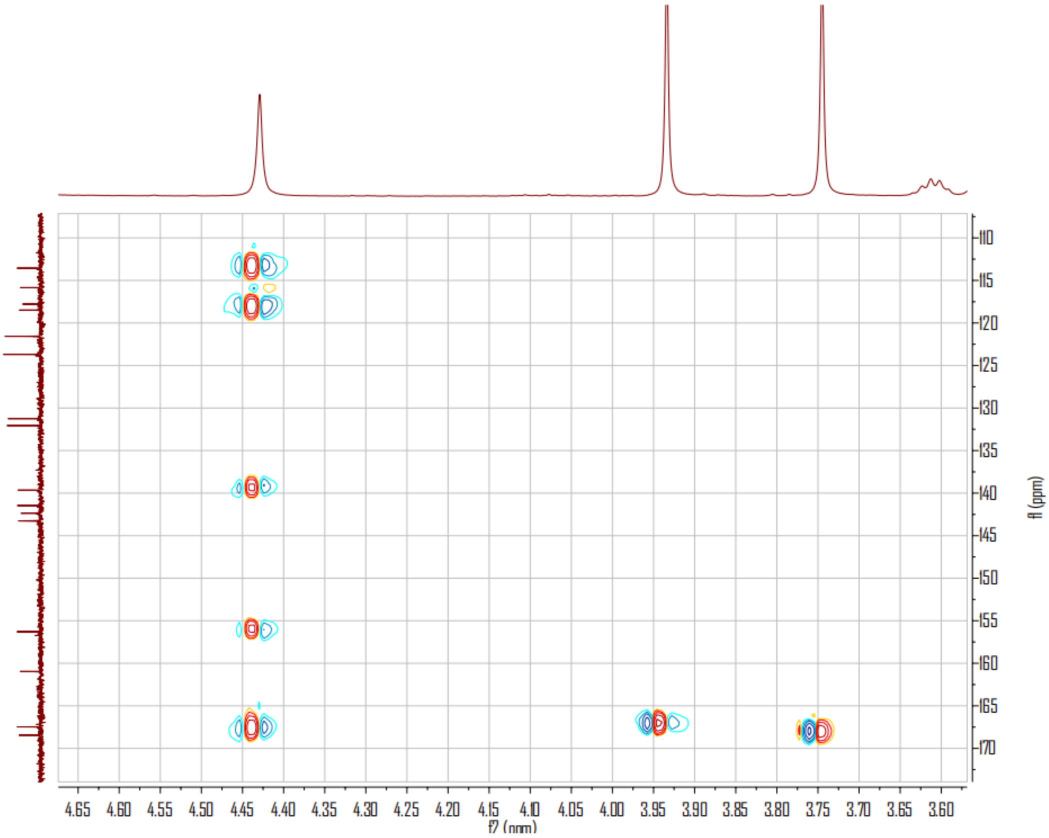

Figure S27. Partial HMBC spectrum of **4** in methanol-d_4

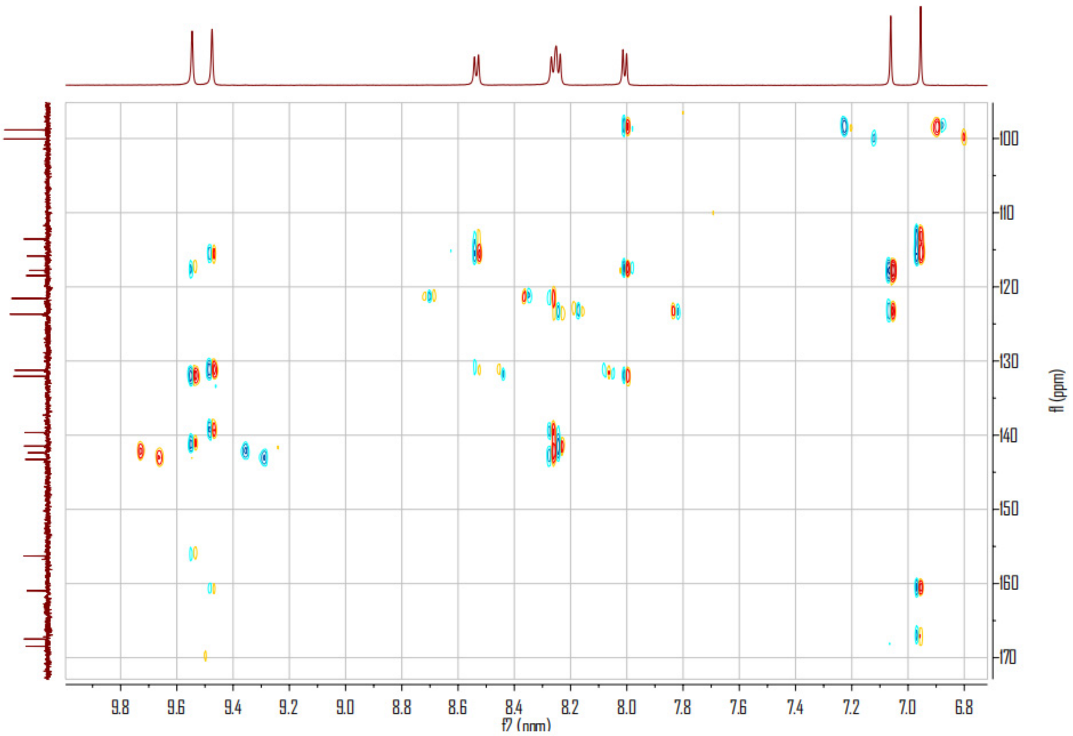

Figure S28. Partial HMBC spectrum of **4** in methanol-d_4

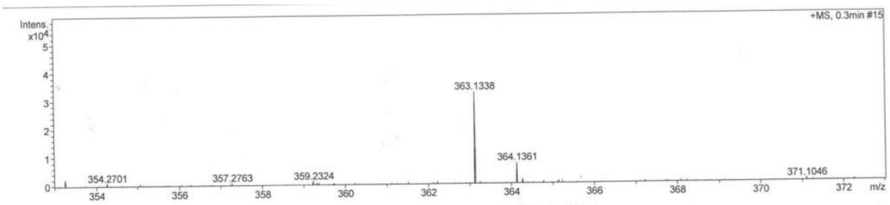

Meas. m/z	#	Ion Formula	Score	m/z	err [ppm]	err [mDa]	mSigma	rdb	e⁻ Conf	N-Rule
363.1338	1	C21H19N2O4	100.00	363.1339	0.4	0.1	9.0	13.5	even	ok
725.2586	1	C42H37N4O8	100.00	725.2606	2.8	2.0	94.8	26.5	even	ok

Figure S29. HRESIMS spectrum of **4**

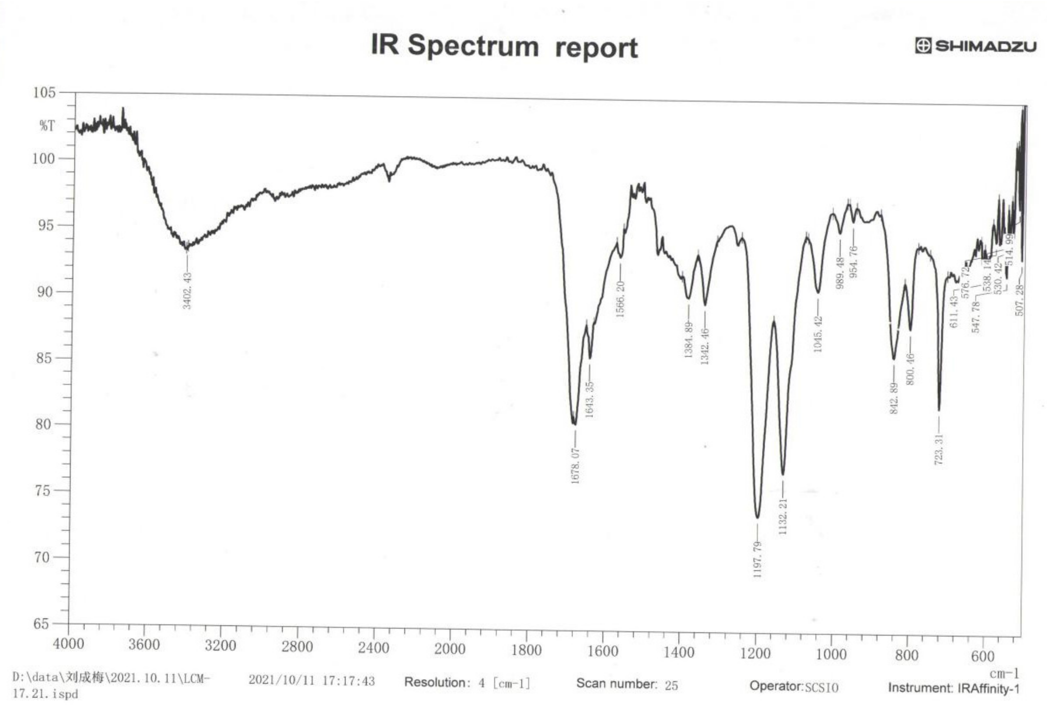

Figure S30. IR spectrum of **4**

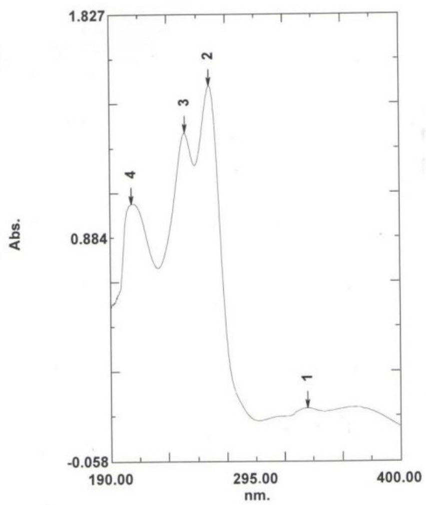

Figure S31. UV spectrum of **4**

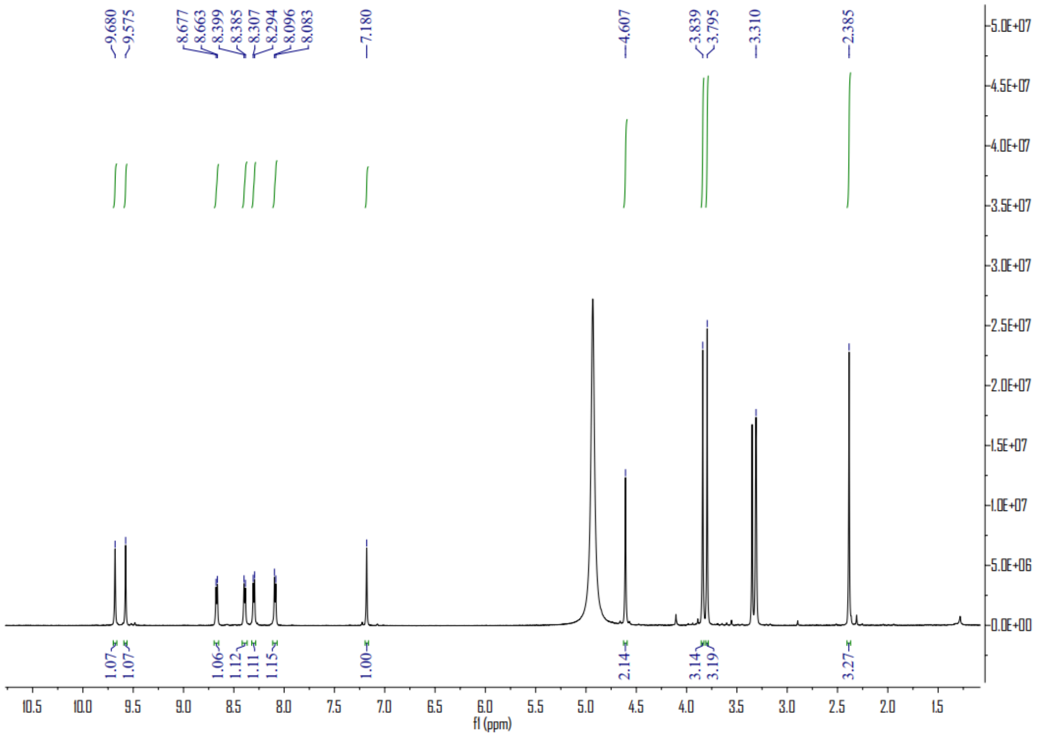

Figure S32. ^{1}H NMR spectrum of **5** in methanol-d_4

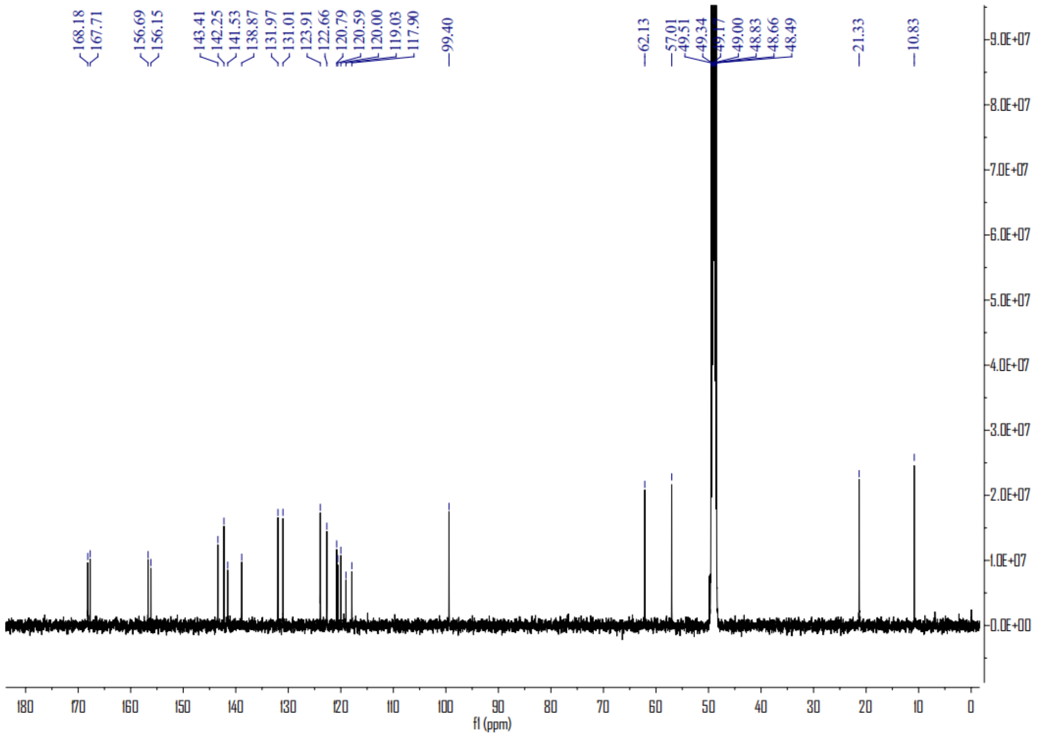

Figure S33. ^{13}C NMR spectrum of **5** in methanol-d_4

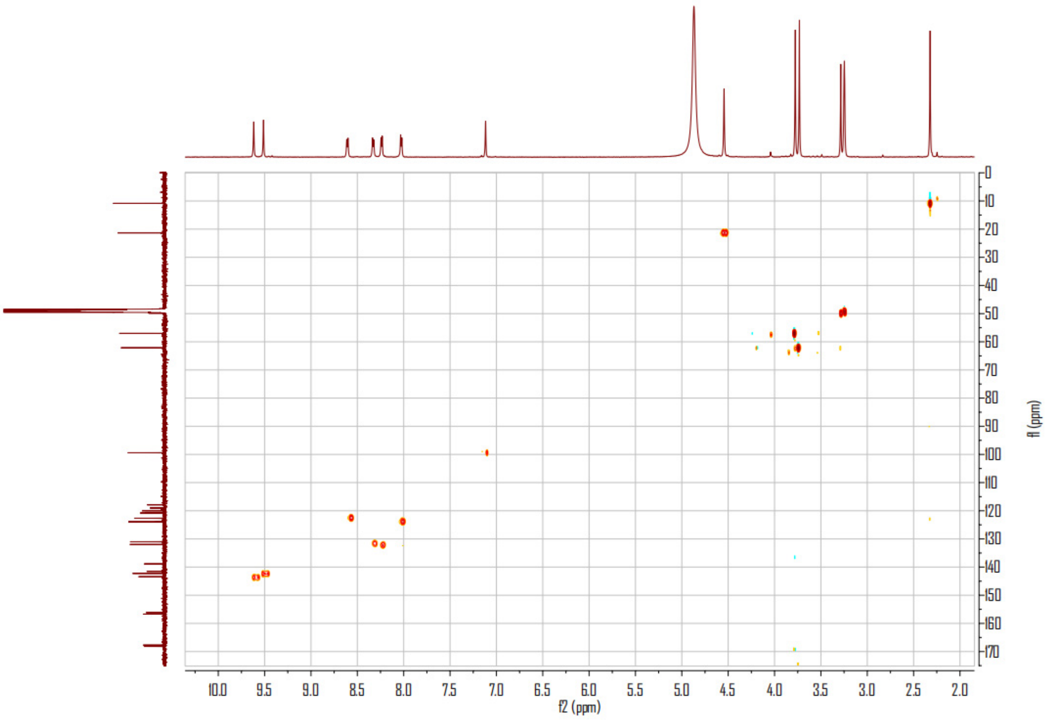

Figure S34. HSQC spectrum of **5** in methanol-d_4

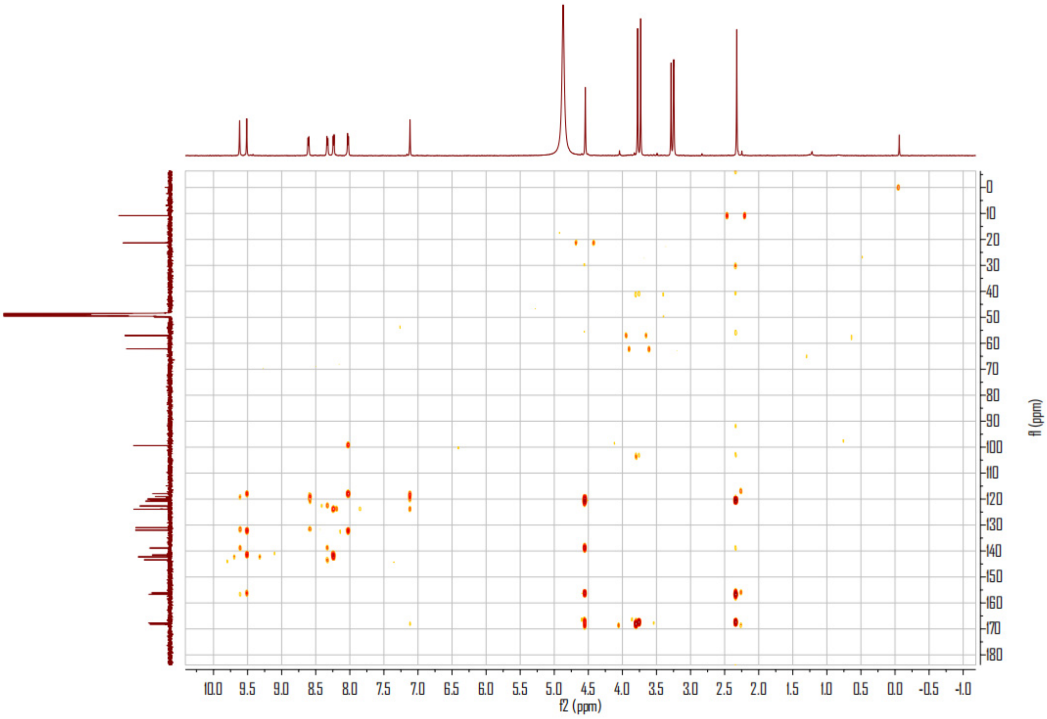

Figure S35. HMBC spectrum of **5** in methanol-d_4

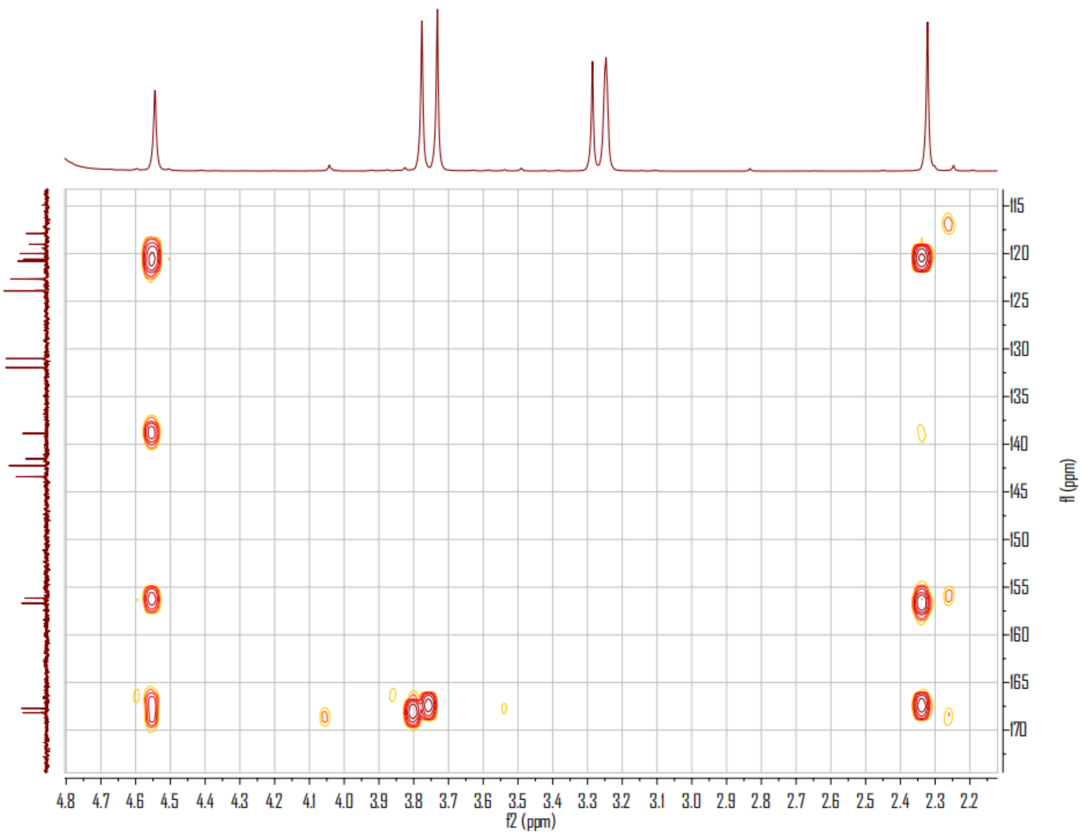

Figure S36. Partial HMBC spectrum of **5** in methanol-d_4

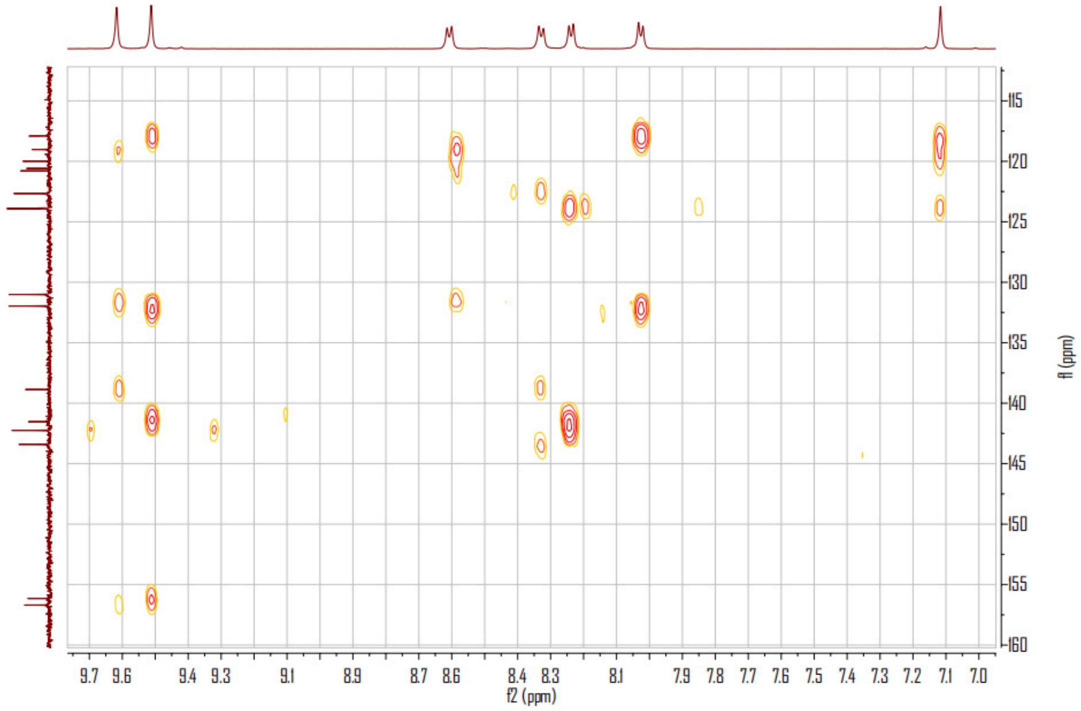

Figure S37. Partial HMBC spectrum of **5** in methanol-d_4

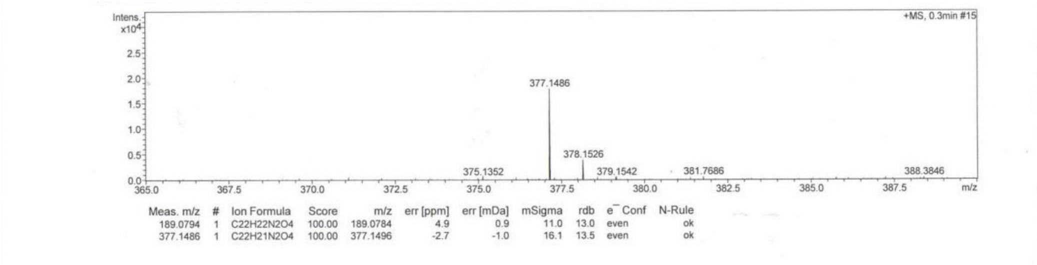

Figure S38. HRESIMS spectrum of **5**

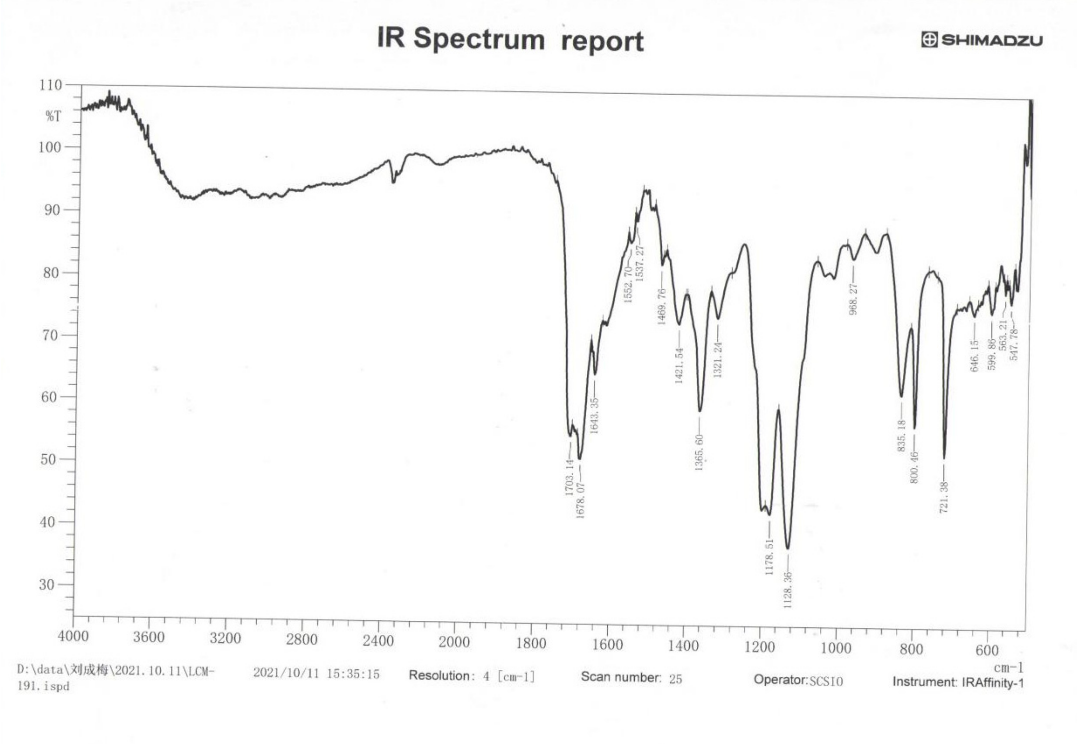

Figure S39. IR spectrum of **5**

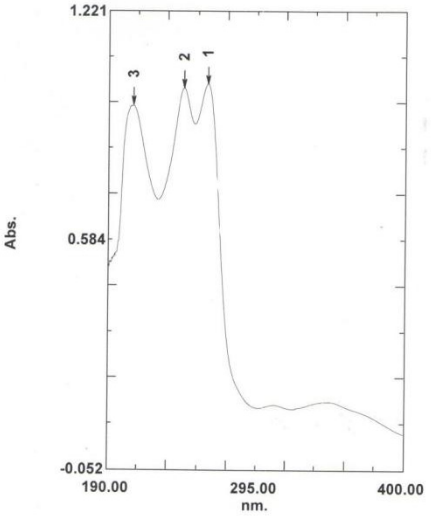

Figure S40. UV spectrum of **5**

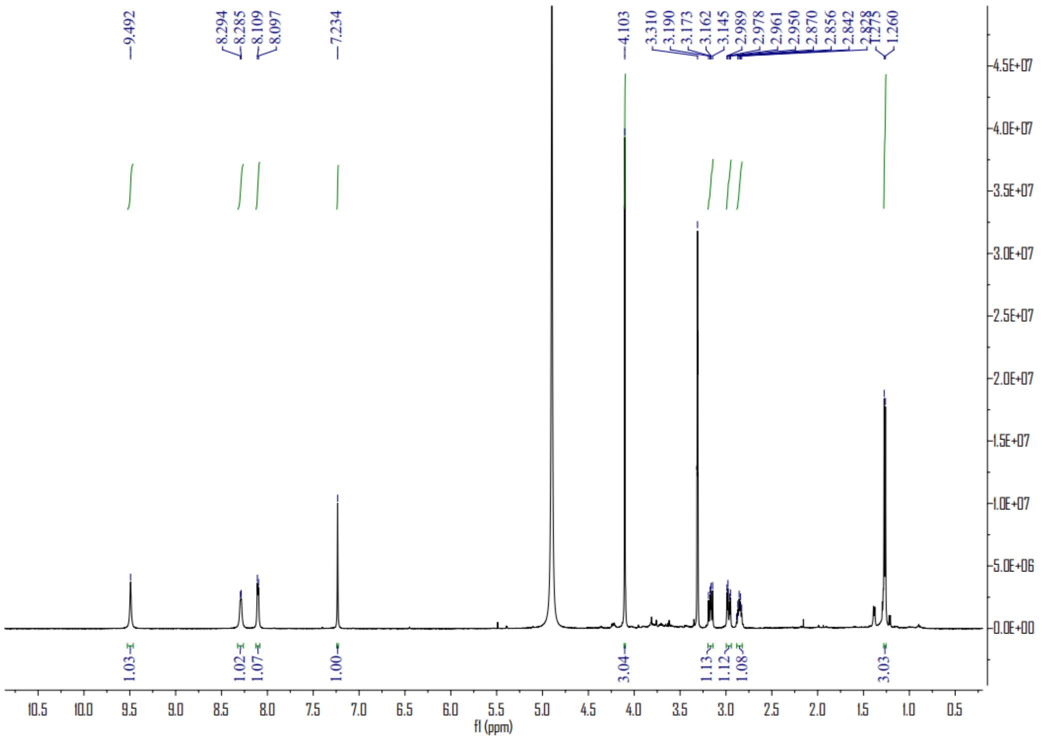

Figure S41. ^{1}H NMR spectrum of **6** in methanol-d_4

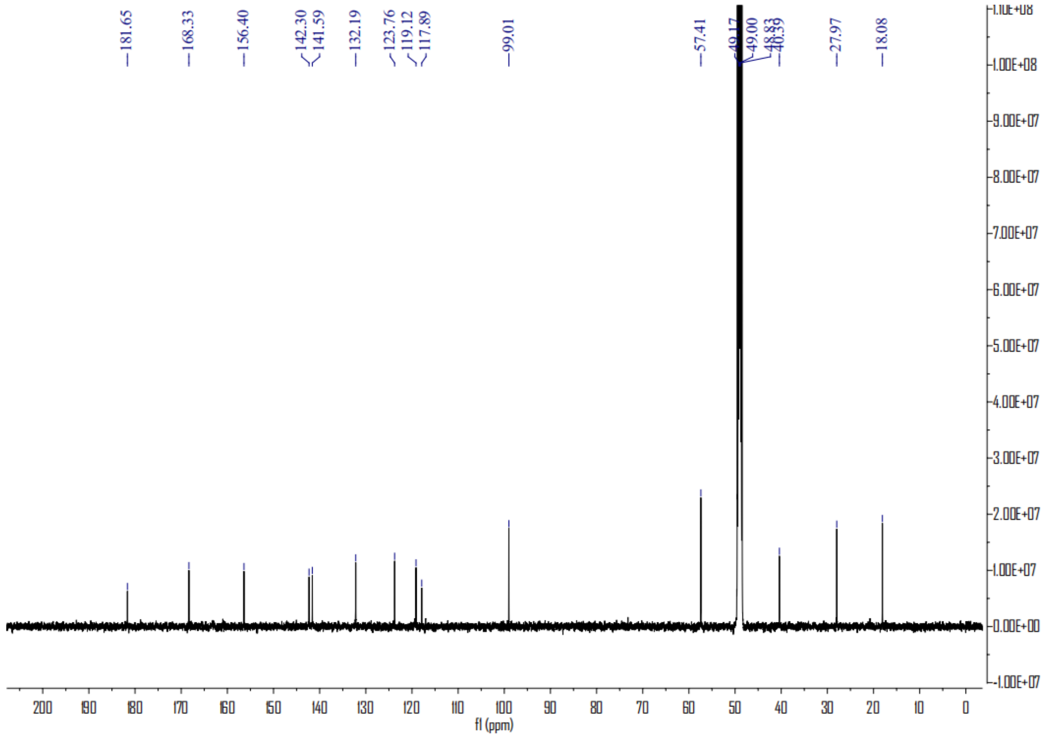

Figure S42. ^{13}C NMR spectrum of **6** in methanol-d_4

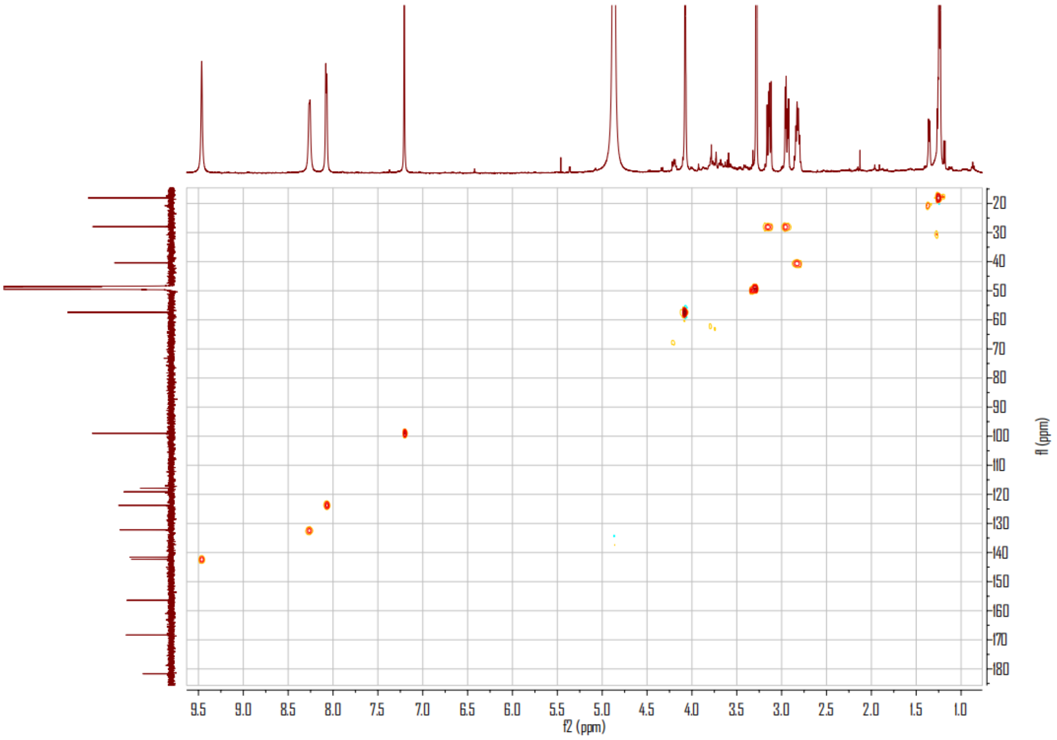

Figure S43. HSQC spectrum of **6** in methanol-d_4

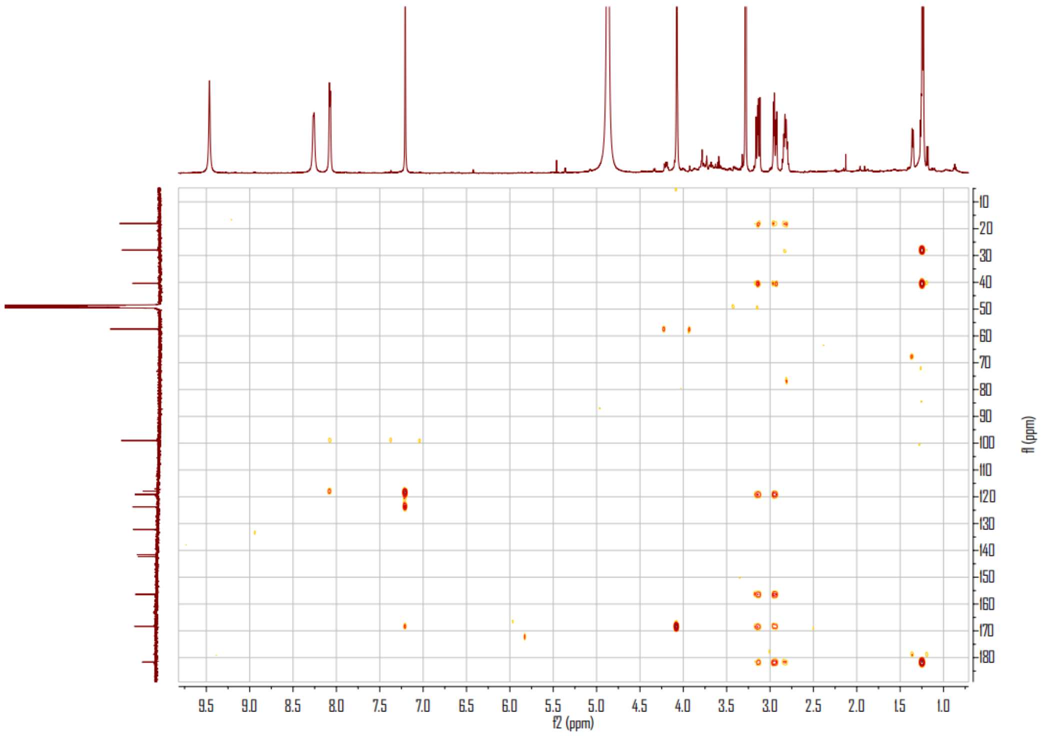

Figure S44. HMBC spectrum of **6** in methanol-d_4

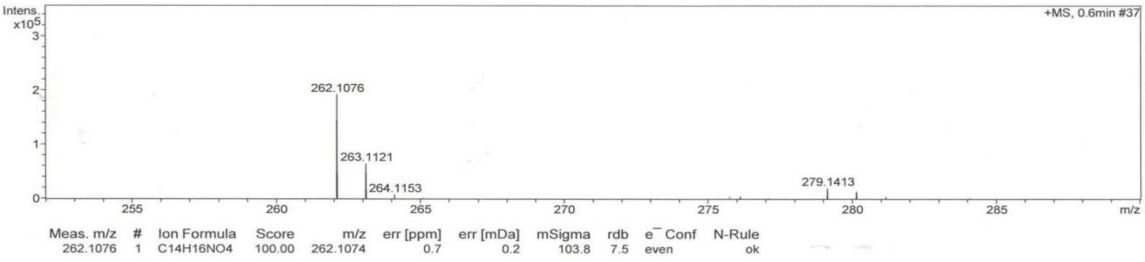

Figure S45. HRESIMS spectrum of **6**

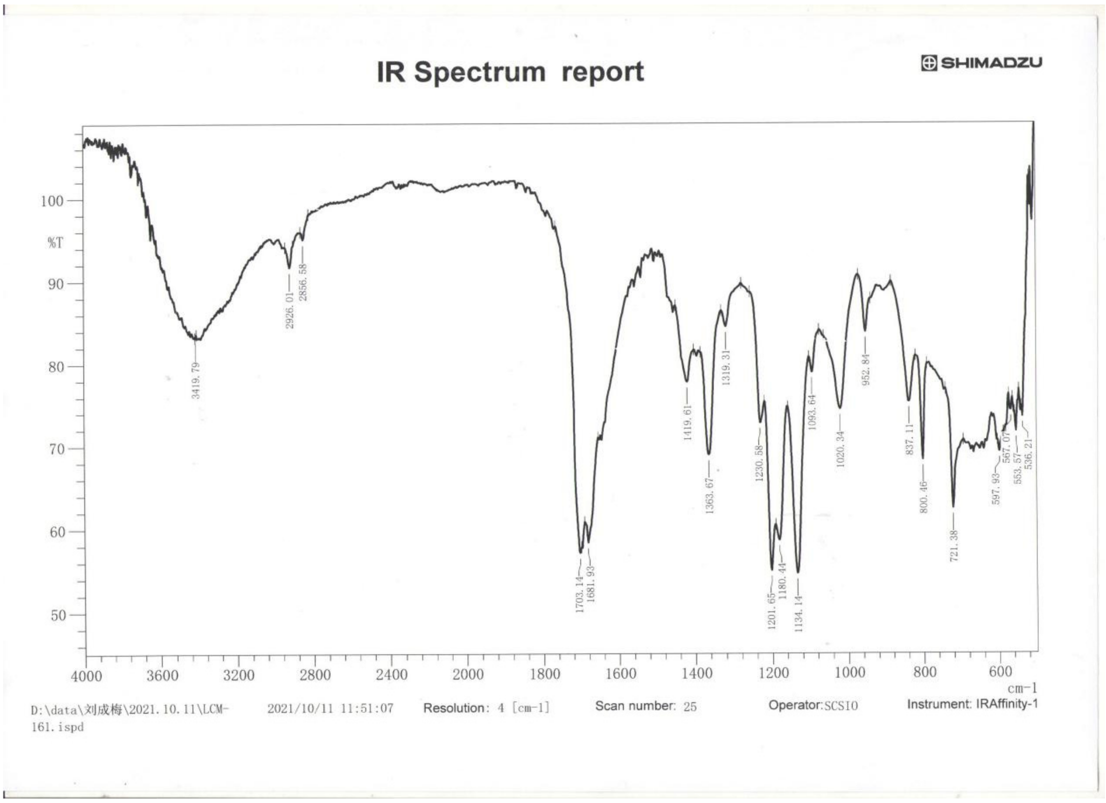

Figure S46. IR spectrum of **6**

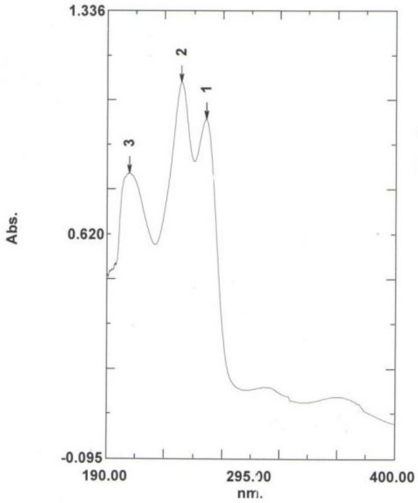

Figure S47. UV spectrum of **6**

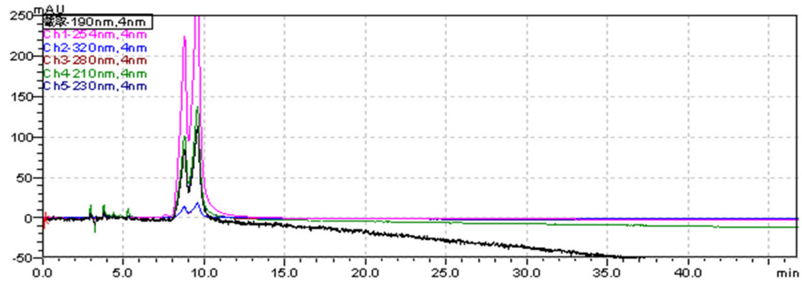

Figure S48. Chirality analysis of compound **6**

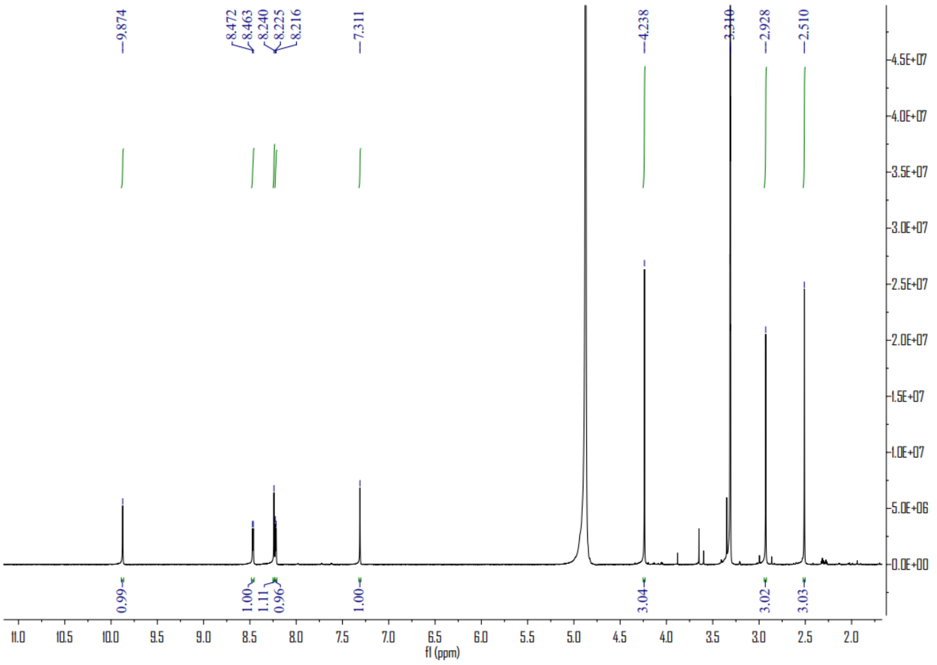

Figure S49. ^{1}H NMR spectrum of **7** in methanol-d_4

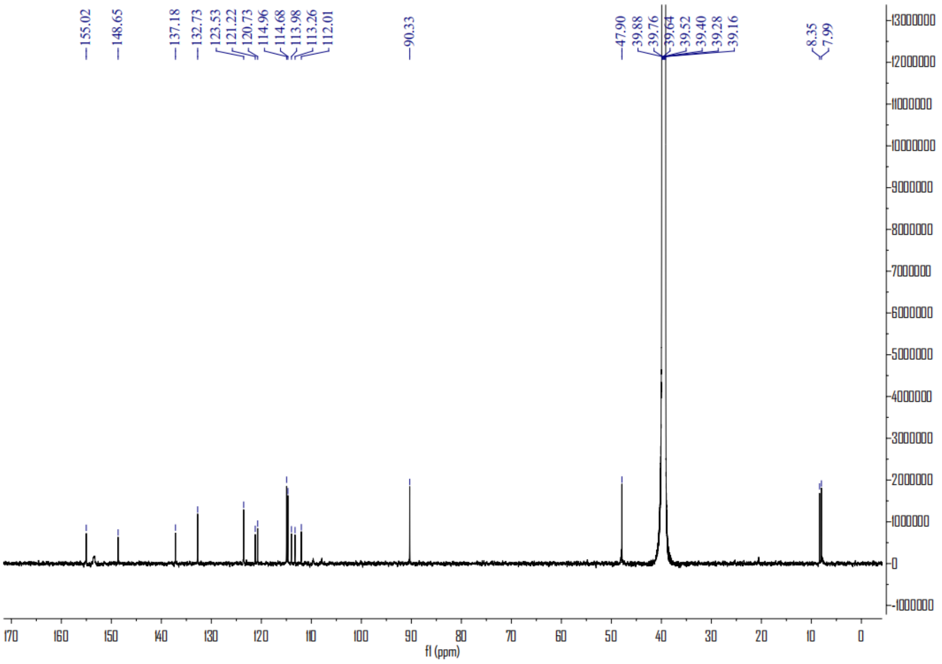

Figure S50. ^{13}C NMR spectrum of **7** in methanol-d_4

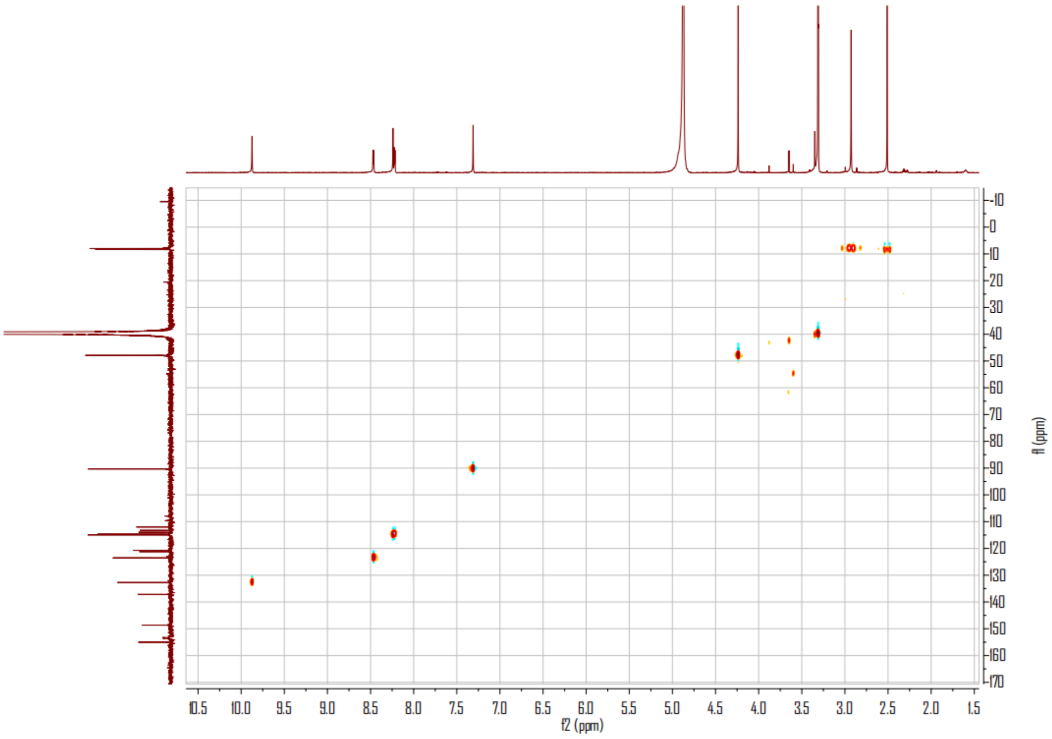

Figure S51. HSQC spectrum of **7** in methanol-d_4

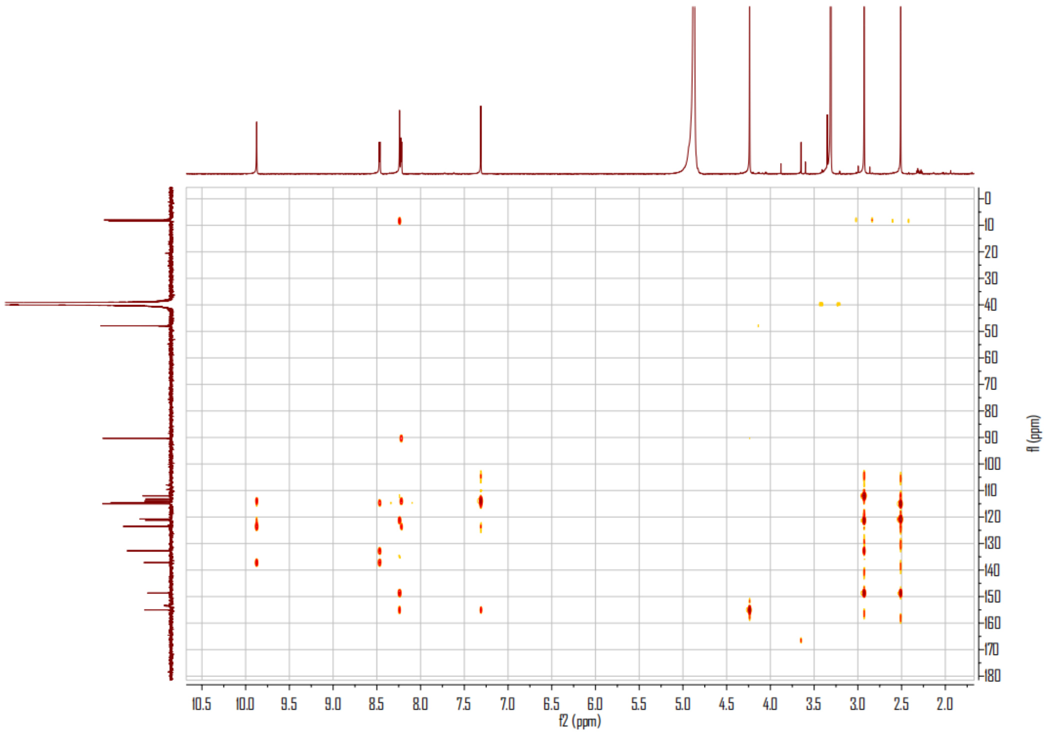

Figure S52. HMBC spectrum of **7** in methanol-d_4

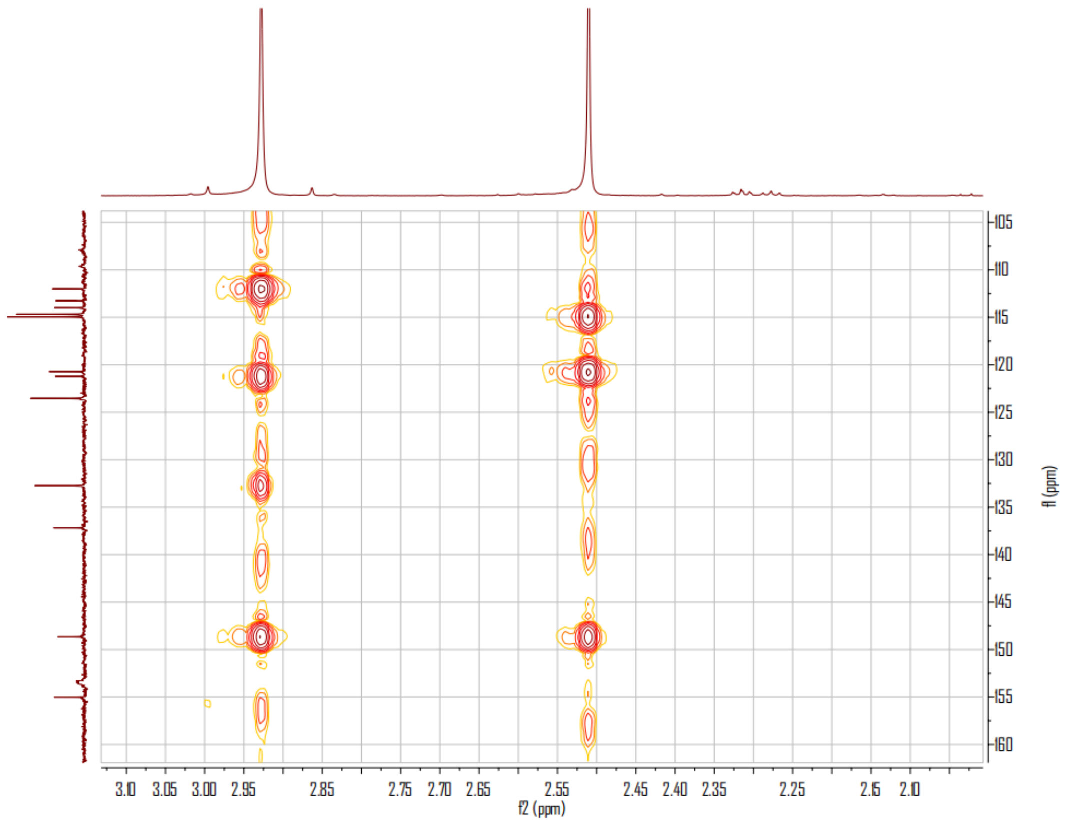

Figure S53. Partial HMBC spectrum of **7** in methanol-d_4

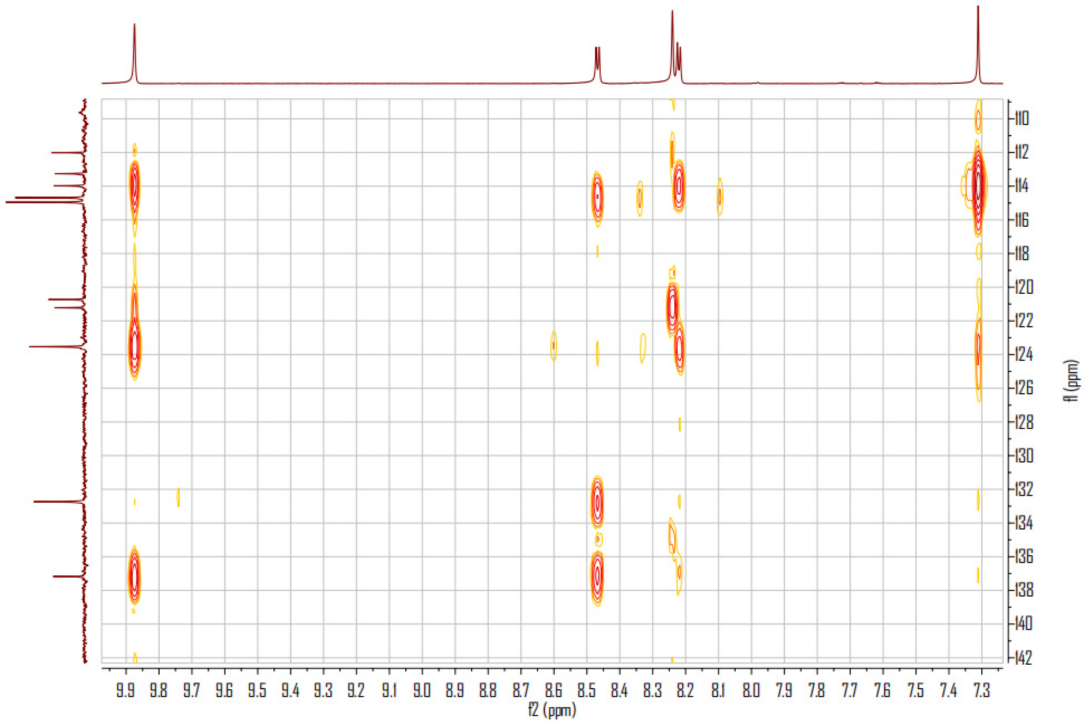

Figure S54. Partial HMBC spectrum of **7** in methanol-d_4

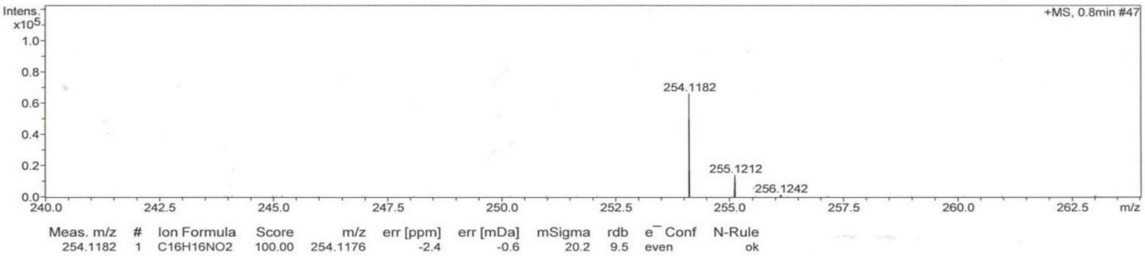

Figure S55. HRESIMS spectrum of **7**

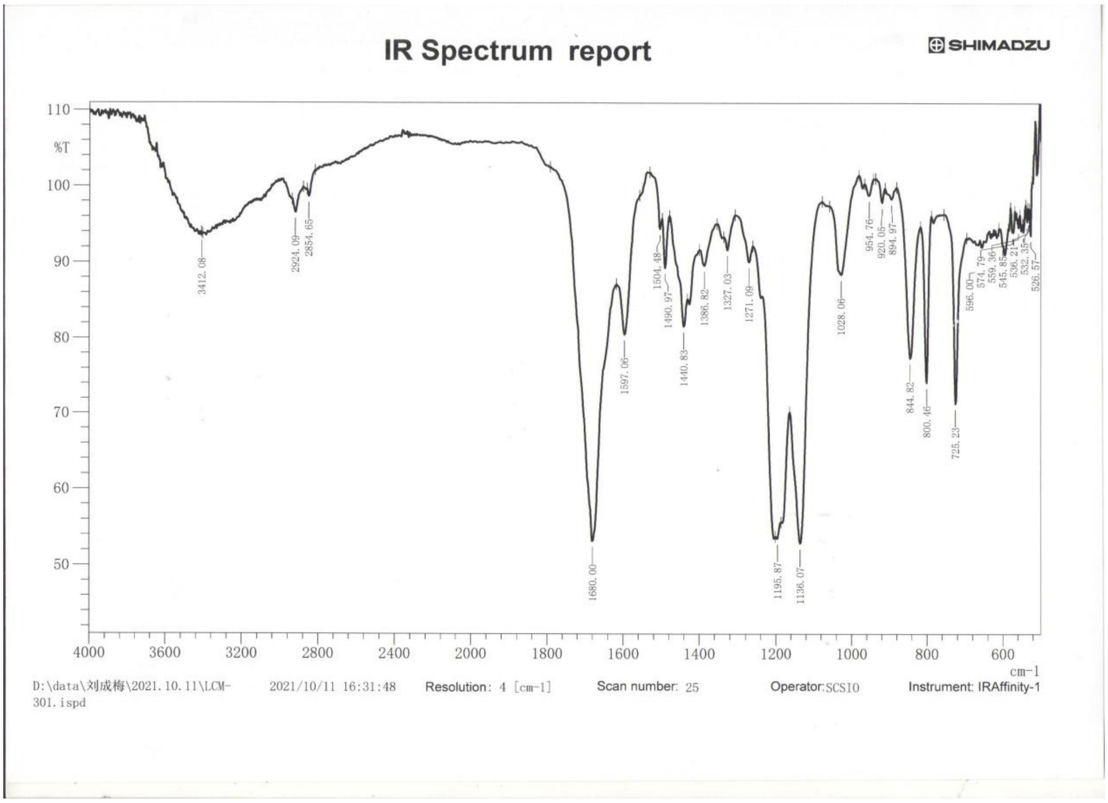

Figure S56. IR spectrum of **7**

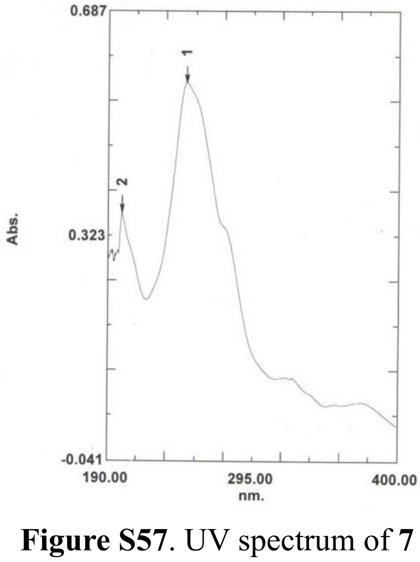

Figure S57. UV spectrum of **7**

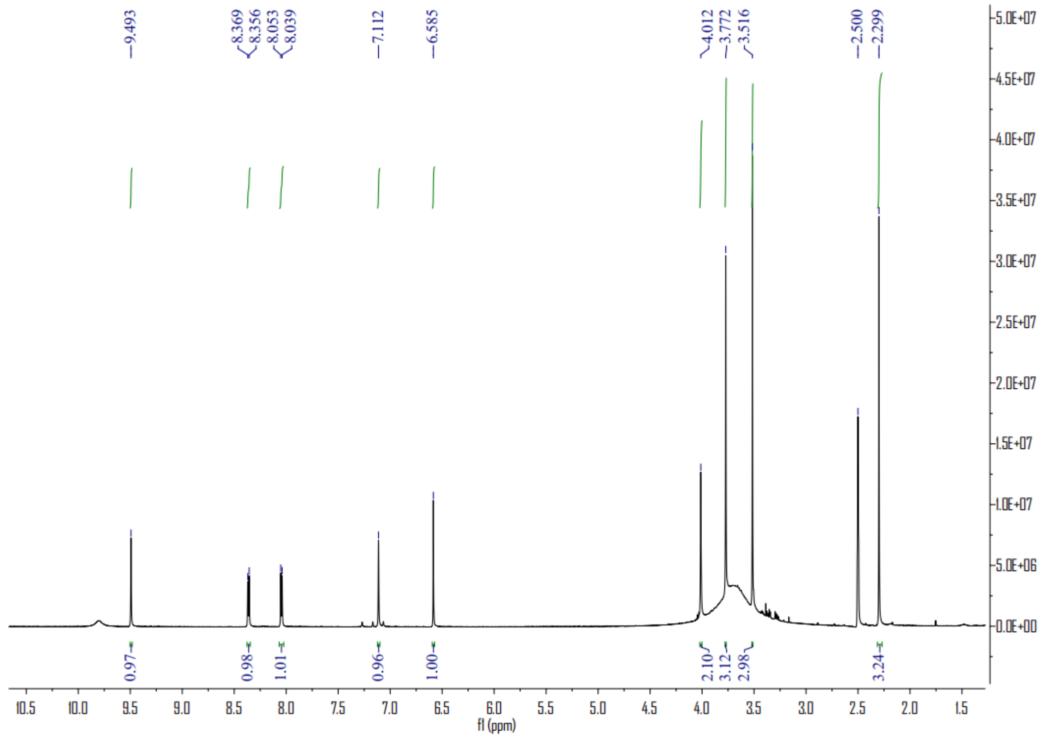

Figure S58. ^{1}H NMR spectrum of **8** in DMSO-d_6

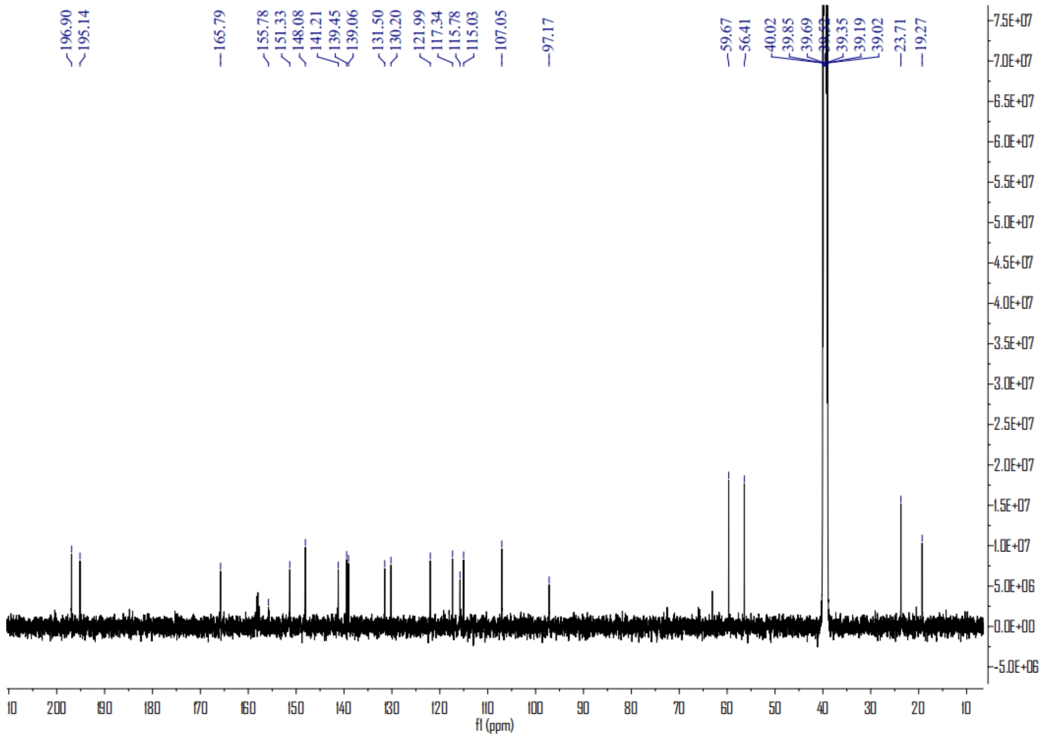

Figure S59. ^{13}C NMR spectrum of **8** in DMSO-d_6

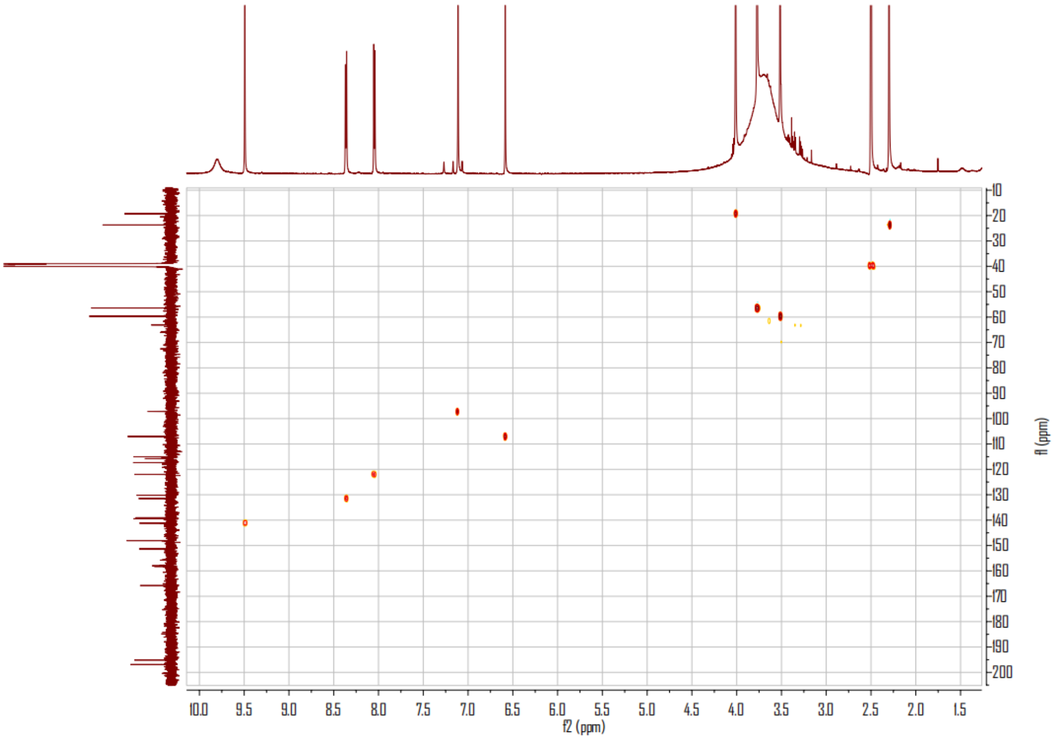

Figure S60. HSQC spectrum of **8** in DMSO-d_6

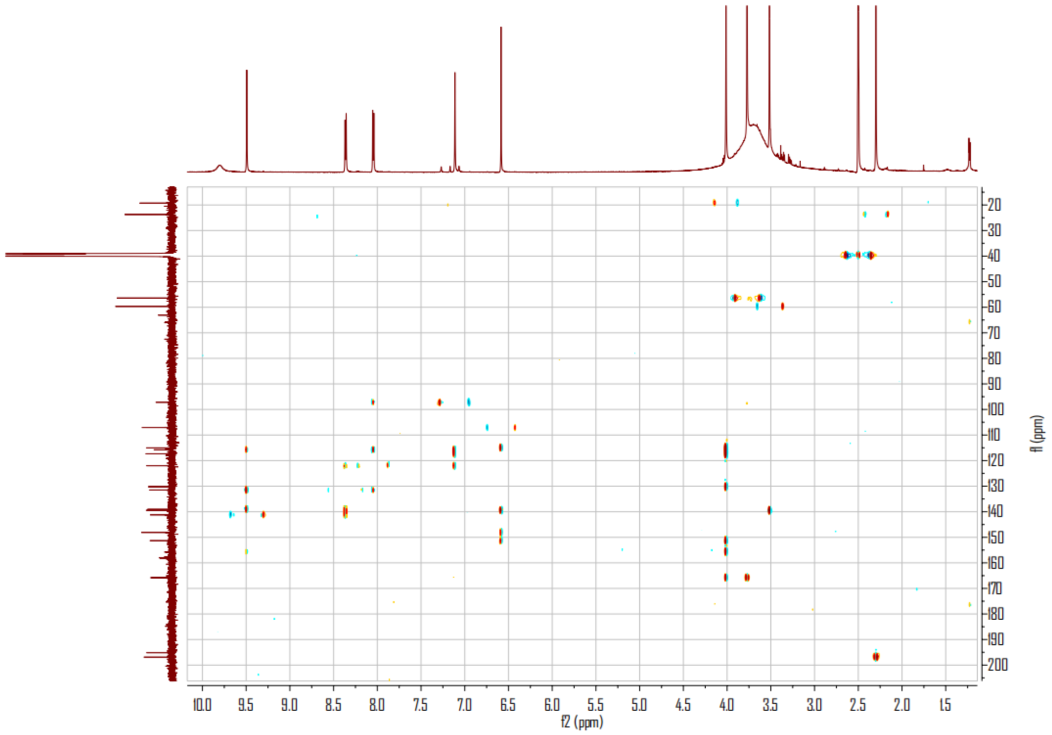

Figure S61. HMBC spectrum of **8** in DMSO-d_6

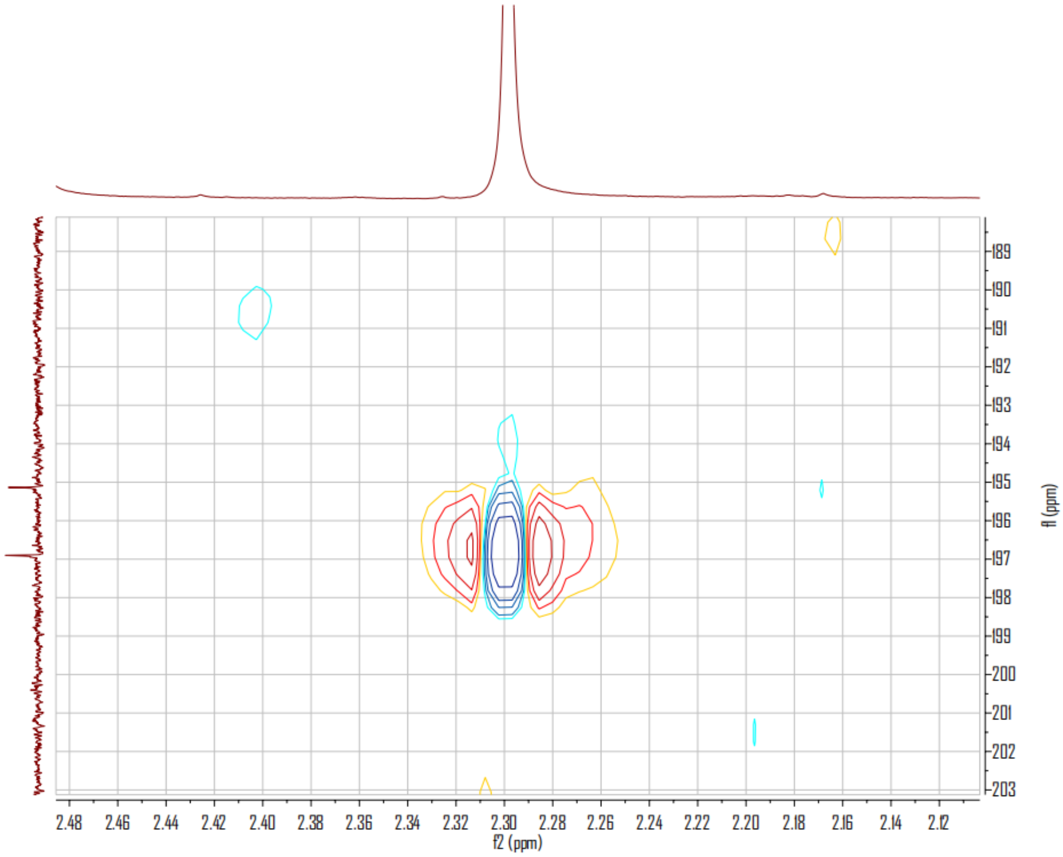

Figure S62. Partial HMBC spectrum of **8** in DMSO-*d*6

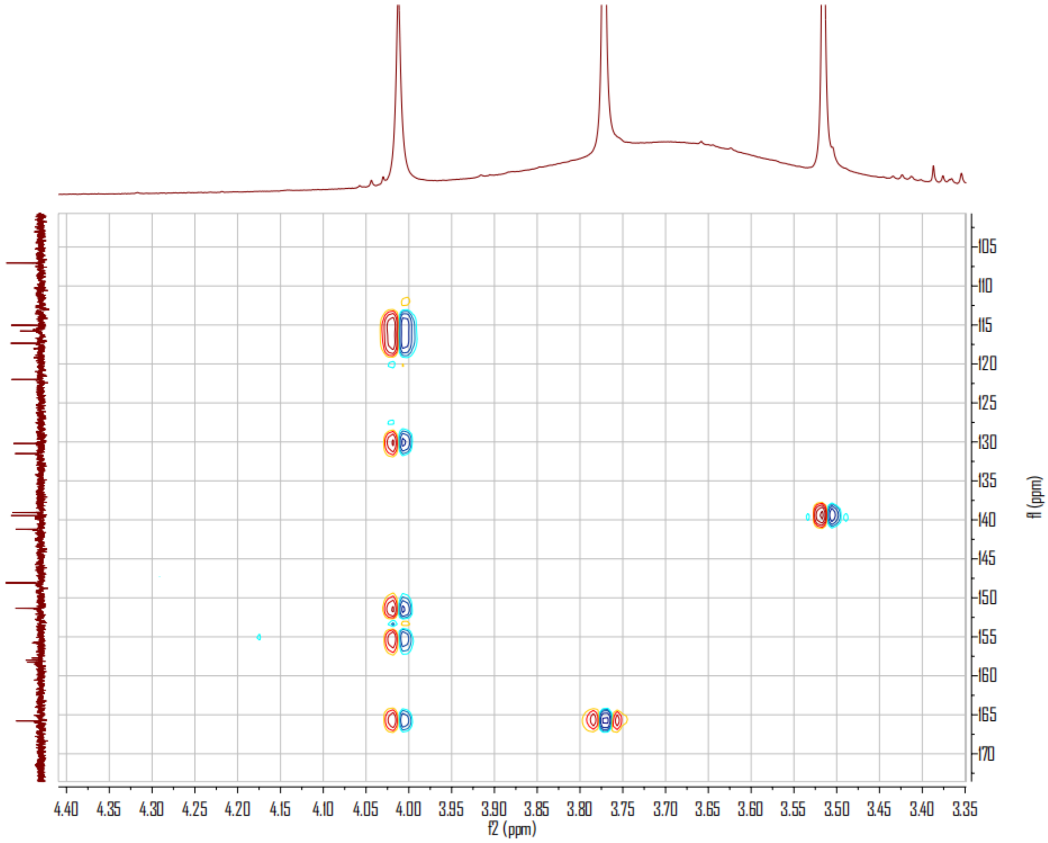

Figure S63. Partial HMBC spectrum of **8** in DMSO-*d*6

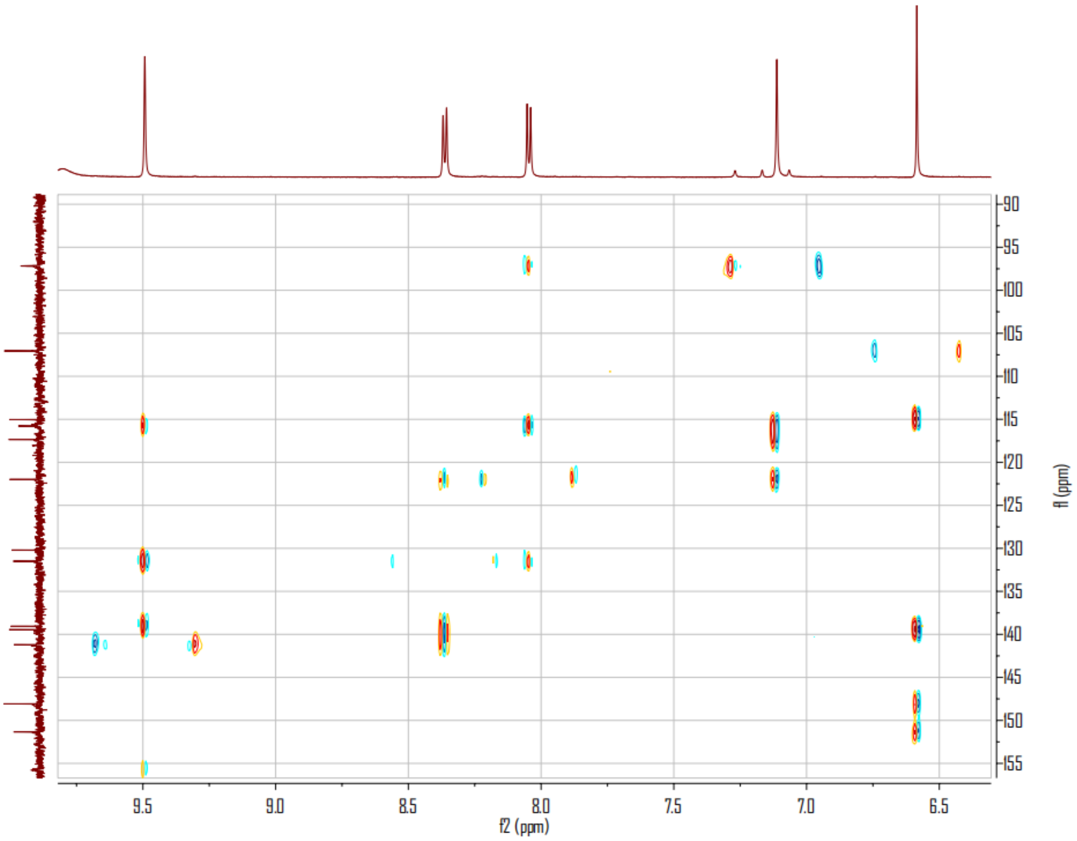

Figure S64. Partial HMBC spectrum of **8** in DMSO-d_6

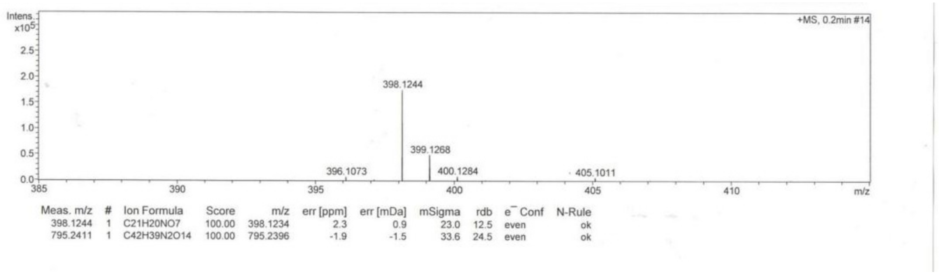

Figure S65. HRESIMS spectrum of **8**

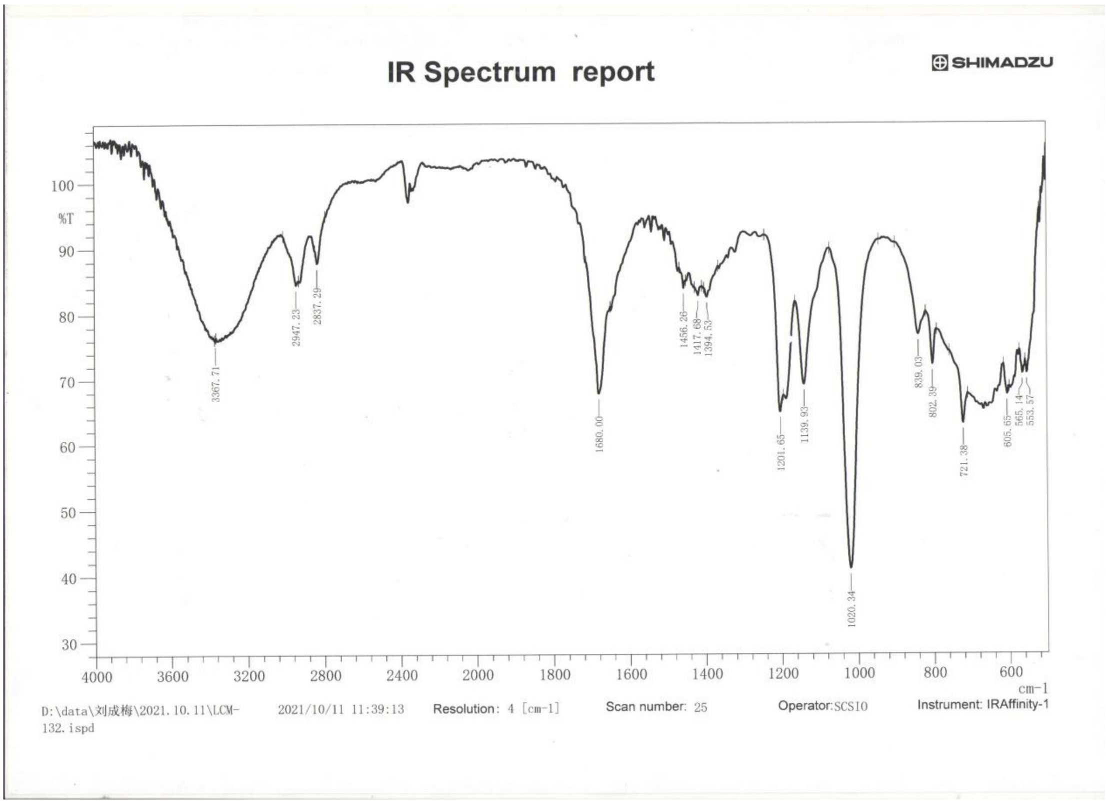

Figure S66. IR spectrum of **8**

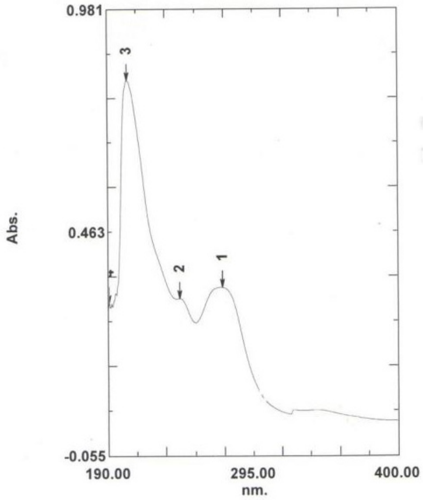

Figure S67. UV spectrum of **8**

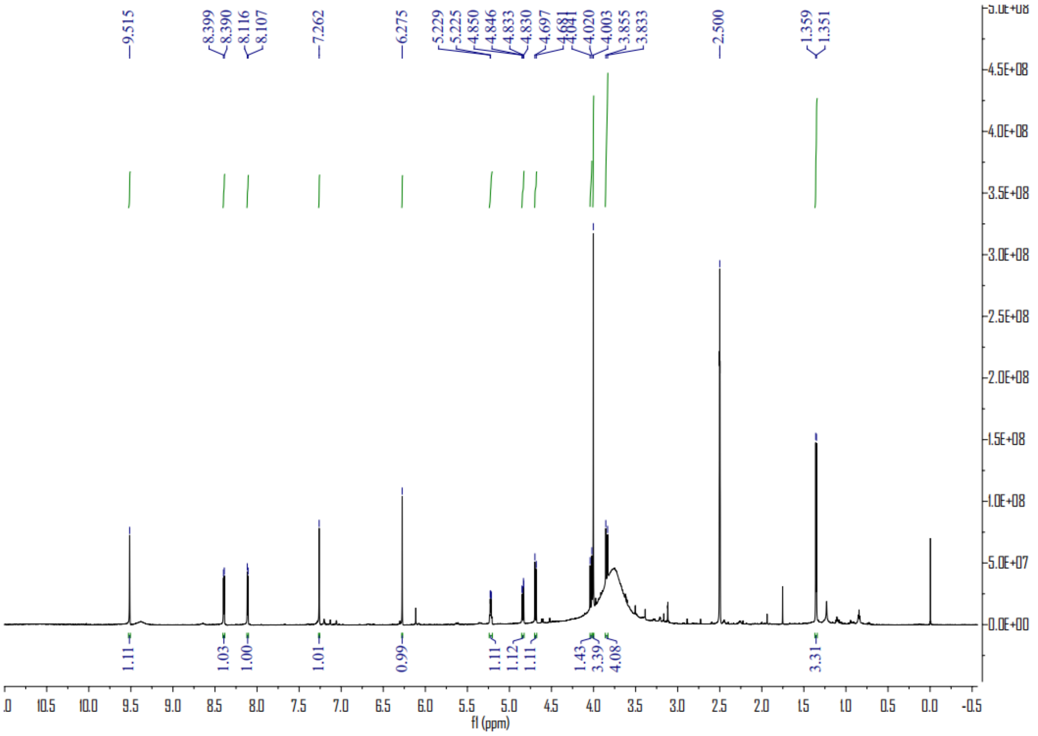

Figure S68. ^{1}H NMR spectrum of **9** in DMSO-d_6

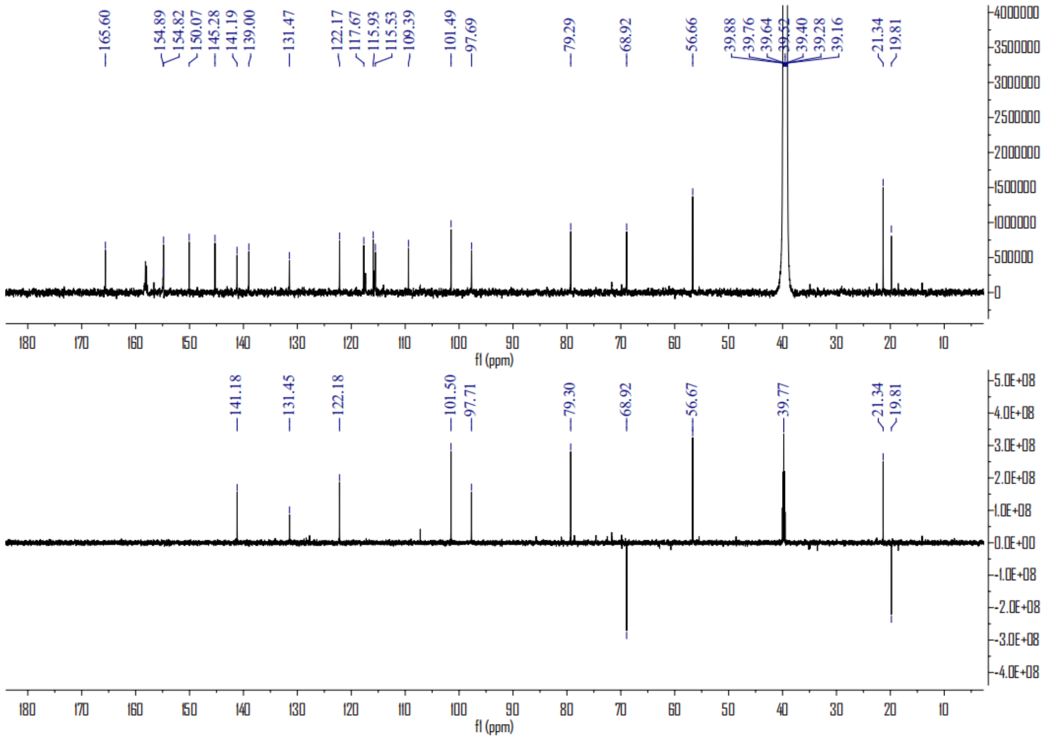

Figure S69. ^{13}C and DEPT135 NMR spectra of **9** in DMSO-d_6

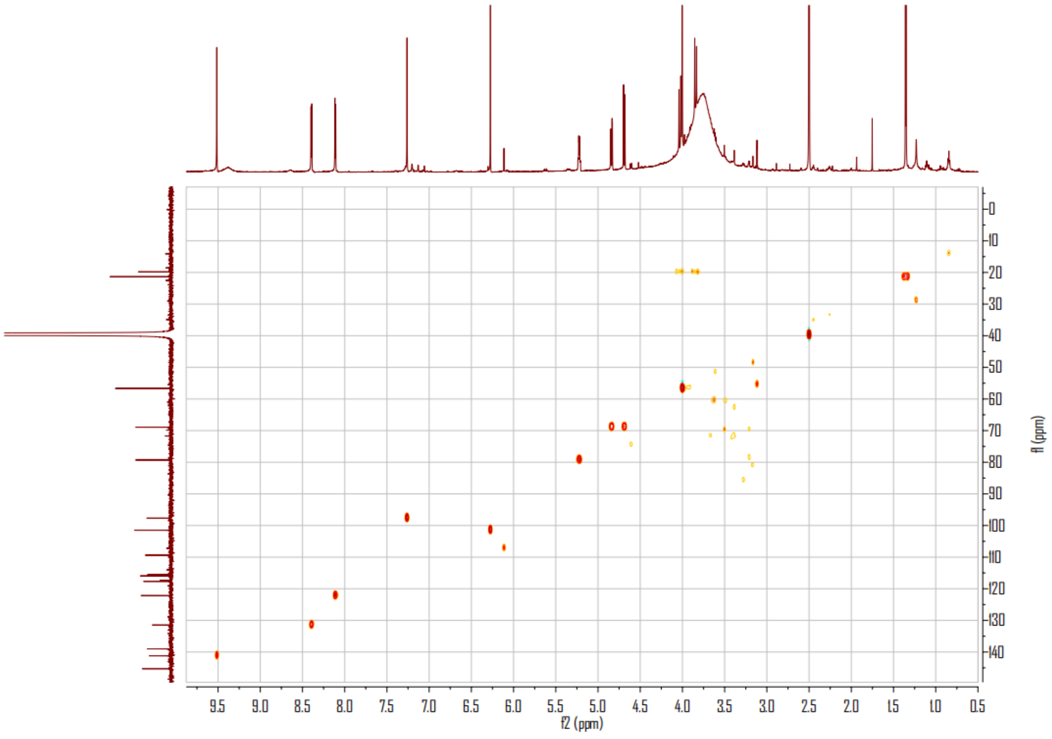

Figure S70. HSQC spectrum of **9** in DMSO-*d*6

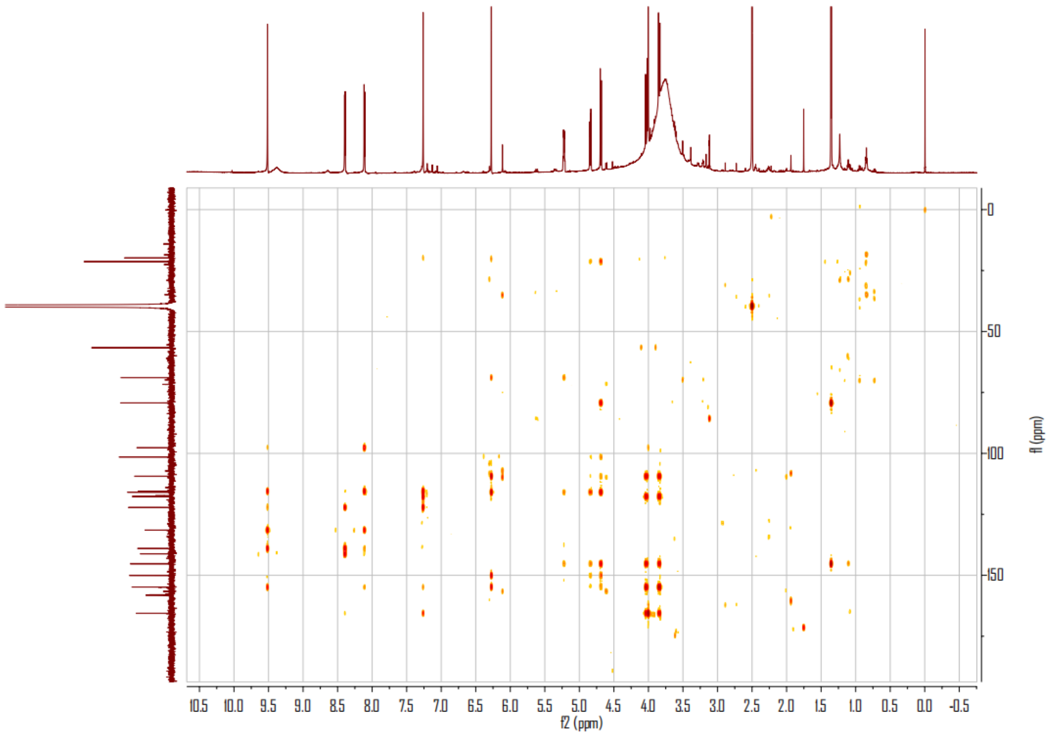

Figure S71. HMBC spectrum of **9** in DMSO-*d*6

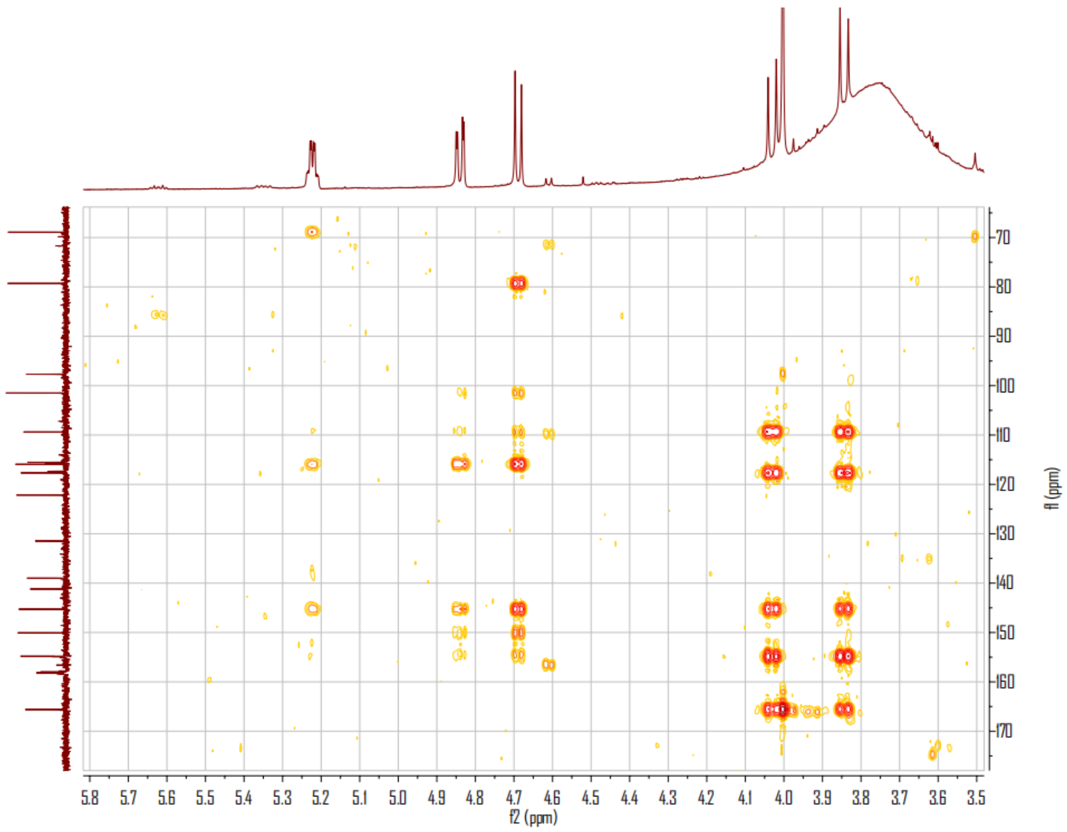

Figure S72. Partial HMBC spectrum of **9** in DMSO-*d*6

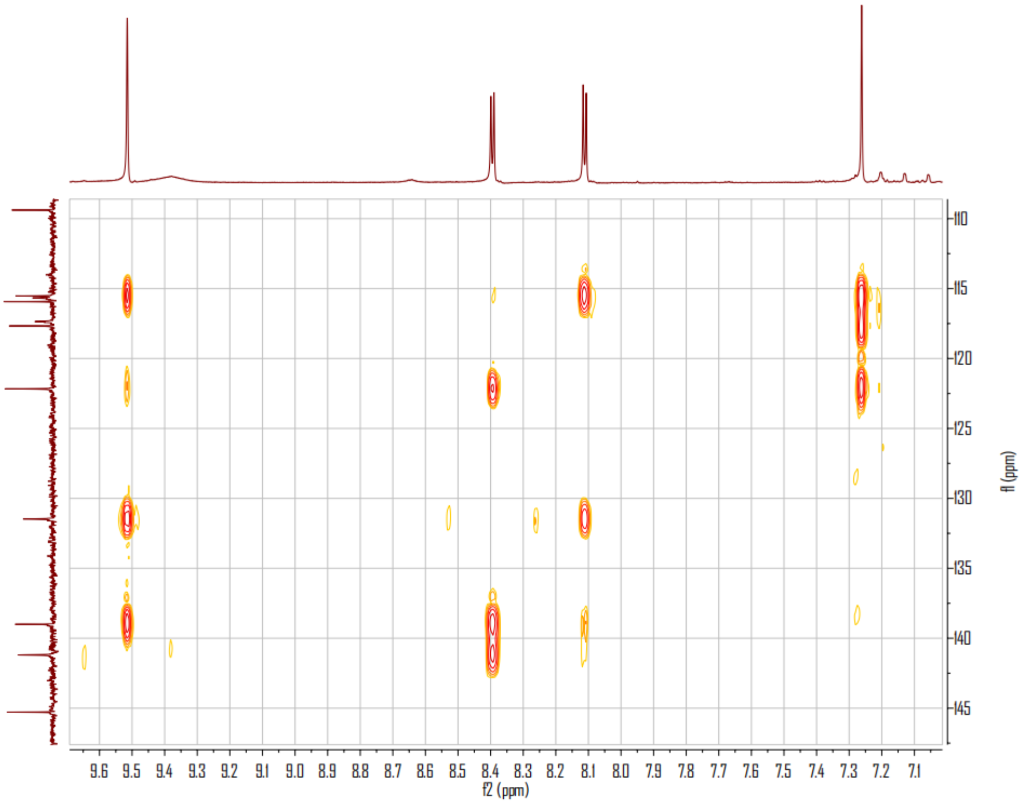

Figure S73. Partial HMBC spectrum of **9** in DMSO-*d*6

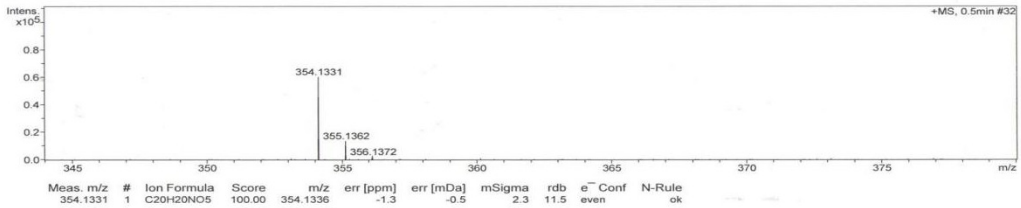

Figure S74. HRESIMS spectrum of **9**

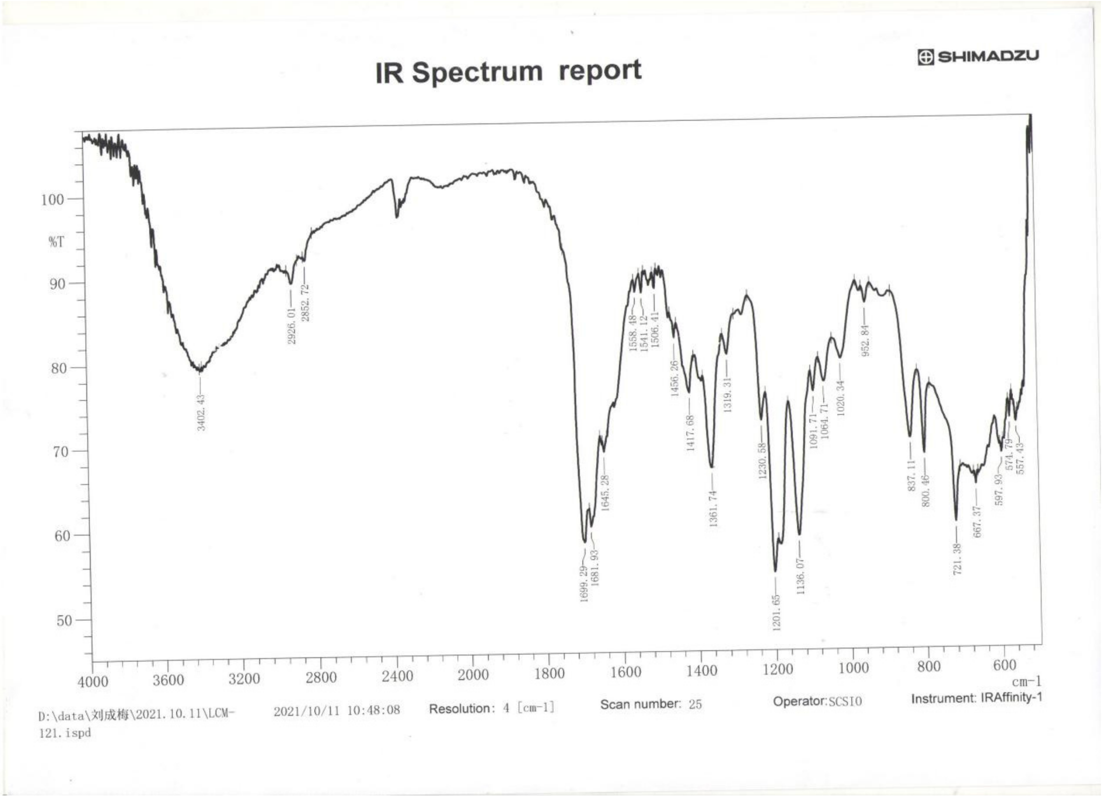

Figure S75. IR spectrum of **9**

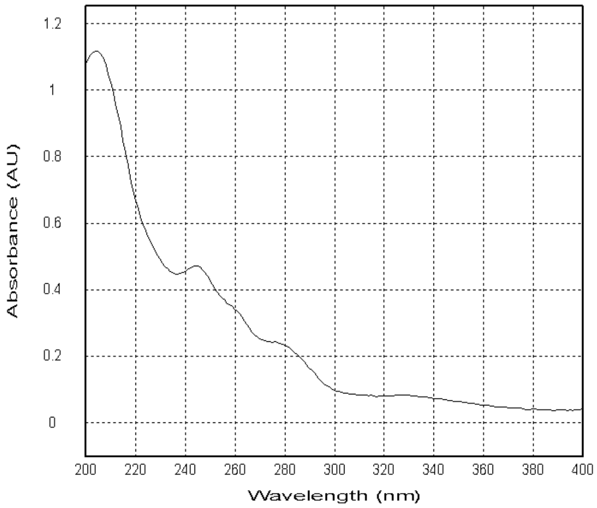

Figure S76. UV spectrum of **9**

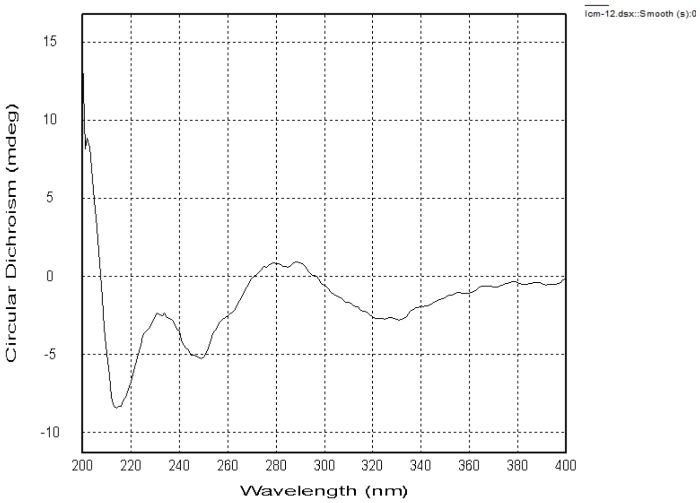

Figure S77. CD spectrum of **9** in methanol

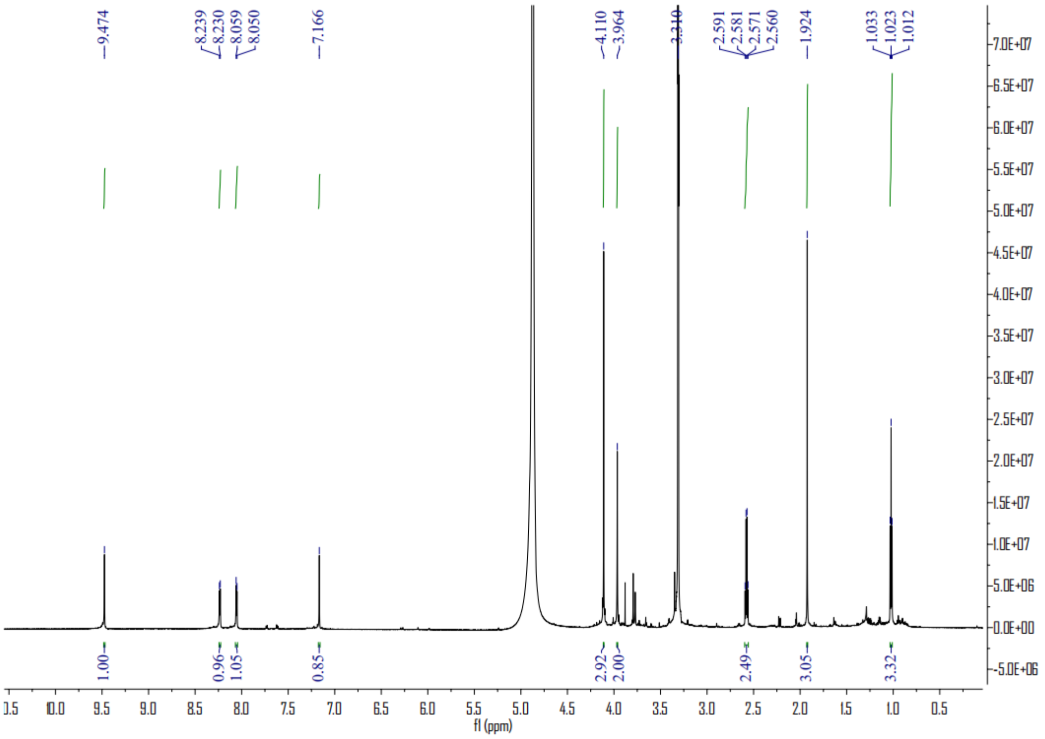

Figure S78. ^{1}H NMR spectrum of **10** in methanol-d_4

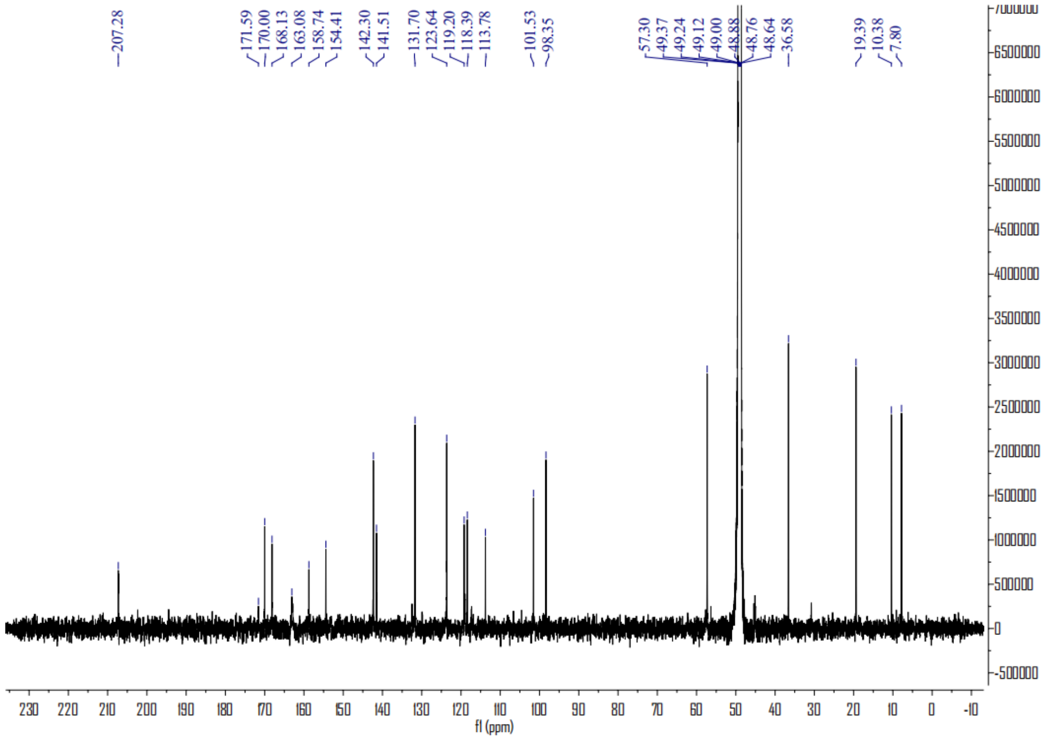

Figure S79. ^{13}C NMR spectrum of **10** in methanol-d_4

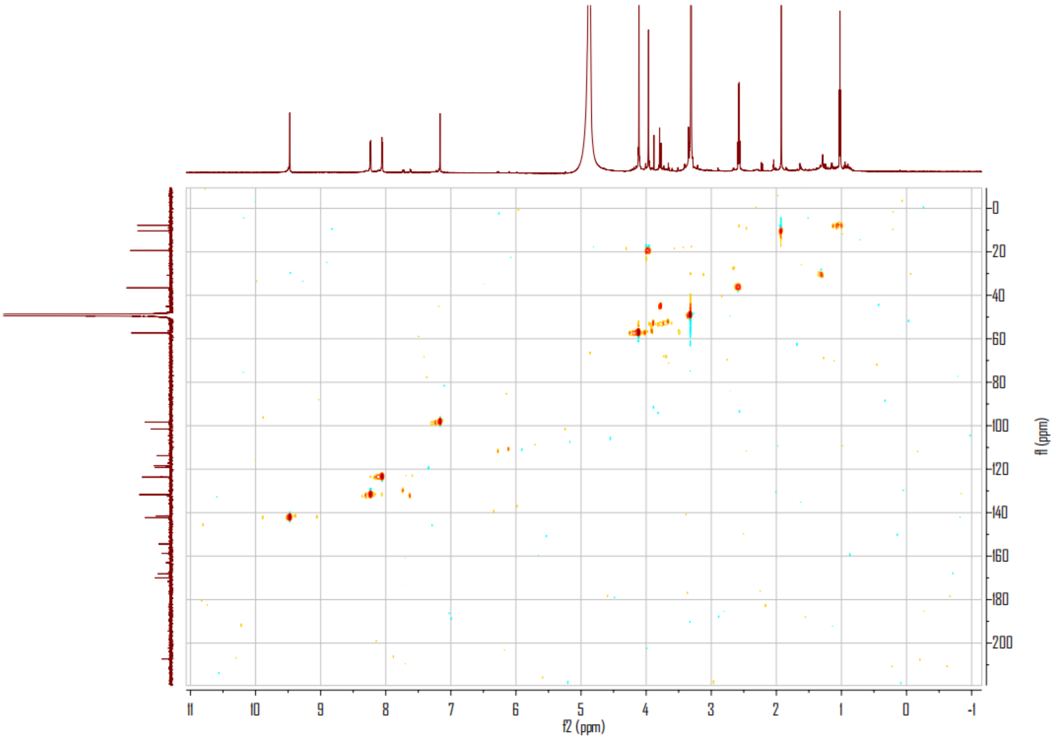

Figure S80. HSQC spectrum of **10** in methanol-d_4

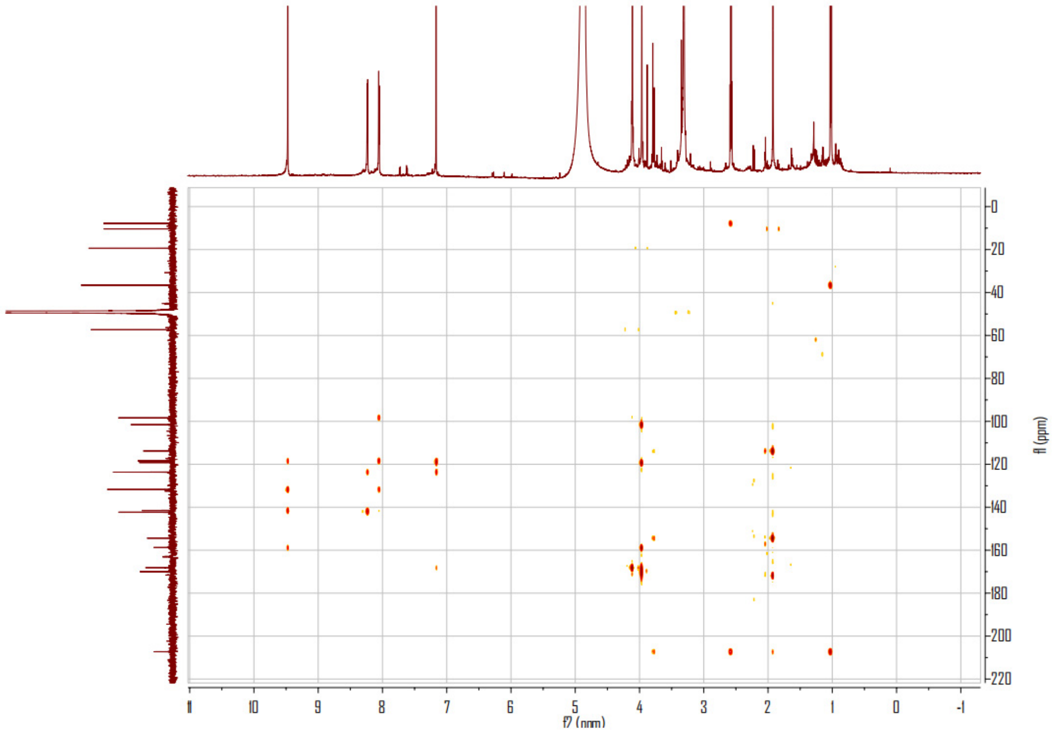

Figure S81. HMBC spectrum of **10** in methanol-d_4

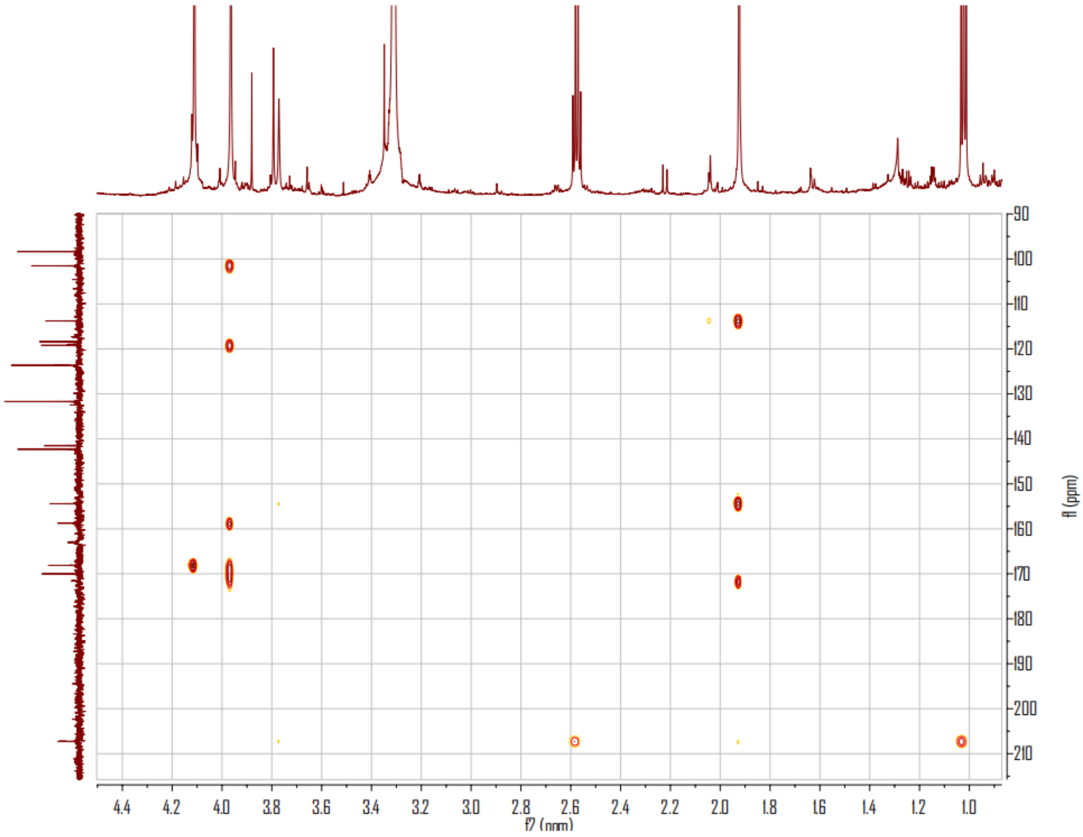

Figure S82. Partial HMBC spectrum of **10** in methanol-d_4

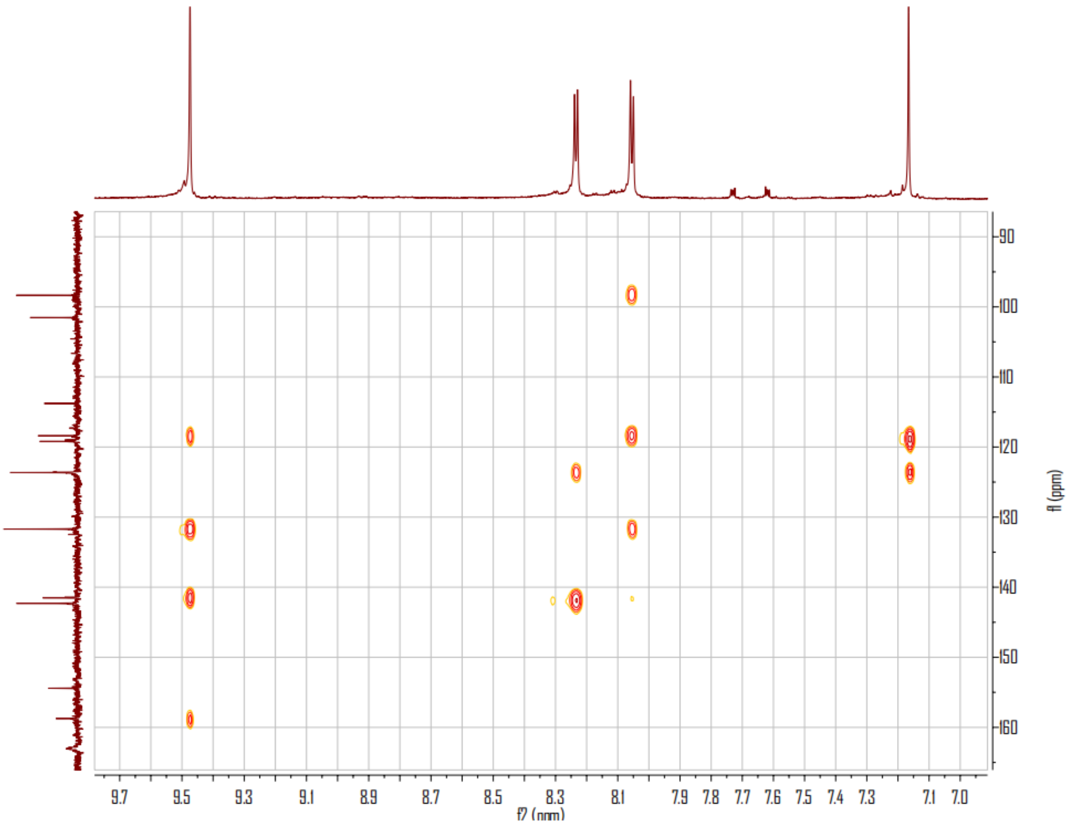

Figure S83. Partial HMBC spectrum of **10** in methanol-d_4

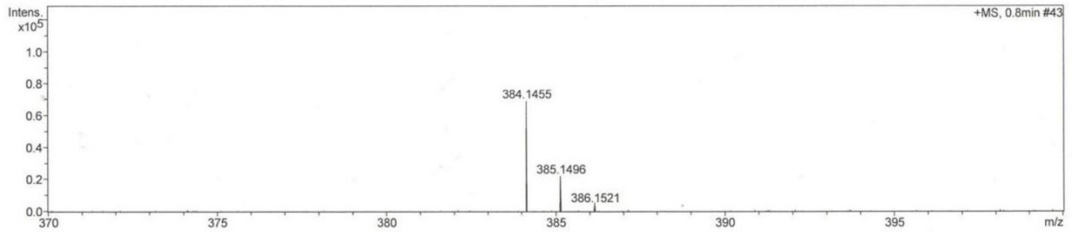

Meas. m/z	#	Ion Formula	Score	m/z	err [ppm]	err [mDa]	mSigma	rdb	e⁻ Conf	N-Rule
384.1455	1	C21H22NO6	100.00	384.1442	3.6	1.4	43.0	11.5	even	ok

Figure S84. HRESIMS spectrum of **10**

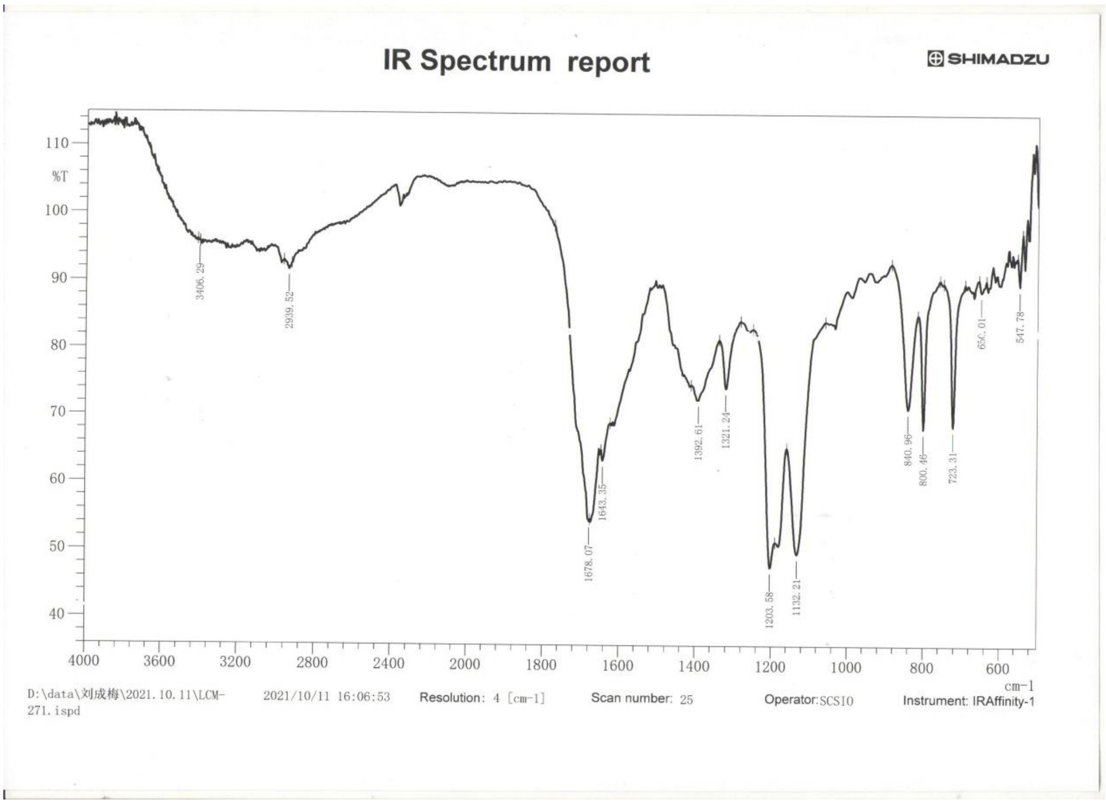

Figure S85. IR spectrum of **10**

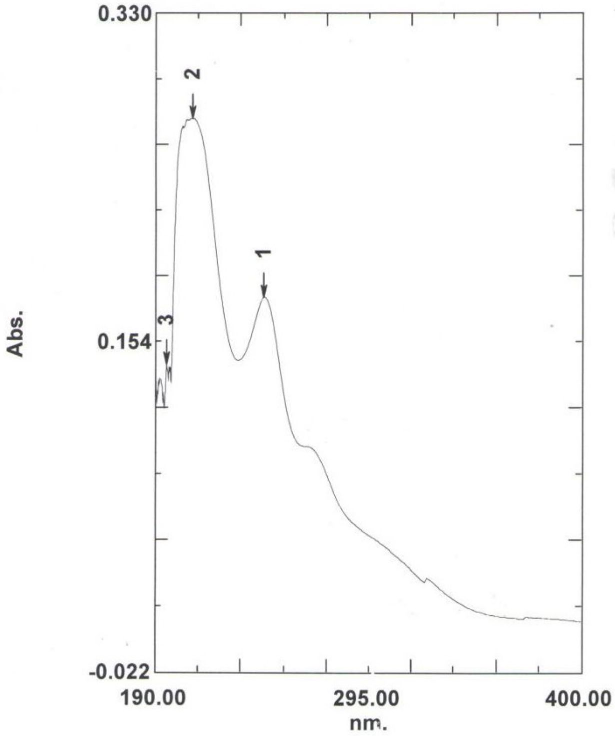

Figure S86. UV spectrum of **10**

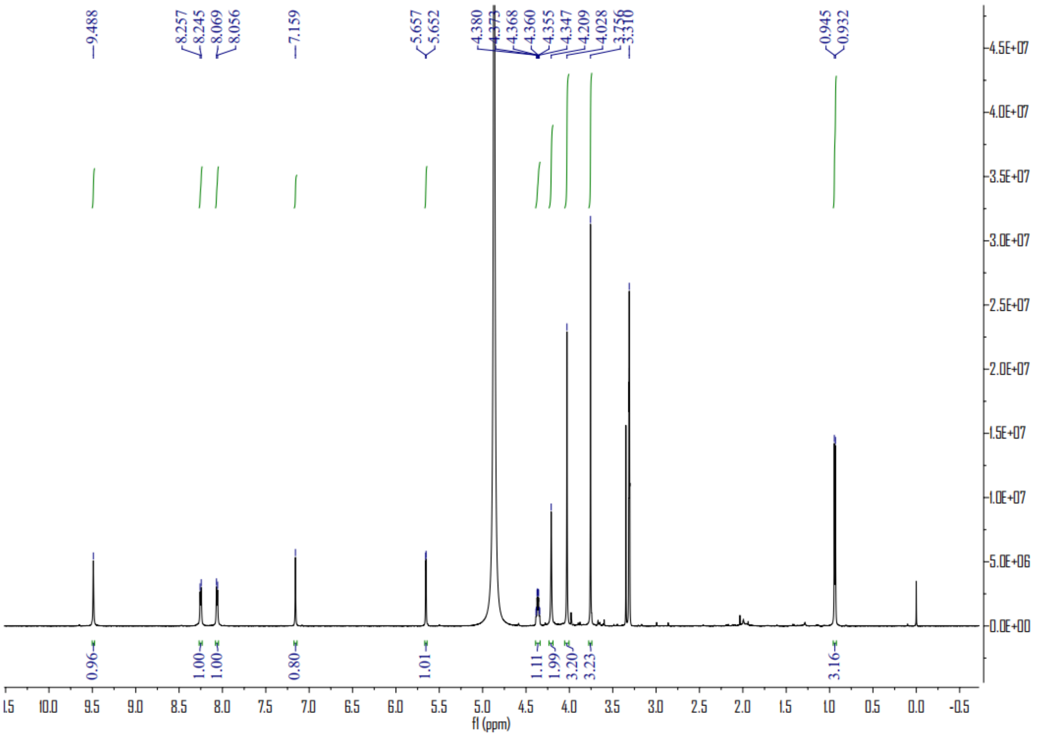

Figure S87. ^{1}H NMR spectrum of **11** in methanol-d_4

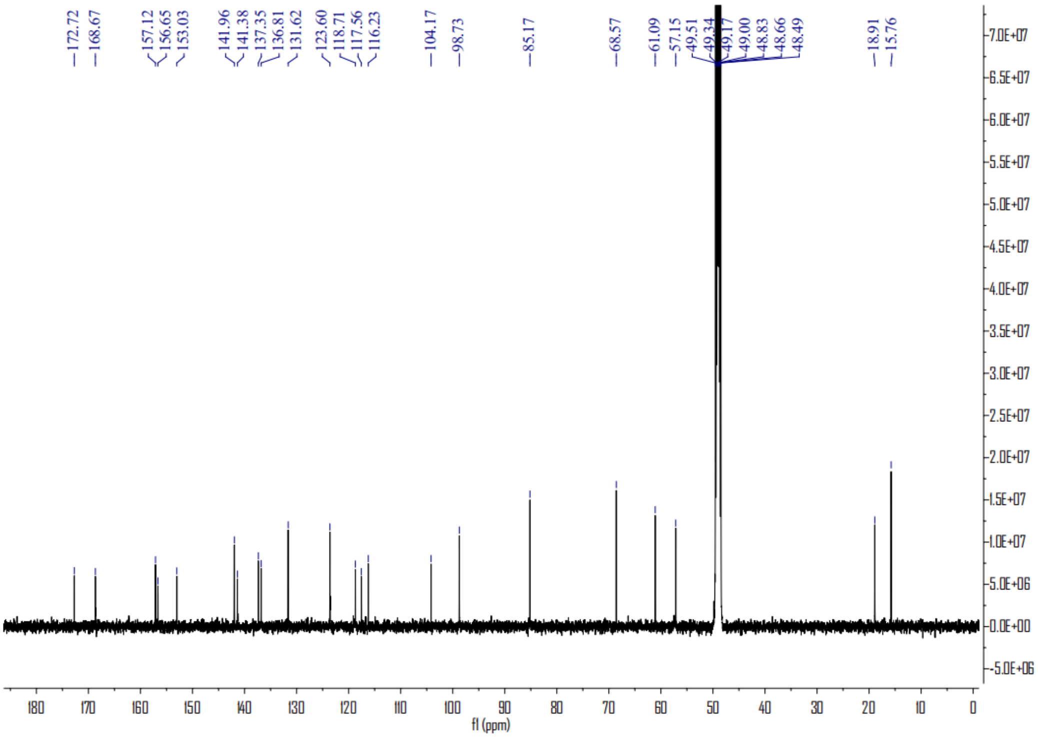

Figure S88. ^{13}C NMR spectrum of **11** in methanol-d_4

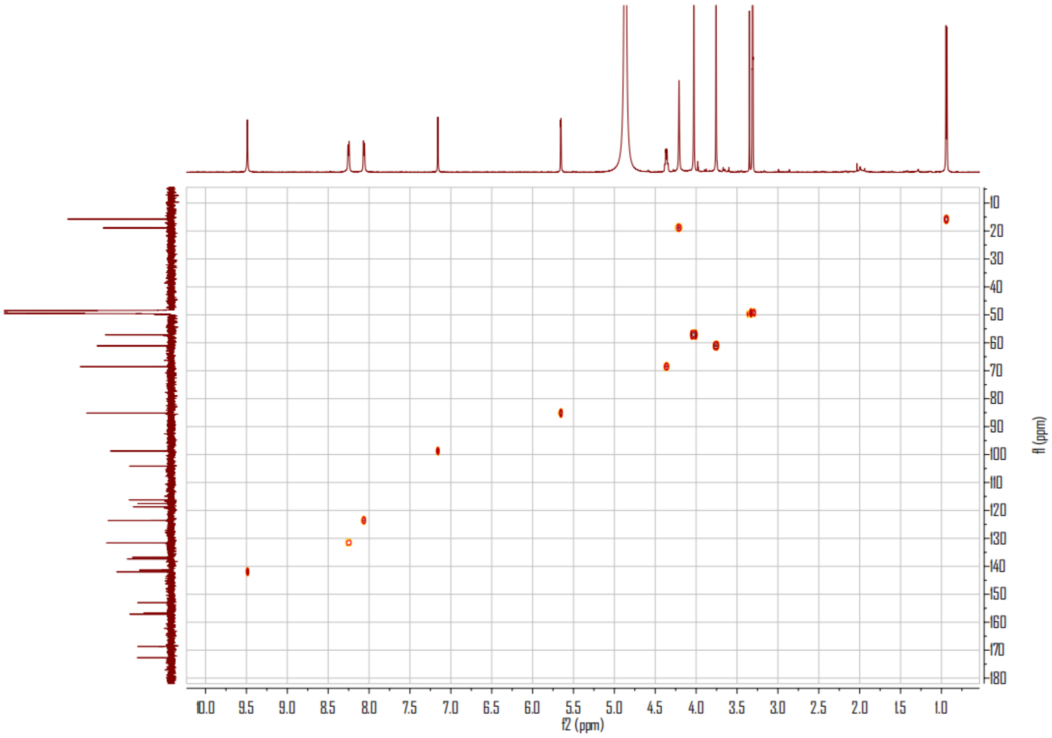

Figure S89. HSQC spectrum of **11** in methanol-*d*4

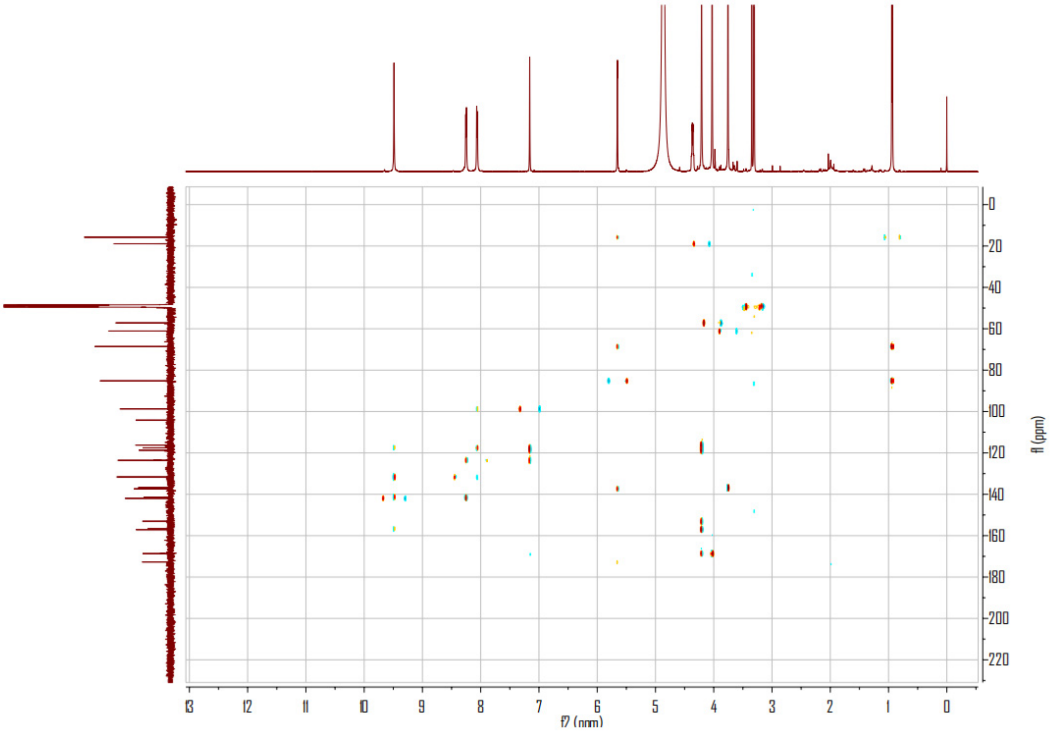

Figure S90. HMBC spectrum of **11** in methanol-*d*4

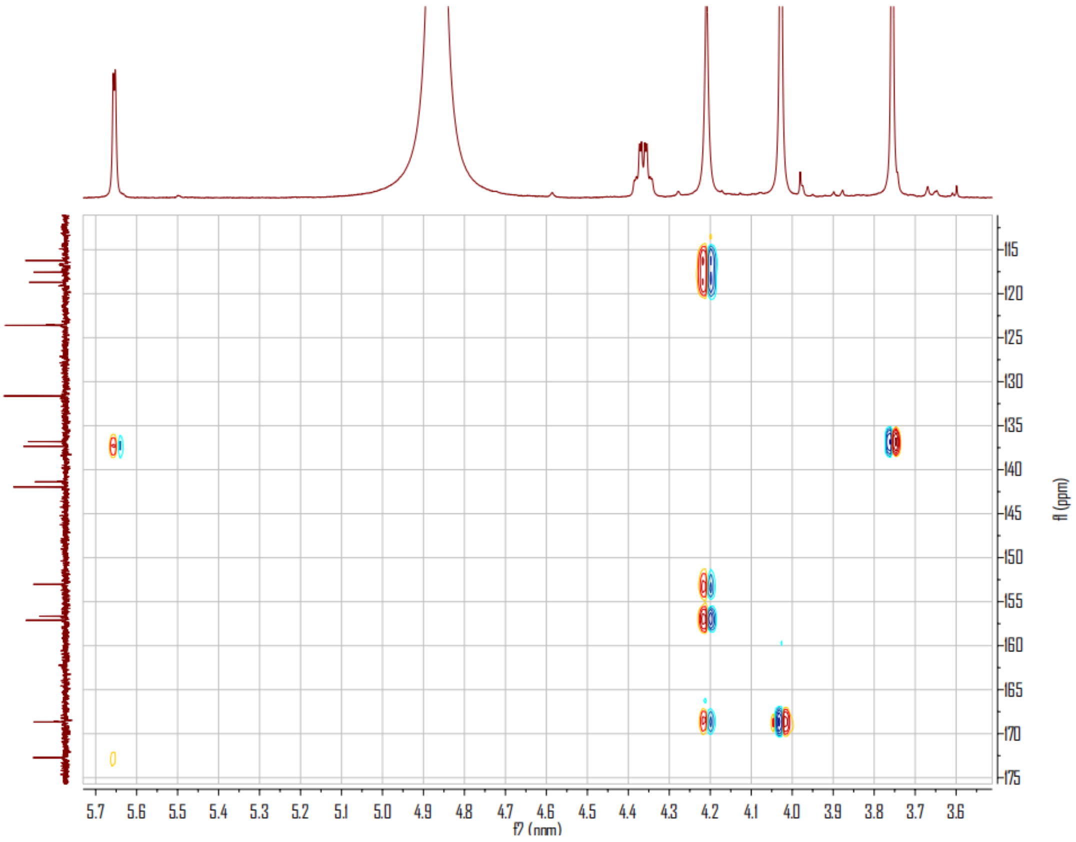

Figure S91. Partial HMBC spectrum of **11** in methanol-d_4

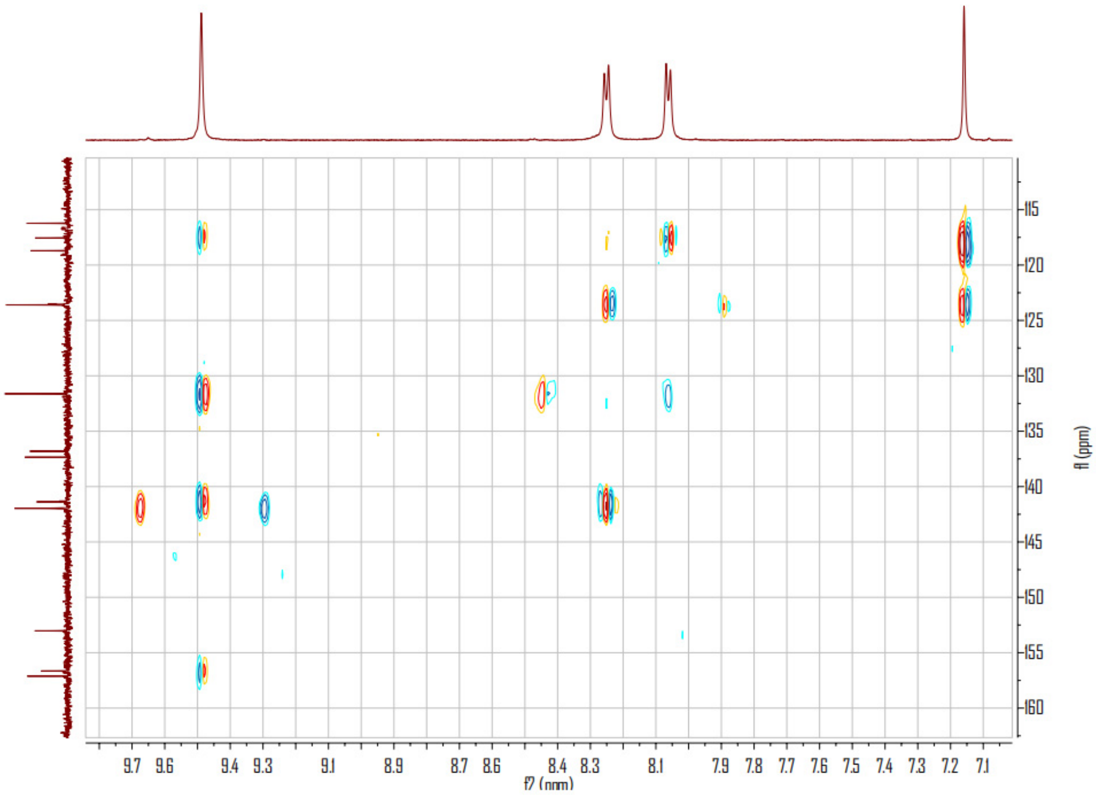

Figure S92. Partial HMBC spectrum of **11** in methanol-d_4

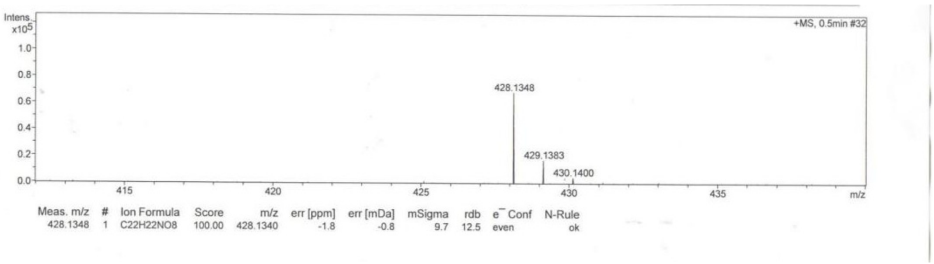

Figure S93. HRESIMS spectrum of **11**

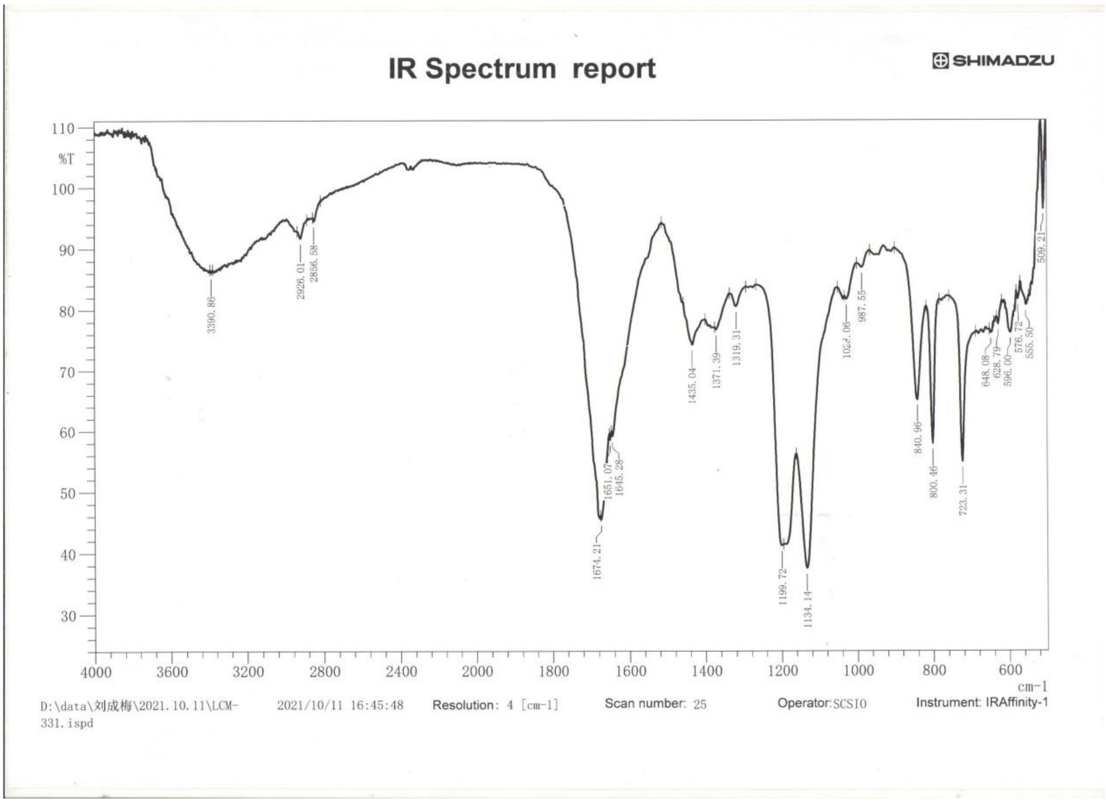

Figure S94. IR spectrum of **11**

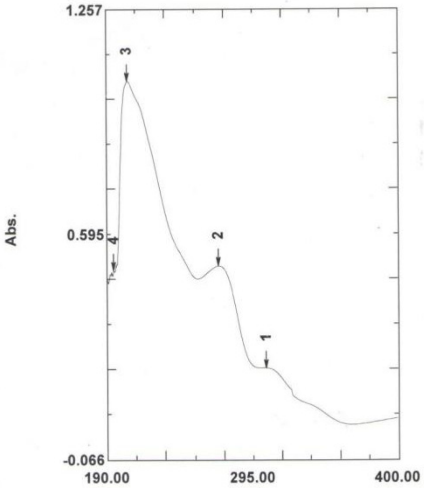

Figure S95. UV spectrum of **11**

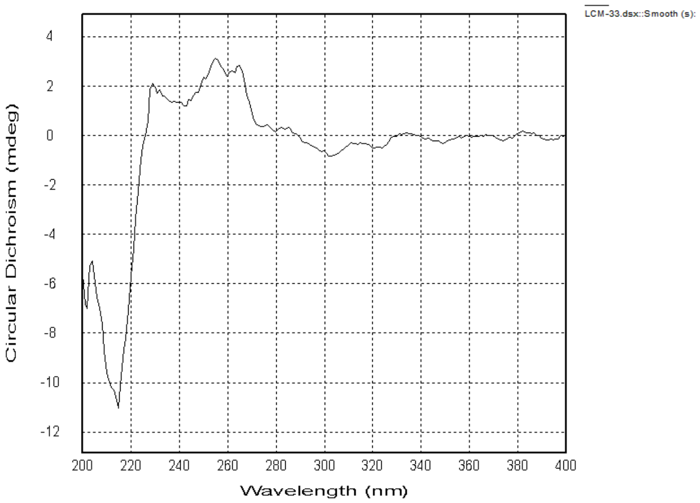

Figure S96. CD spectrum of **11** in methanol

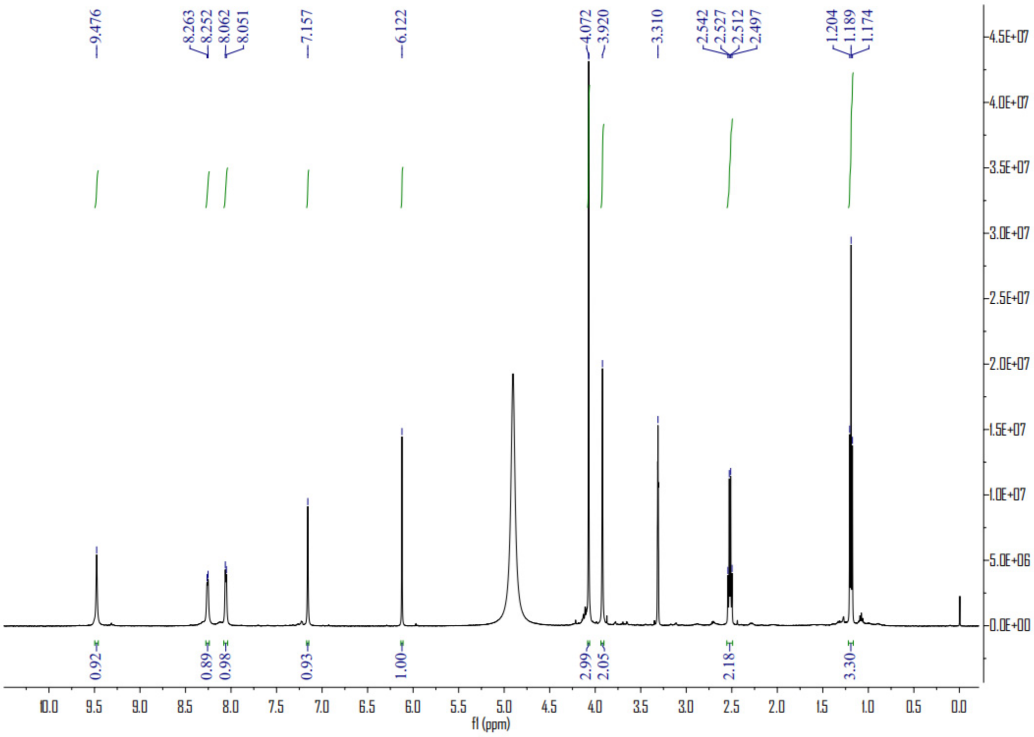

Figure S97. ^{1}H NMR spectrum of **12** in methanol-d_4

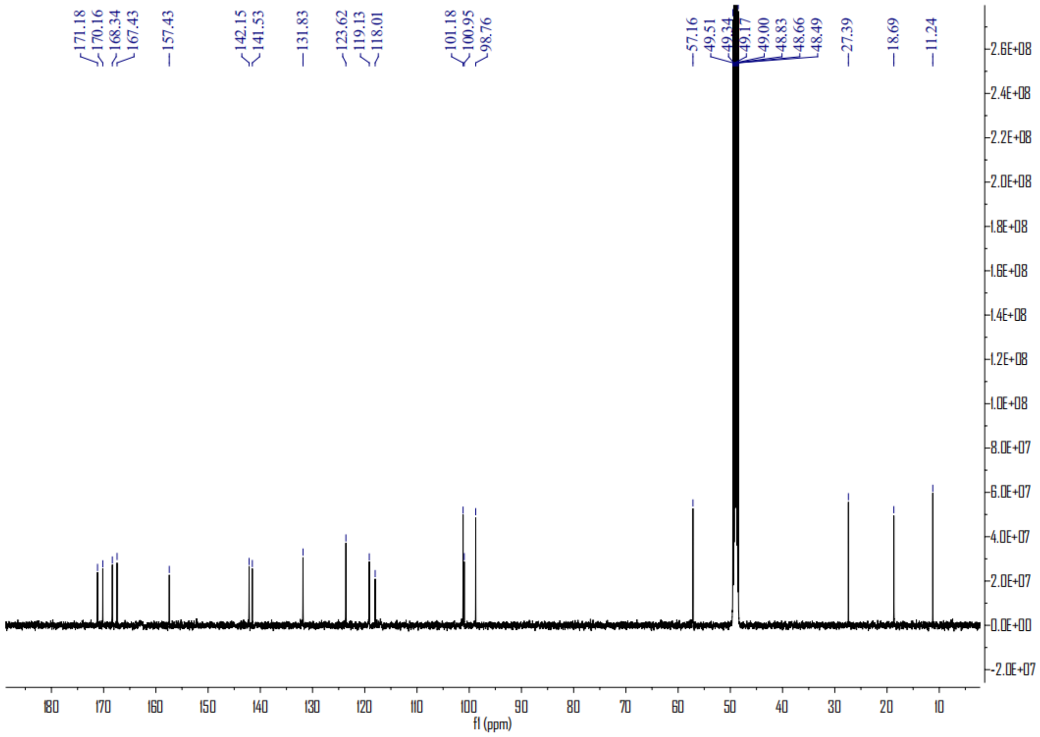

Figure S98. ^{13}C NMR spectrum of **12** in methanol-*d*4

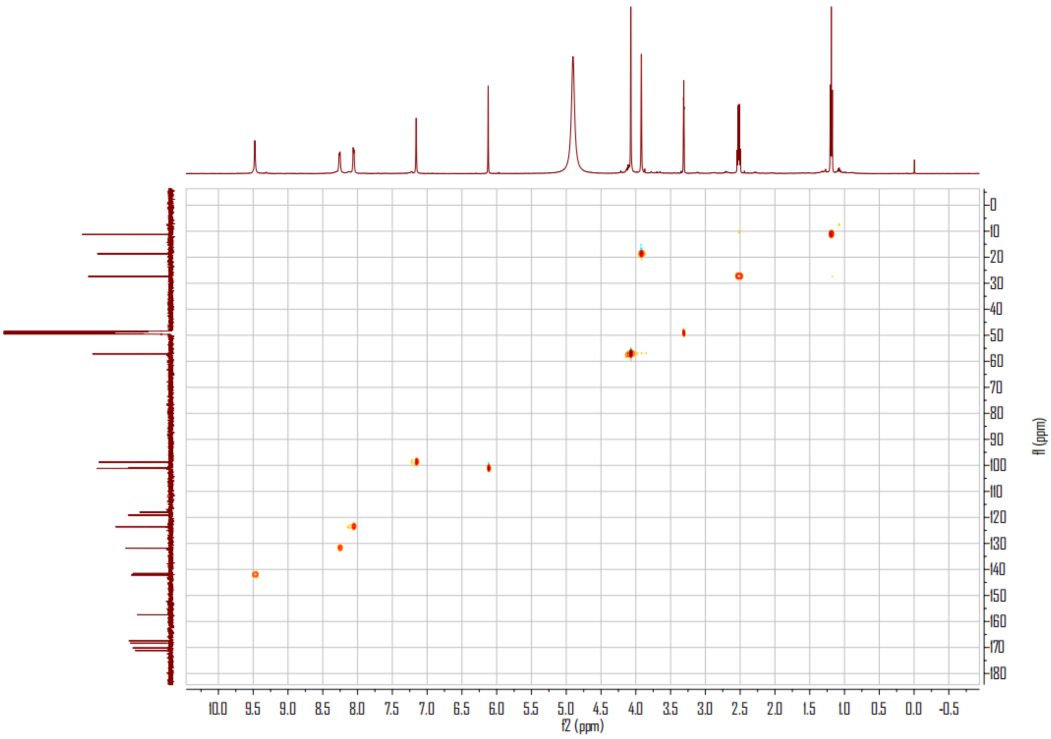

Figure S99. HSQC spectrum of **12** in methanol-*d*4

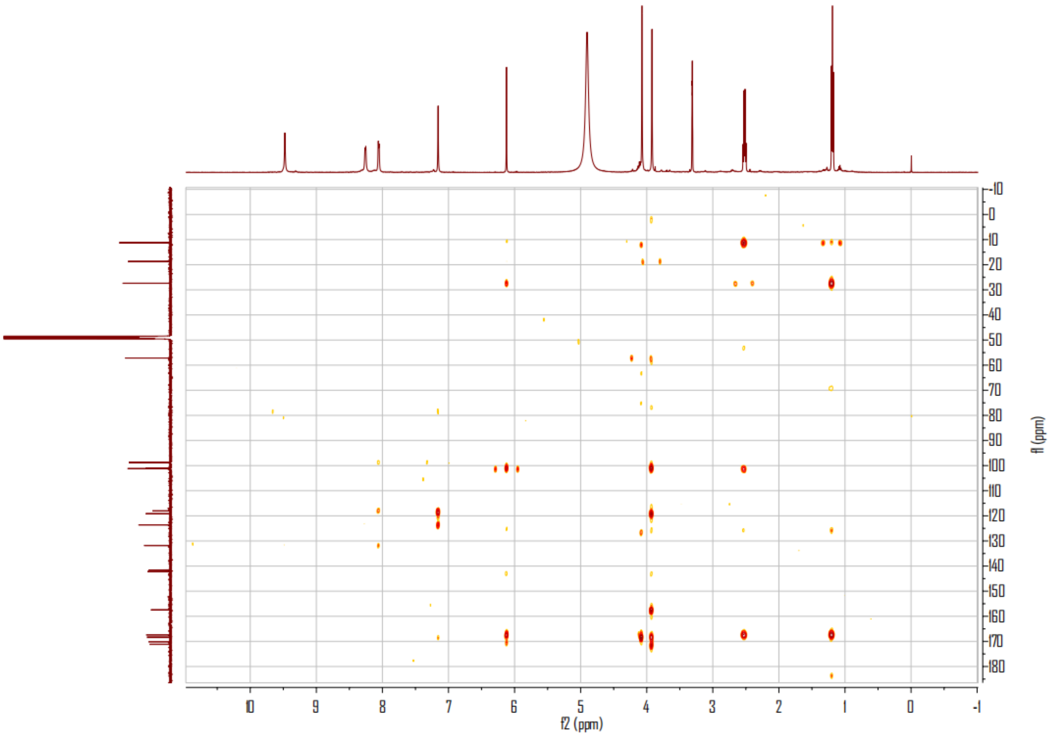

Figure S100. HMBC spectrum of **12** in methanol-*d*4

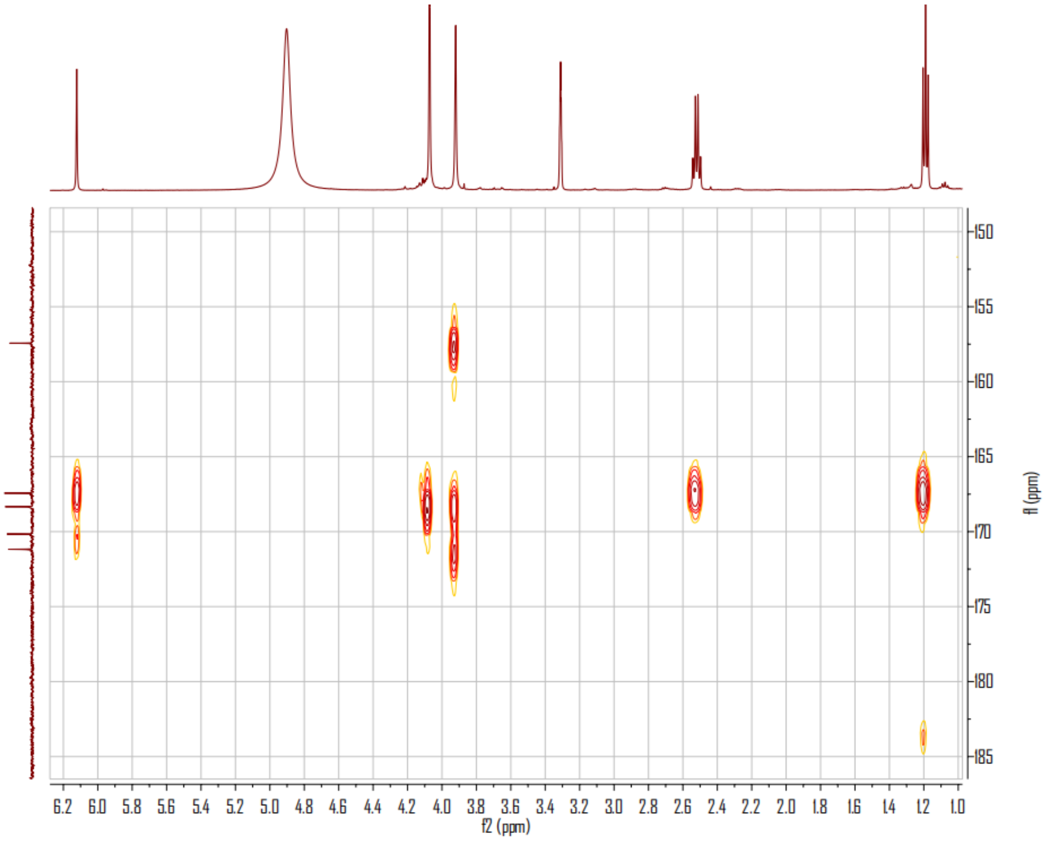

Figure S101. Partial HMBC spectrum of **12** in methanol-*d*4

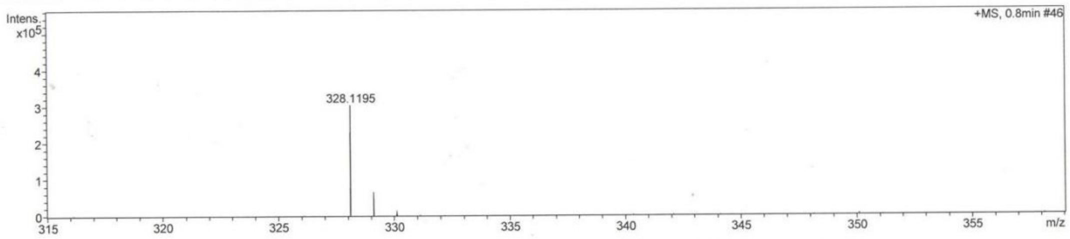

Meas. m/z	#	Ion Formula	Score	m/z	err [ppm]	err [mDa]	mSigma	rdb	e⁻ Conf	N-Rule
328.1195	1	C18H18NO5	100.00	328.1179	-4.8	-1.6	7.4	10.5	even	ok
350.1017	1	C18H17NNaO5	100.00	350.0999	-5.2	-1.8	46.7	10.5	even	ok

Figure S102. HRESIMS spectrum of **12**

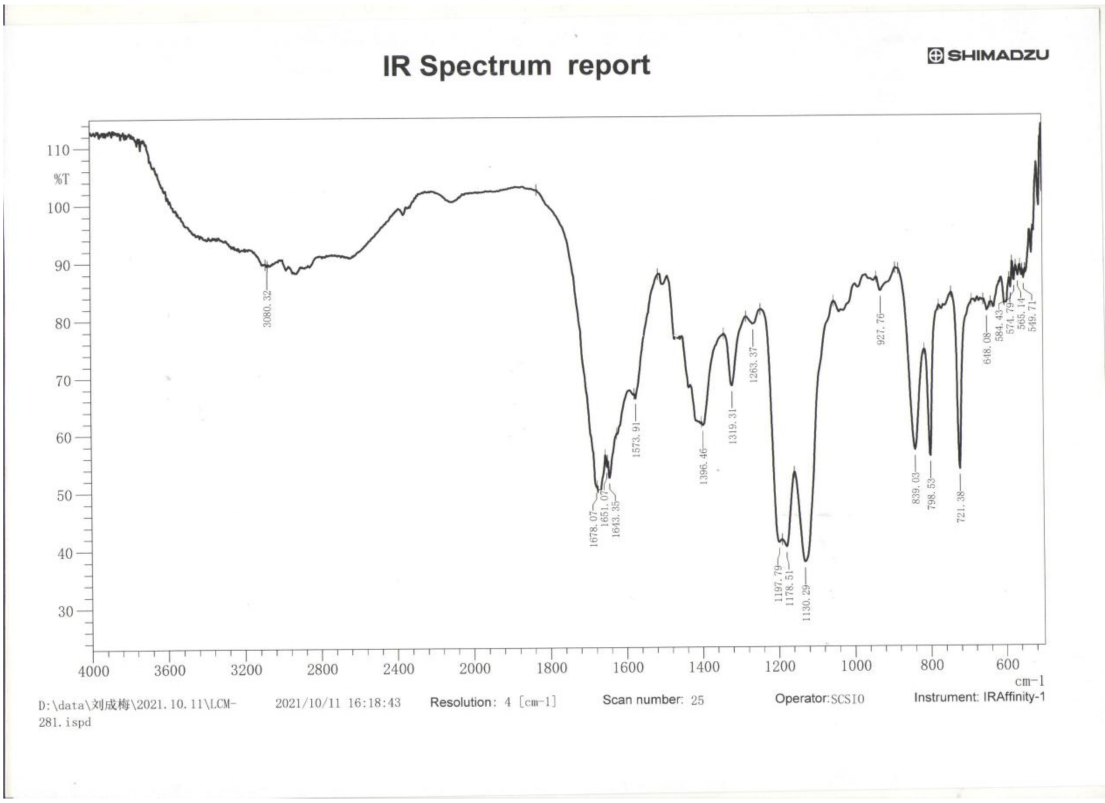

Figure S103. IR spectrum of **12**

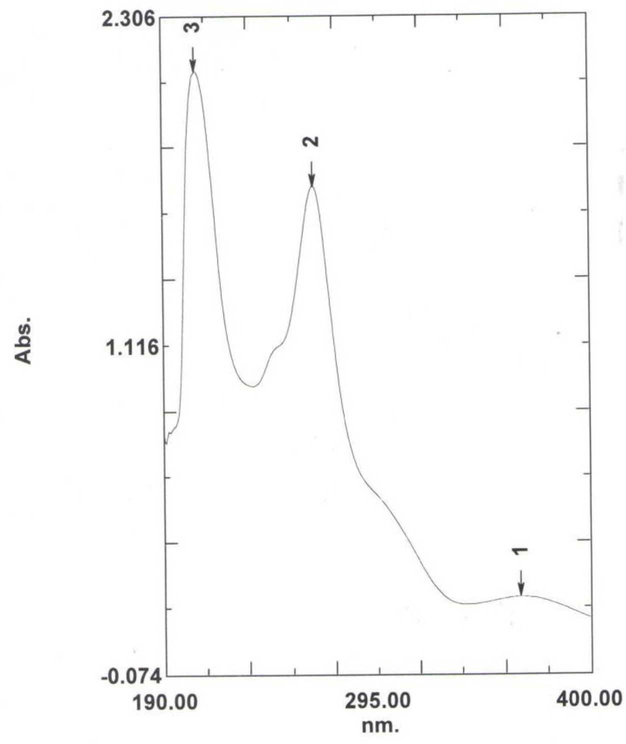

Figure S104. UV spectrum of **12**

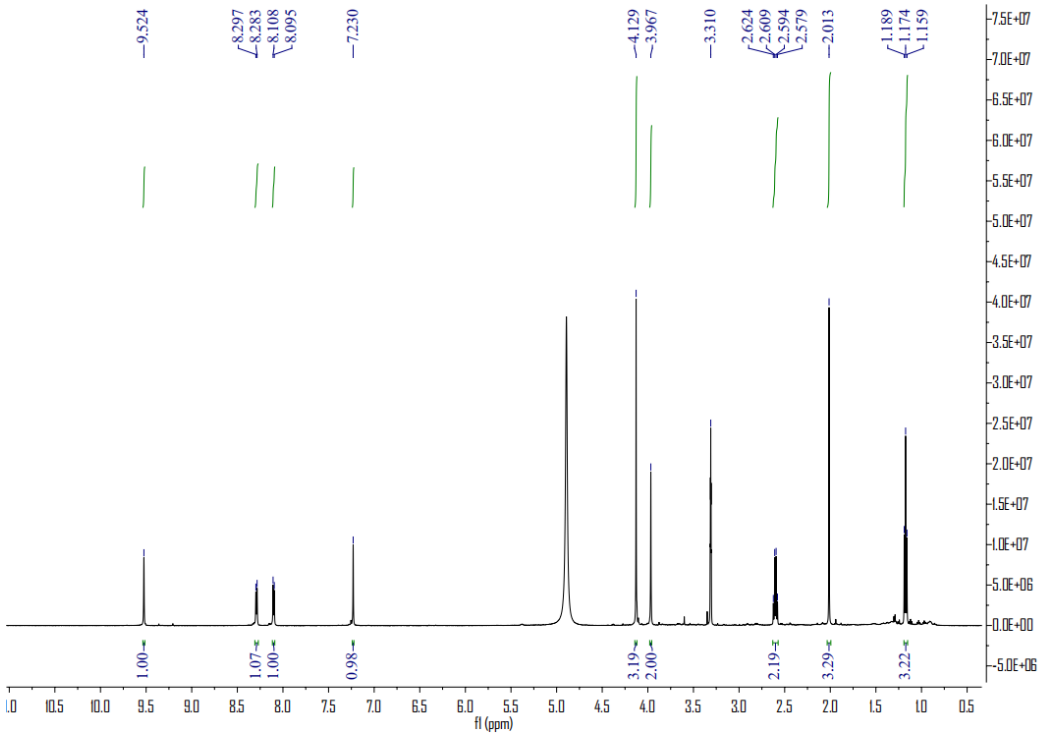

Figure S105. ^{1}H NMR spectrum of **13** in methanol-d_4

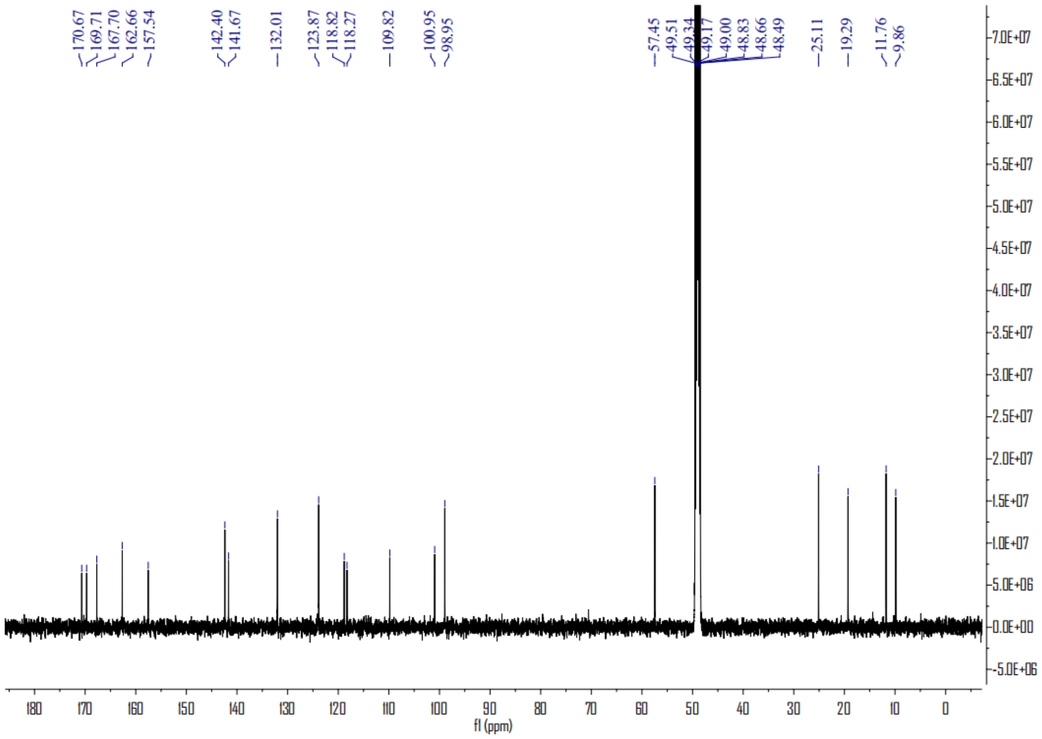

Figure S106. ^{13}C NMR spectrum of **13** in methanol-d_4

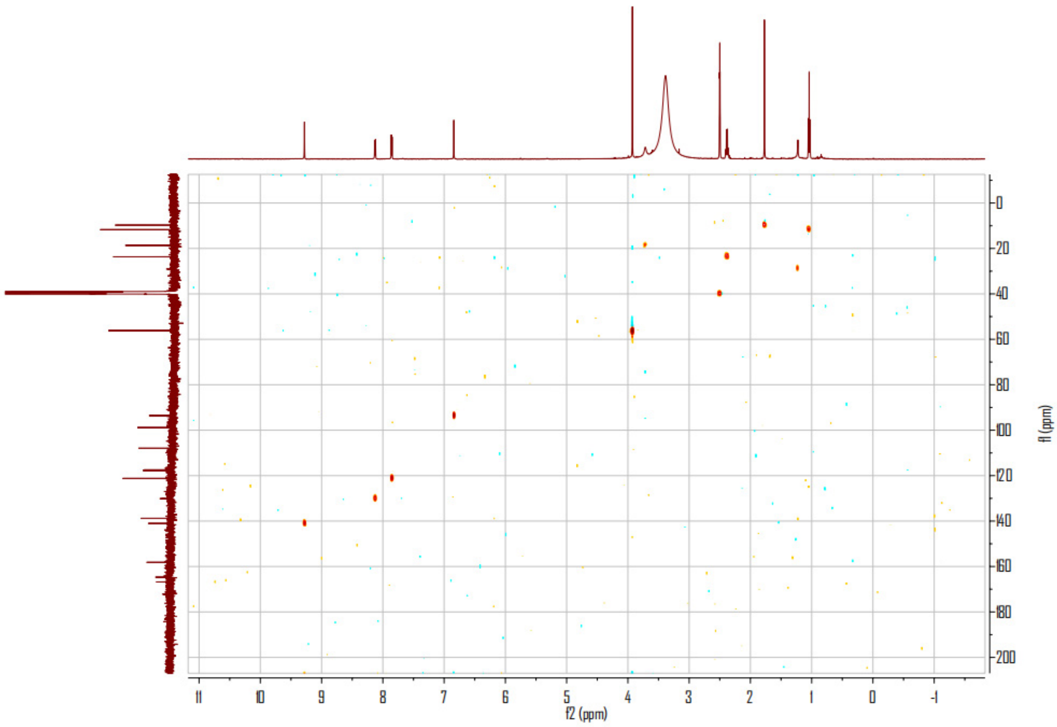

Figure S107. HSQC spectrum of **13** in DMSO-d_6

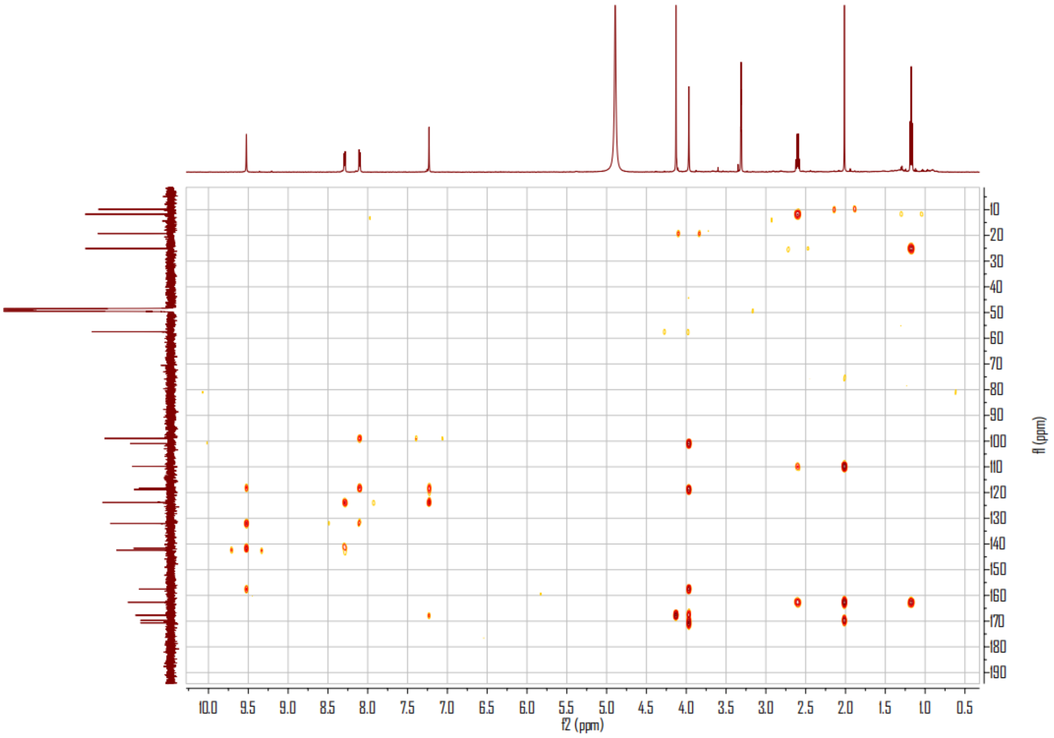

Figure S108. HMBC spectrum of **13** in methanol-d_4

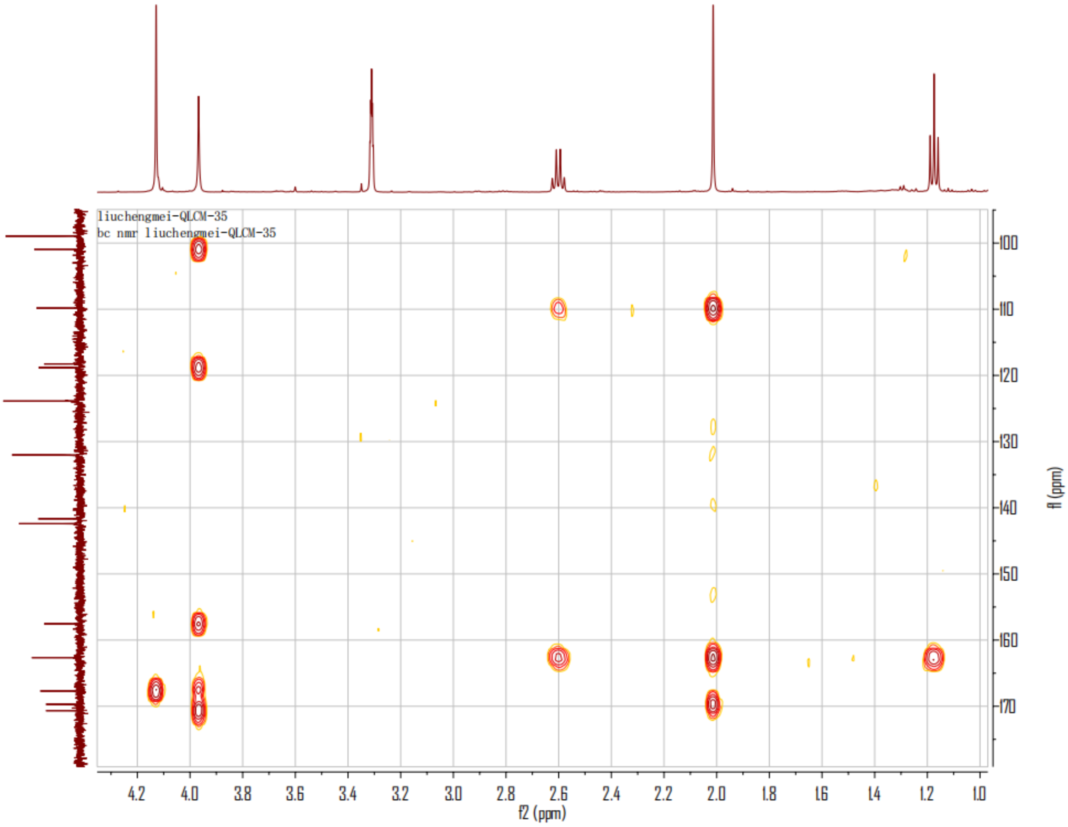

Figure S109. Partial HMBC spectrum of **13** in methanol-d_4

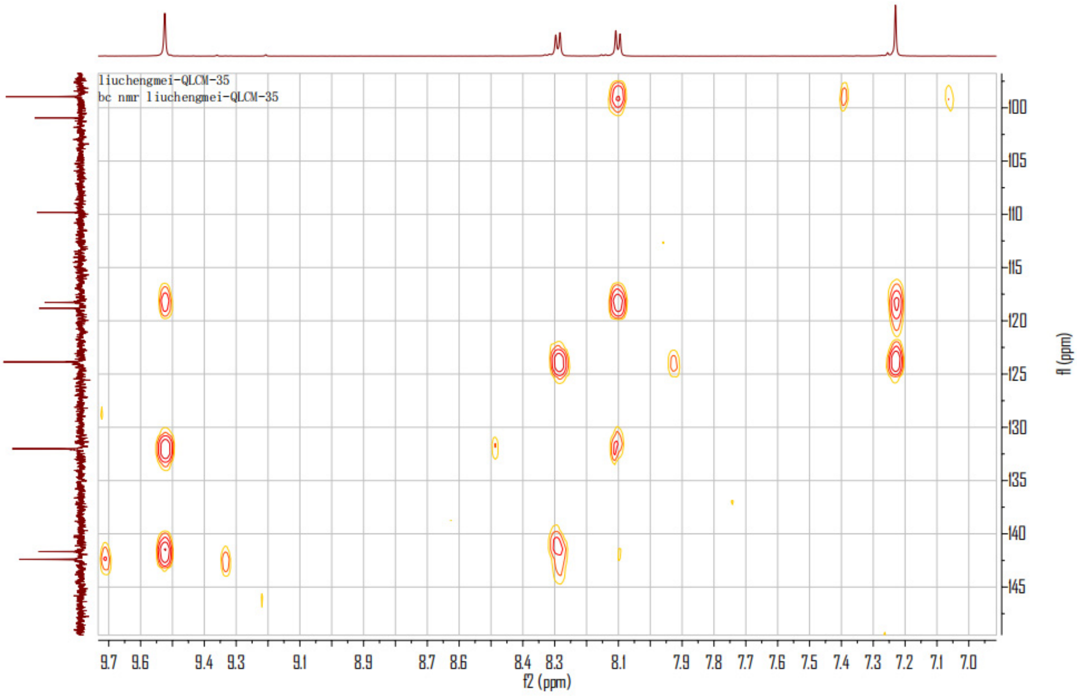

Figure S110. Partial HMBC spectrum of **13** in methanol-*d*4

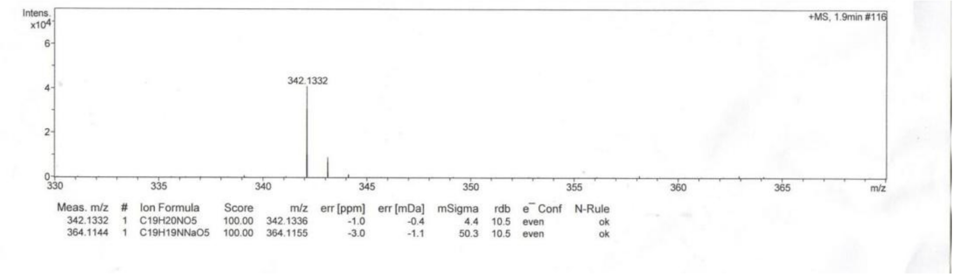

Figure S111. HRESIMS spectrum of **13**

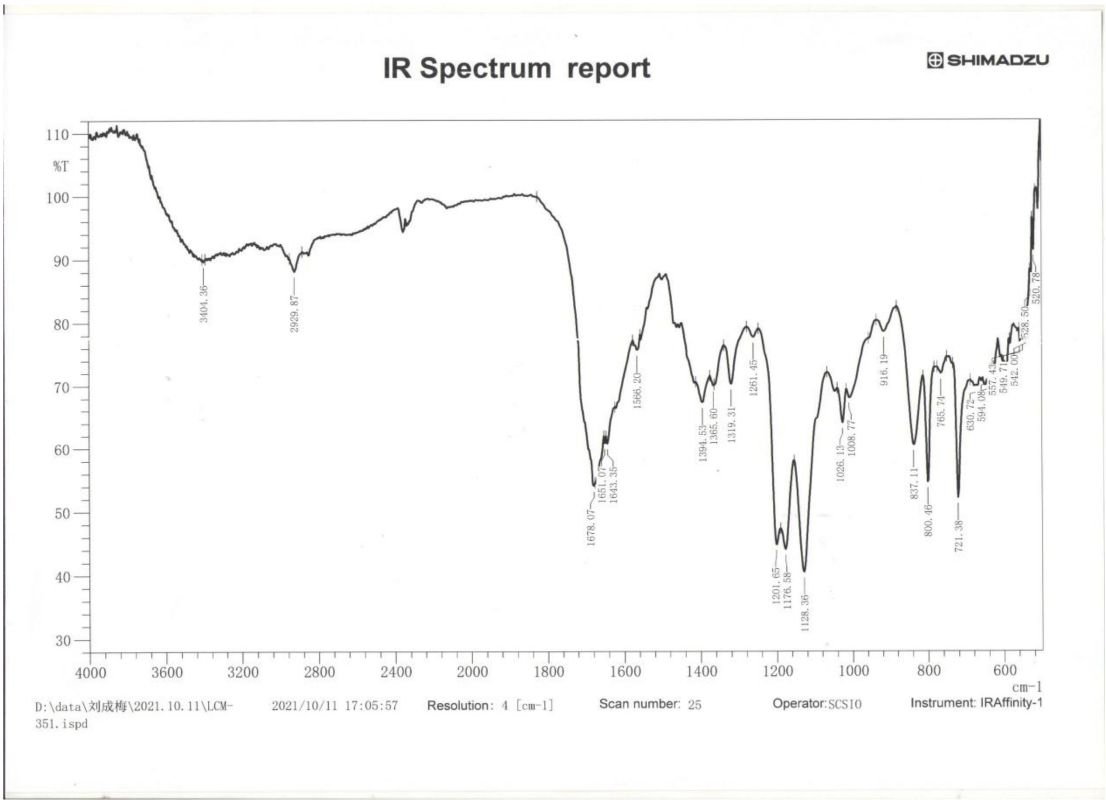

Figure S112. IR spectrum of **13**

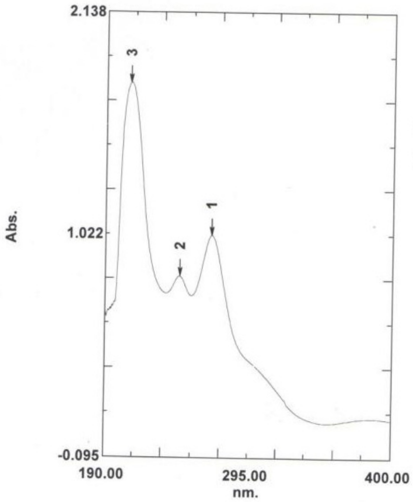

Figure S113. UV spectrum of **13.**

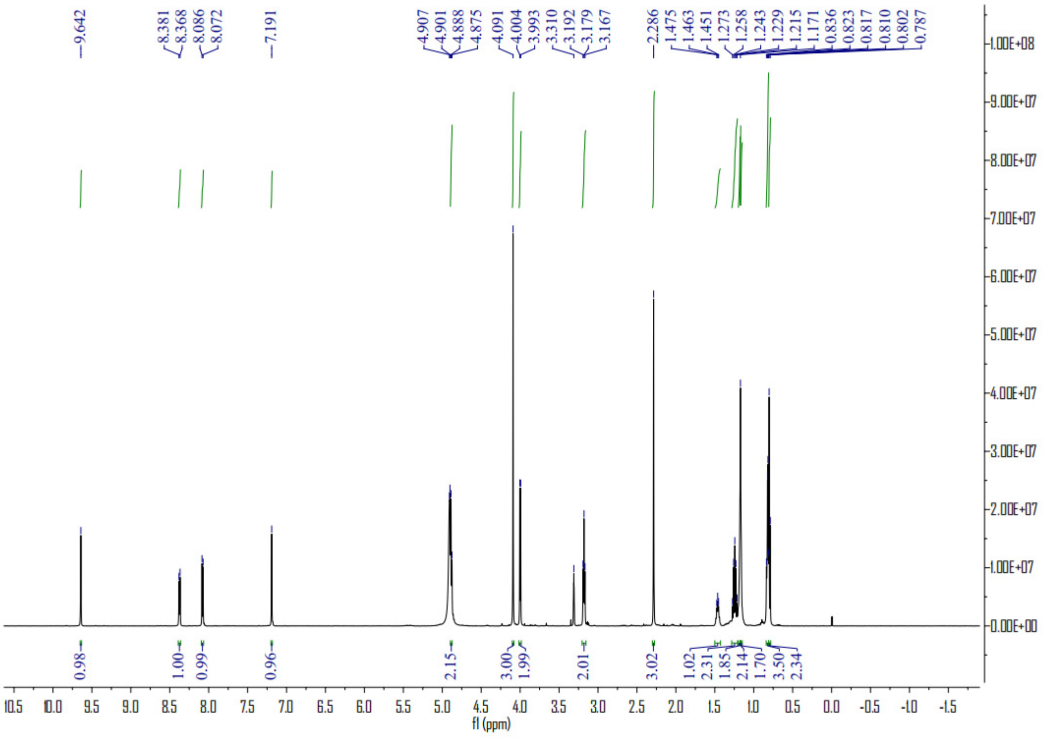

Figure S114. ^{1}H NMR spectrum of **14** in methanol-d_4

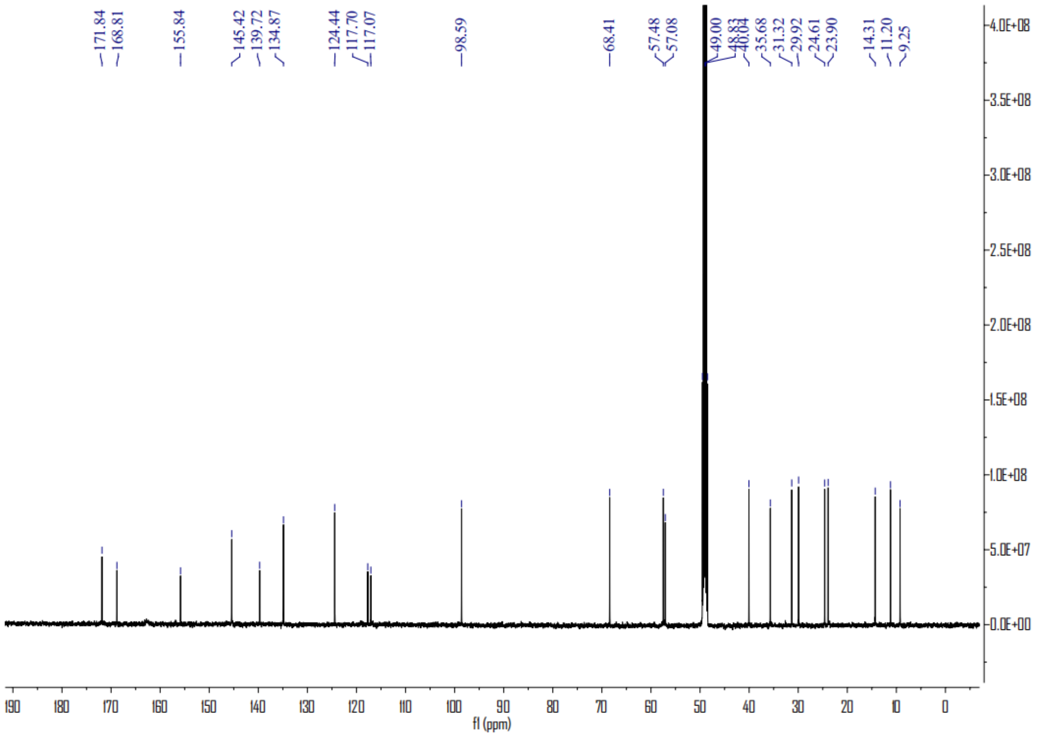

Figure S115. ^{13}C NMR spectrum of **14** in methanol-d_4

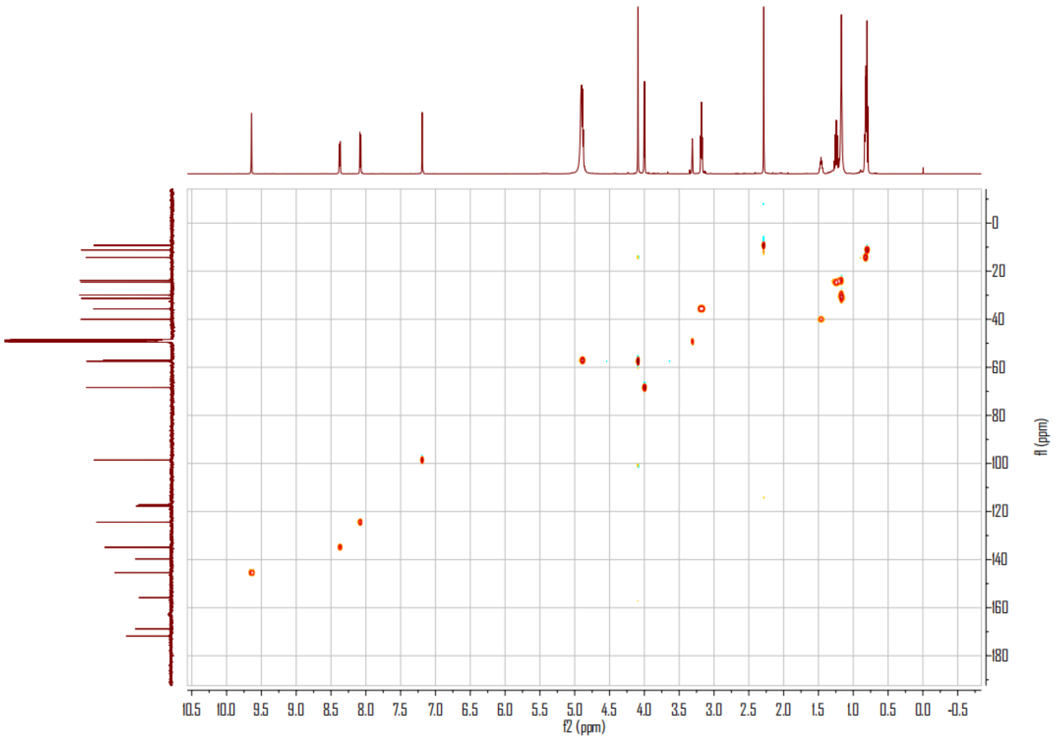

Figure S116. HSQC spectrum of **14** in methanol-d_4

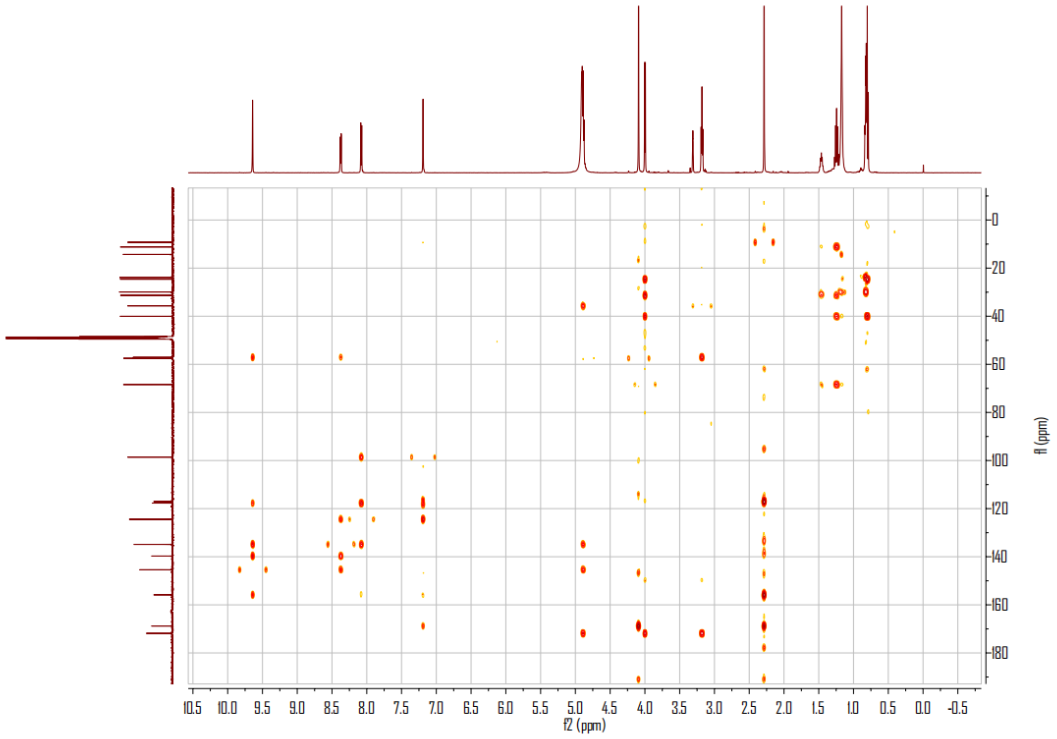

Figure S117. HMBC spectrum of **14** in methanol-d_4

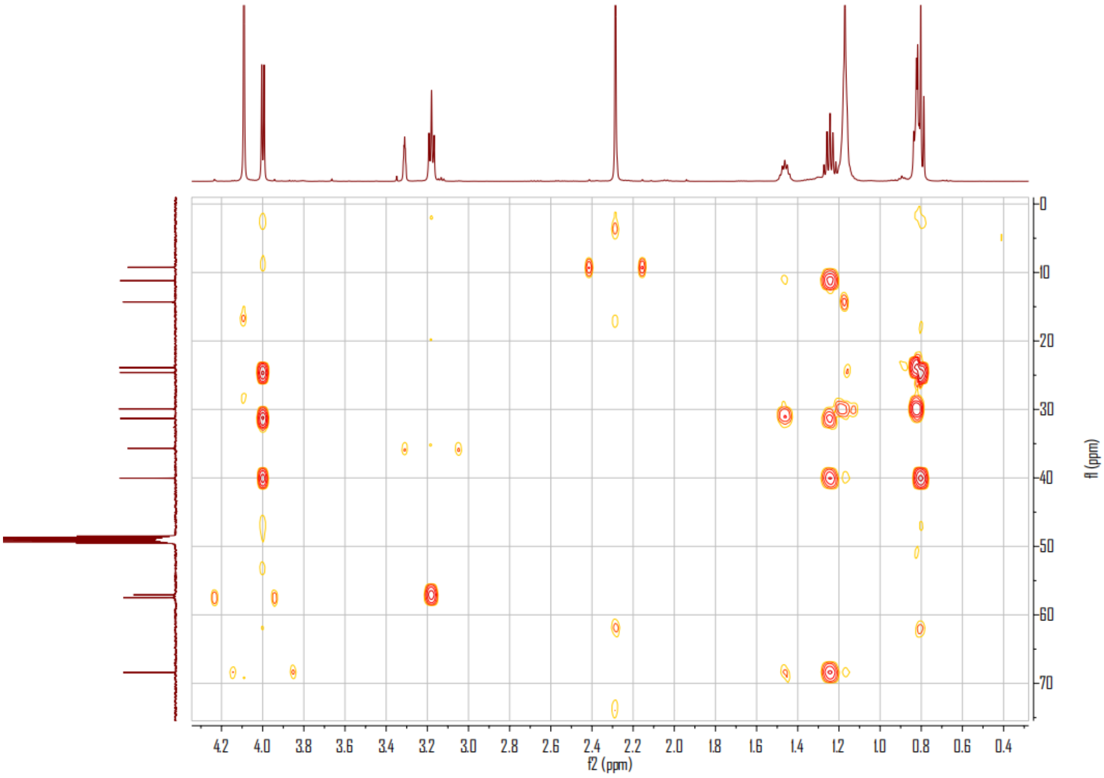

Figure S118. Partial HMBC spectrum of **14** in methanol-d_4

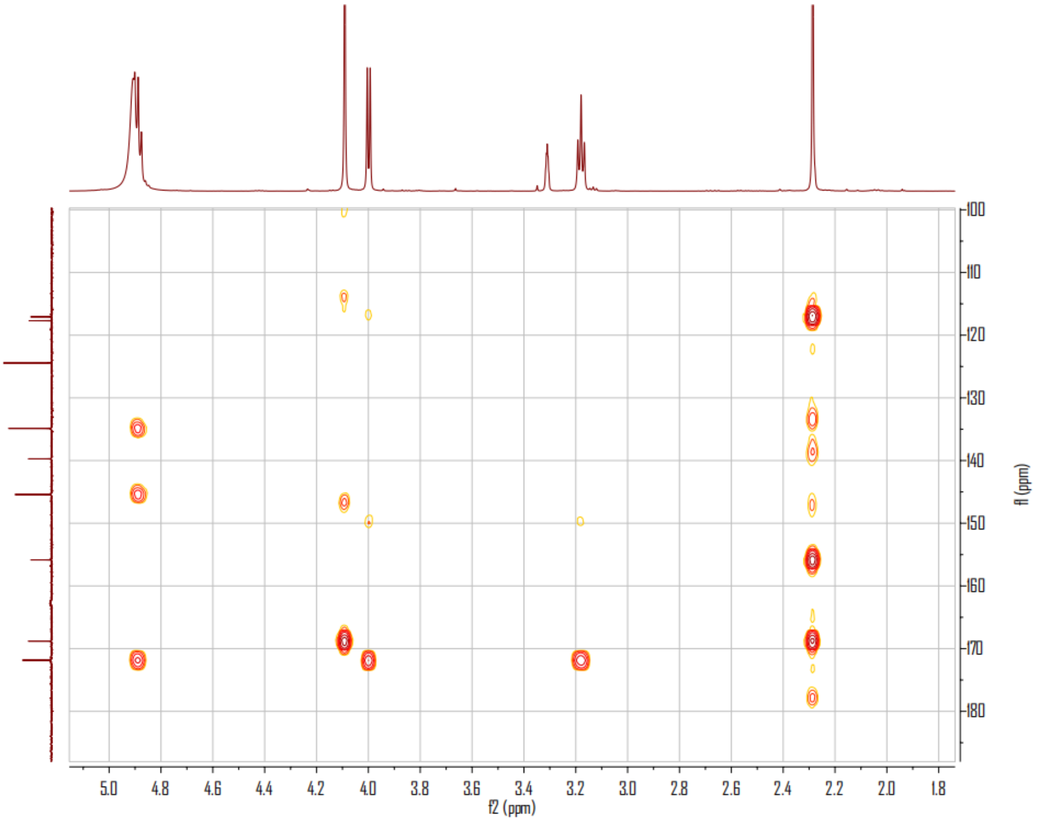

Figure S119. Partial HMBC spectrum of **14** in methanol-d_4

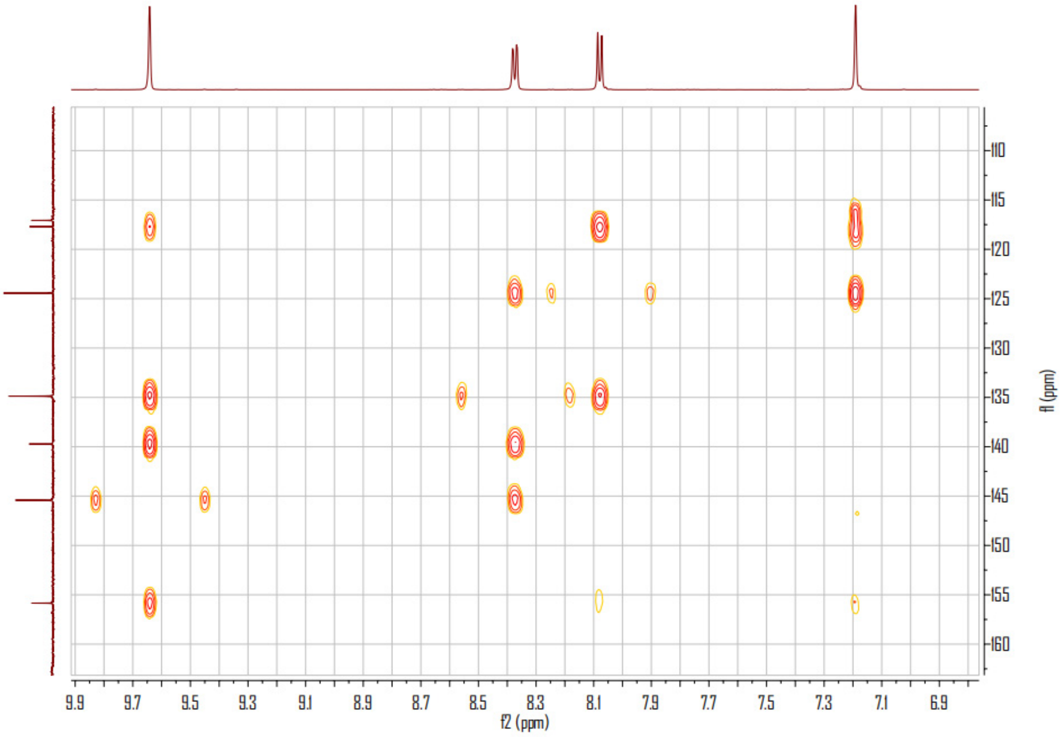

Figure S120. Partial HMBC spectrum of **14** in methanol-*d*4

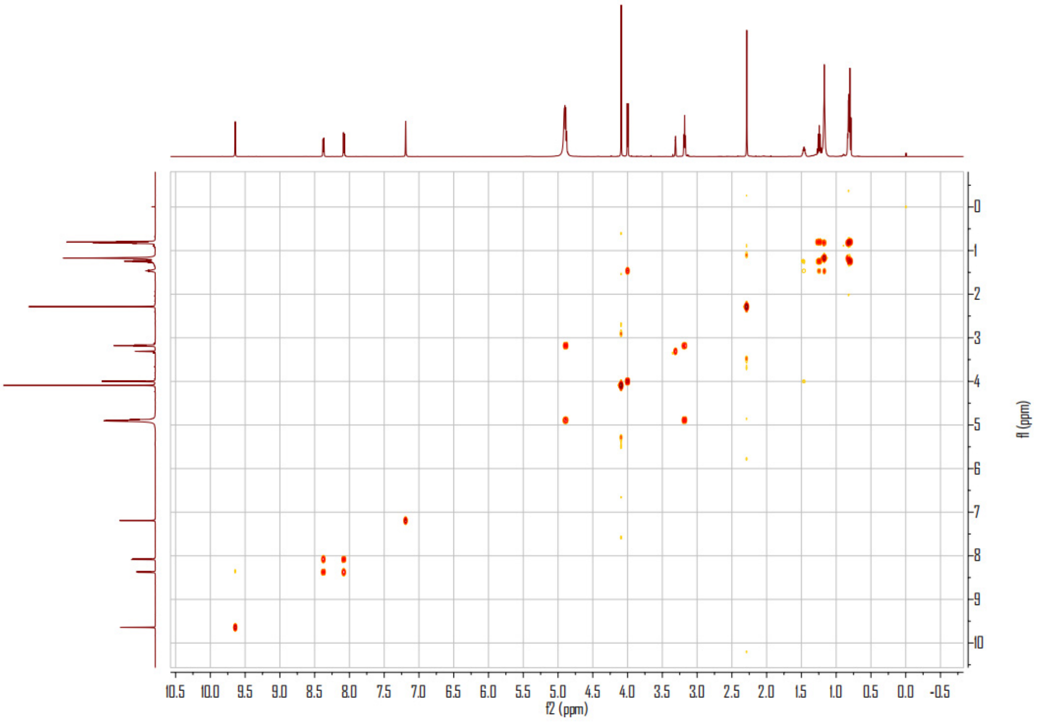

Figure S121. COSY spectrum of **14** in methanol-*d*4

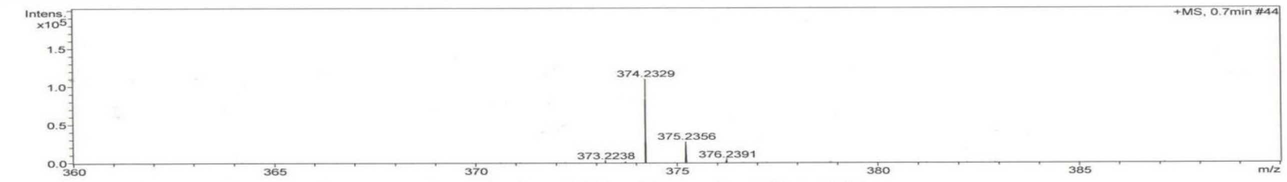

Meas. m/z	#	Ion Formula	Score	m/z	err [ppm]	err [mDa]	mSigma	rdb	e⁻ Conf	N-Rule
374.2329	1	C22H32NO4	100.00	374.2326	-0.7	-0.3	2.3	7.5	even	ok
396.2164	1	C22H31NNaO4	-1.#J	396.2145	-4.8	-1.9	n.a.	7.5	even	ok

Figure S122. HRESIMS spectrum of **14**

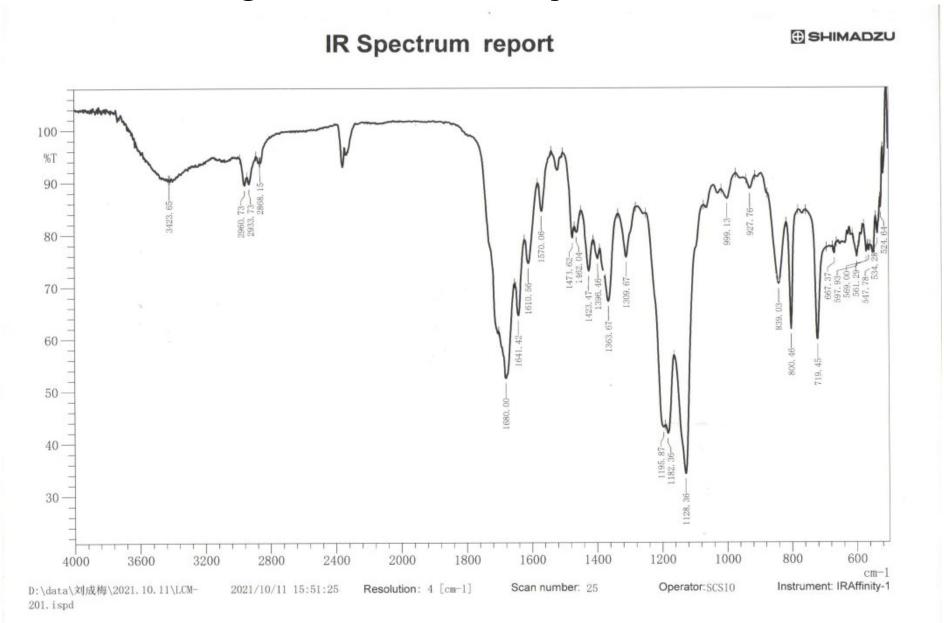

Figure S123. IR spectrum of **14**

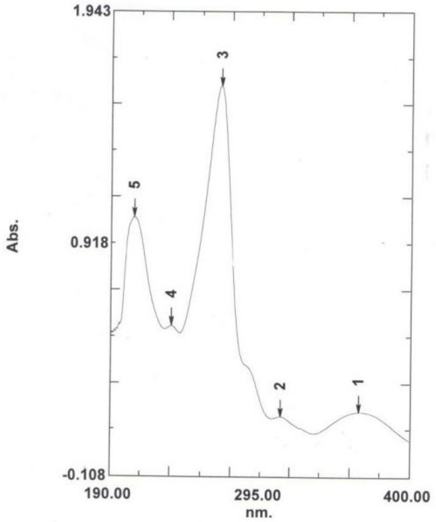

Figure S124. UV spectrum of **14**

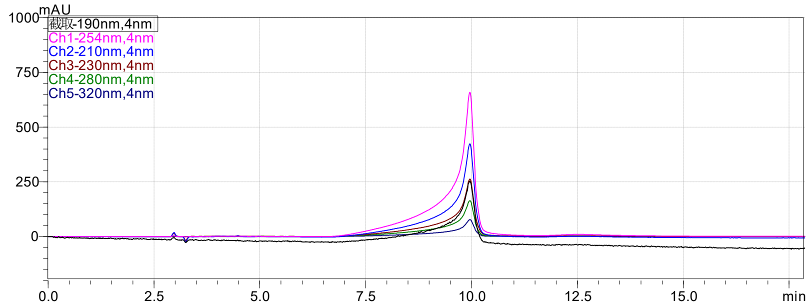

Figure S125. Chirality analysis of compound **14** by CHIRALPAK IA

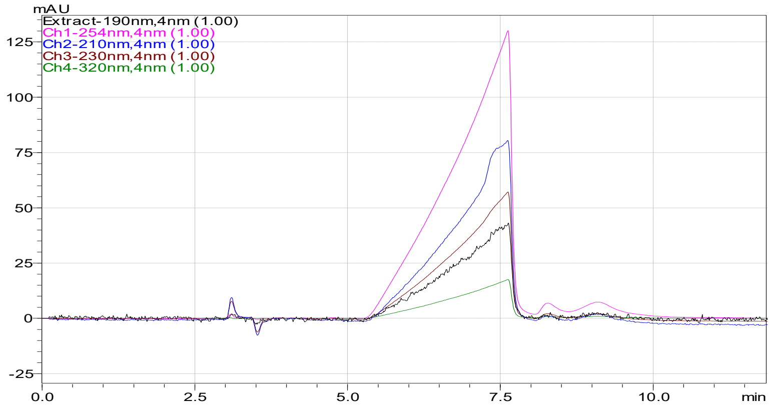

Figure S126. Chirality analysis of compound **14** by CHIRALPAK IB

Table S1. Stable conformers of compound (16*R*)-**9** used for ECD calculations

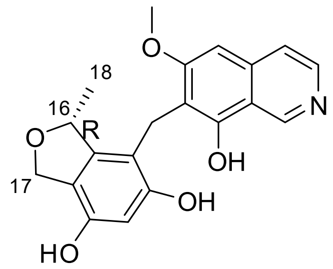

(16*R*)-**9**

Conformer	Conformation
**9**-1	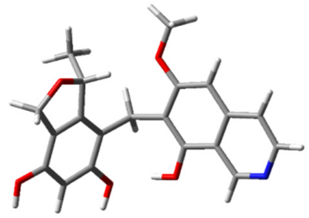
**9**-2	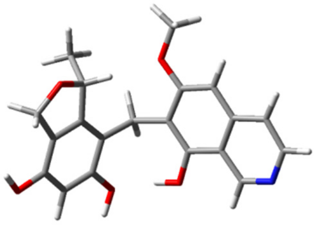
**9**-3	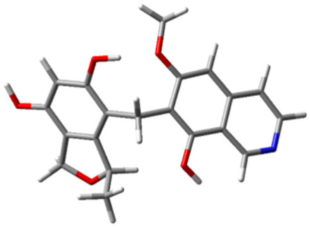
**9**-4	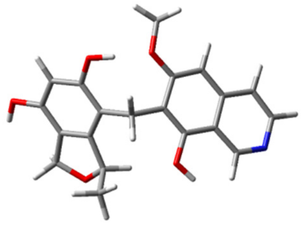
**9**-5	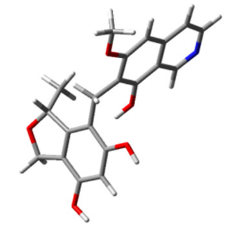

**9**-6	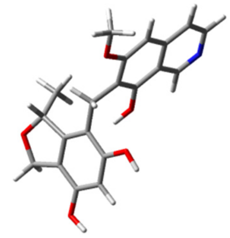
**9**-7	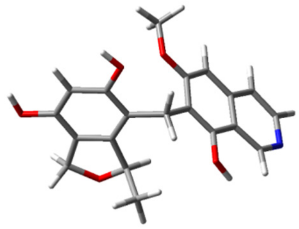
**9**-8	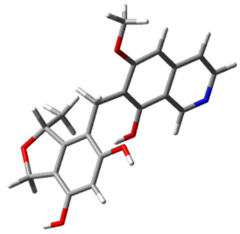
**9**-9	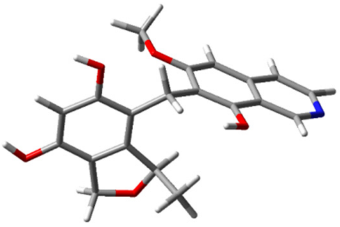
**9**-10	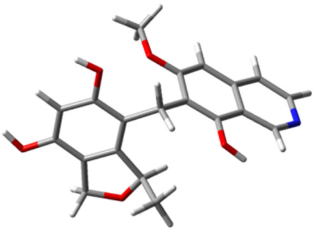
**9**-11	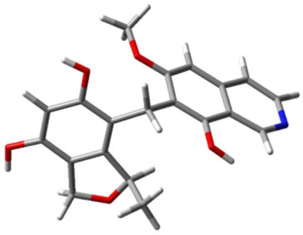

Table S2. Important thermodynamic parameters (a.u.) of (16*R*)-**9** at B3LYP/6-311G (d,p) level in the gas phase

Conformer	*E(*kcal/mol)	*H(*kcal/mol)	*G(*kcal/mol)	imaginary frequencies
**9-**1	-755574.960	-755574.367	-755621.613	18.87
**9-**2	-755574.377	-755573.784	-755621.078	18.75
**9-**3	-755572.438	-755571.846	-755619.745	20.41
**9-**4	-755571.803	-755571.211	-755619.772	15.55
**9**-5	-755572.743	-755572.150	-755620.652	15.98
**9**-6	-755572.743	-755572.150	-755620.653	15.97
**9-**7	-755572.269	-755571.677	-755620.579	10.81
**9-**8	-755572.108	-755571.516	-755620.141	15.61
**9**-9	-755571.950	-755571.357	-755619.951	12.11
**9**-10	-755572.268	-755571.675	-755620.573	10.82
**9**-11	-755571.750	-755571.157	-755620.099	12.20

E: total energy; *H*: enthalpy; *G*: Gibbs free energy.

Table S3. Relative thermal energies (ΔE), relative free energies (ΔG), and equilibrium populations (P) of low-energy conformers of structures (16*R*)-**9** in methanol.

Conformer	*ΔE(*kcal/mol)[a]	*ΔG(*kcal/mol)[a]	P_G%[b]
**9-**1	0.000	0.000	40.71
**9-**2	0.583	0.535	16.50
**9-**3	2.522	1.868	1.74
**9**-4	3.157	1.841	1.82
**9**-5	2.217	0.961	8.03
**9**-6	2.217	0.960	8.05
**9**-7	2.691	1.034	7.11
**9**-8	2.852	1.472	3.39
**9**-9	3.010	1.662	2.46
**9**-10	2.692	1.040	7.03
**9**-11	3.210	1.514	3.16

[a] At the M062X/def2TZVP scrf(SMD) level of theory.
[b] From Δ*G* values at 298.15 K.

Table S4. Crystal data and structure refinement for compound **6**

Identification code	lcm-16
Empirical formula	$C_{14}H_{14.04}NO_4$
Formula weight	260.30
Temperature/K	100.00(10)
Crystal system	monoclinic
Space group	$P2_1/c$
a/Å	4.82780(10)
b/Å	13.4963(3)
c/Å	22.6665(7)
α/°	90
β/°	96.025(3)
γ/°	90
Volume/Å^3	1468.73(6)
Z	4
ρ_{calc}g/cm^3	1.177
μ/mm^{-1}	0.724
F(000)	548.0
Crystal size/mm^3	0.15 × 0.12 × 0.1
Radiation	Cu Kα (λ = 1.54184)
2Θ range for data collection/°	7.634 to 147.79
Index ranges	-5 ≤ h ≤ 5, -16 ≤ k ≤ 9, -27 ≤ l ≤ 26
Reflections collected	7185
Independent reflections	2852 [R_{int} = 0.0197, R_{sigma} = 0.0255]
Data/restraints/parameters	2852/1/212
Goodness-of-fit on F^2	1.073
Final R indexes [I>=2σ (I)]	R_1 = 0.0487, wR_2 = 0.1366
Final R indexes [all data]	R_1 = 0.0545, wR_2 = 0.1406
Largest diff. peak/hole / e Å^{-3}	0.48/-0.26

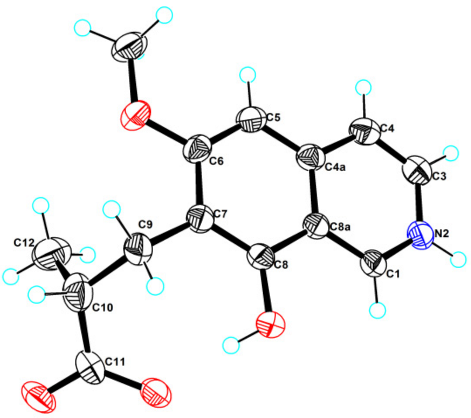

Table S5. Crystal data and structure refinement for compound **12**

.

Identification code	LCM-28
Empirical formula	$C_{18}H_{17}NO_5$
Formula weight	327.32
Temperature/K	100.00(10)
Crystal system	triclinic
Space group	P-1
a/Å	7.6037(4)
b/Å	9.9253(5)
c/Å	10.5261(6)
α/°	70.543(5)
β/°	85.628(4)
γ/°	81.605(4)
Volume/Å^3	740.68(7)
Z	2
ρ_{calc}g/cm^3	1.468
μ/mm^{-1}	0.897
F(000)	344.0
Crystal size/mm^3	0.13 × 0.12 × 0.1
Radiation	Cu Kα (λ = 1.54184)
2Θ range for data collection/°	8.914 to 148.202
Index ranges	$-9 \leq h \leq 9$, $-7 \leq k \leq 12$, $-11 \leq l \leq 13$
Reflections collected	7019
Independent reflections	2880 [R_{int} = 0.0259, R_{sigma} = 0.0317]
Data/restraints/parameters	2880/0/220
Goodness-of-fit on F^2	1.082
Final R indexes [I>=2σ (I)]	R_1 = 0.0419, wR_2 = 0.1114
Final R indexes [all data]	R_1 = 0.0523, wR_2 = 0.1150
Largest diff. peak/hole / e Å^{-3}	0.21/-0.27

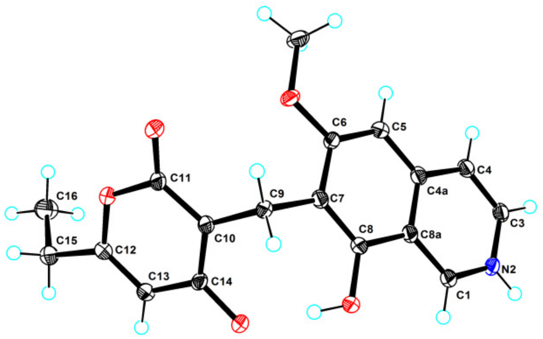

Supplement: Supplementary file 1 [file marinedrugs-20-00078-s001.zip › marinedrugs-1548956-supplementary.pdf]
